# The aldolase inhibitor aldometanib mimics glucose starvation to activate lysosomal AMPK

**DOI:** 10.1038/s42255-022-00640-7

**Published:** 2022-10-10

**Authors:** Chen-Song Zhang, Mengqi Li, Yu Wang, Xiaoyang Li, Yue Zong, Shating Long, Mingliang Zhang, Jin-Wei Feng, Xiaoyan Wei, Yan-Hui Liu, Baoding Zhang, Jianfeng Wu, Cixiong Zhang, Wenhua Lian, Teng Ma, Xiao Tian, Qi Qu, Yaxin Yu, Jinye Xiong, Dong-Tai Liu, Zhenhua Wu, Mingxia Zhu, Changchuan Xie, Yaying Wu, Zheni Xu, Chunyan Yang, Junjie Chen, Guohong Huang, Qingxia He, Xi Huang, Lei Zhang, Xiufeng Sun, Qingfeng Liu, Abdul Ghafoor, Fu Gui, Kaili Zheng, Wen Wang, Zhi-Chao Wang, Yong Yu, Qingliang Zhao, Shu-Yong Lin, Zhi-Xin Wang, Hai-Long Piao, Xianming Deng, Sheng-Cai Lin

**Affiliations:** 1 State Key Laboratory of Cellular Stress Biology, School of Life Sciences, Faculty of Medicine and Life Sciences, Xiamen University, Fujian, China; 2 Department of Endocrinology and Metabolism, Shanghai Jiao Tong University Affiliated Sixth People’s Hospital, Shanghai, China; 3 Laboratory Animal Research Centre, Xiamen University, Fujian, China; 4 Analysis and Measurement Centre, School of Pharmaceutical Sciences, Xiamen University, Fujian, China; 5 Key Laboratory of Ministry of Education for Protein Science, School of Life Sciences, Tsinghua University, Beijing, China; 6 State Key Laboratory of Molecular Vaccinology and Molecular Diagnostics, School of Public Health, Xiamen University, Fujian, China; 7 CAS Key Laboratory of Separation Science for Analytical Chemistry, Dalian Institute of Chemical Physics, Chinese Academy of Sciences, Liaoning, China

**Keywords:** Metabolic diseases, Drug development, Nutrient signalling

## Abstract

The activity of 5′-adenosine monophosphate-activated protein kinase (AMPK) is inversely correlated with the cellular availability of glucose. When glucose levels are low, the glycolytic enzyme aldolase is not bound to fructose-1,6-bisphosphate (FBP) and, instead, signals to activate lysosomal AMPK. Here, we show that blocking FBP binding to aldolase with the small molecule aldometanib selectively activates the lysosomal pool of AMPK and has beneficial metabolic effects in rodents. We identify aldometanib in a screen for aldolase inhibitors and show that it prevents FBP from binding to v-ATPase-associated aldolase and activates lysosomal AMPK, thereby mimicking a cellular state of glucose starvation. In male mice, aldometanib elicits an insulin-independent glucose-lowering effect, without causing hypoglycaemia. Aldometanib also alleviates fatty liver and nonalcoholic steatohepatitis in obese male rodents. Moreover, aldometanib extends lifespan and healthspan in both *Caenorhabditis elegans* and mice. Taken together, aldometanib mimics and adopts the lysosomal AMPK activation pathway associated with glucose starvation to exert physiological roles, and might have potential as a therapeutic for metabolic disorders in humans.

## Abstract (web summary)

A small-molecule aldolase inhibitor, aldolazin is reported and shown to selectively activate the lysosomal pool of AMPK, which has glucose-lowering effects in rodents.

## Main

AMPK is a pivotal controller of metabolic homeostasis^1–3^. It comprises a heterotrimeric complex of a catalytic α-subunit and regulatory β- and γ-subunits, among which the γ-subunit provides binding sites for the regulatory adenine nucleotides AMP, ADP and ATP, whose occupancy depends on the cellular AMP:ATP and ADP:ATP ratios^4–8^. Depending on cellular glucose levels as well as energy states, AMPK is regulated in a hierarchical spatiotemporal manner^9–12^. Under falling levels of glucose and hence FBP, but before cellular energy becomes limited, the lysosomally localized AMPK is activated via the lysosomal activation axis^13,14^. In this axis, lack of FBP is directly sensed by the lysosomal proton pump vacuolar H^+^-ATPase (v-ATPase)-associated aldolase, which in turn blocks the endoplasmic reticulum (ER)-localized calcium channel transient receptor potential V (TRPV) subfamily, converting low cellular glucose to low calcium signal at the ER–lysosome contact^14,15^. TRPV then interacts with v-ATPase, causing a reconfiguration of the aldolase–v-ATPase complex, resulting in inhibition of v-ATPase^15^. Thus, AXIN utilizes v-ATPase and its associated Ragulator (comprising five LAMTOR subunits, LAMTOR1–5) as docking sites to tether liver kinase B1 (LKB1), an AMPK upstream kinase^13,16^. This leads to AMPK phosphorylation at Thr172 and its activation. Importantly, lysosomal AMPK activation occurs in vivo in various physiological conditions such as starvation and calorie restriction^14,17^. Once cellular energy levels are low, the increased AMP causes allosteric changes in AMPK, which enables it to form a complex with LKB1-bound AXIN in the cytosol independently of the lysosome pathway, leading to activation of the cytosolic pool of AMPK in addition to the lysosomal pool^9,16^. In the case of severe stress from conditions such as extreme starvation and ischaemia, mitochondrial AMPK is also activated, in an AXIN-independent manner^9,18,19^. Once activated, AMPK directly phosphorylates multiple targets to inhibit anabolism and stimulate catabolism, thereby minimizing ATP consumption and stimulating ATP production to maintain energy homeostasis^1,3^. For example, the acetyl-CoA carboxylases (ACC1 and ACC2) are substrates of AMPK for inhibition of fatty acid synthesis and promotion of fatty acid oxidation under glucose/energy stress^20^. AMPK also phosphorylates the transcription factor sterol regulatory element-binding protein (SREBP)-1c to suppress fatty acid synthesis at the transcriptional level^21^. In addition, the activity of AMPK also contributes to the inhibition of target of rapamycin complex 1 (TORC1) and hence protein synthesis by phosphorylating tuberous sclerosis complex and the Raptor subunit of TORC1 under conditions of glucose starvation^22,23^. Apart from blocking anabolic processes, AMPK phosphorylates such substrates as TBC1 domain family member 1 (TBC1D1) to promote glucose uptake in skeletal muscle^24,25^ to increase glycolysis^26^. In addition, AMPK promotes autophagy, either through directly phosphorylating the unc-51-like autophagy activating kinase 1 and Beclin-1, or through the inhibition of TORC1 complex^27–29^. AMPK also exerts a critical role in promoting mitochondrial biogenesis through elevating cellular NAD^+^ levels for activating sirtuins, thus promoting oxidative phosphorylation and maximizing the efficiency of ATP production^30,31^. The roles of AMPK in TORC1 inhibition, autophagy initiation, NAD^+^ elevation and the activation of sirtuins have all been implicated in longevity^32^. These pleiotropic actions suggest that AMPK is a potential therapeutic target for treating metabolic disorders such as diabetes and fatty liver disease^33–35^.

However, despite immense interest and efforts, pharmacological agonists directly targeting AMPK often cause adverse effects, likely due to indiscriminate activation of all subcellular pools of AMPK being unphysiological^36^. For example, the pan-AMPK activator MK-8722 robustly activates AMPK in skeletal muscle and significantly improves glucose homeostasis, but causes cardiac hypertrophy^37^. The mitochondrial inhibitors could activate AMPK via elevating cellular AMP levels, but the inhibition of mitochondria per se is harmful to organisms, particularly under prolonged treatments^38,39^. Metformin, a first-tier drug for treating type 2 diabetes, is known to activate AMPK, but can only target the liver, kidney and intestine but not skeletal muscle (an important tissue for glucose lowering due to its large contribution to body mass)^40–43^, thereby limiting its effects on glucose lowering. Nevertheless, it is rather encouraging to see that the AMPKβ1-specific agonists have shown effects in improving insulin sensitivity and alleviating fatty liver, as well as acceptable safety profiles^44–48^.

## Results

### Aldometanib activates lysosomal AMPK

**Fig. 1:**
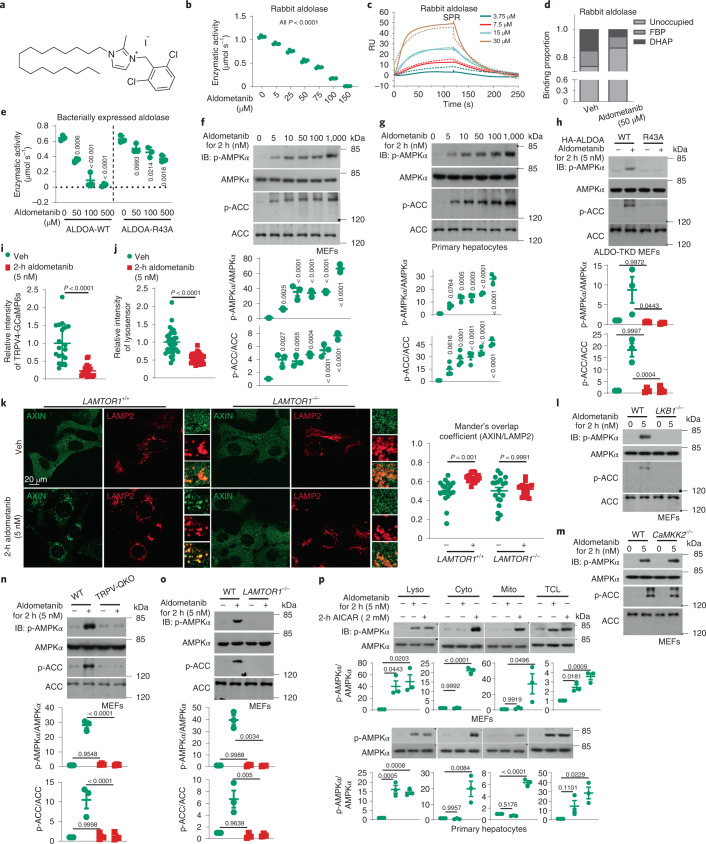
Aldometanib activates lysosomal AMPK. **a**,**b**, Aldometanib inhibits the enzymatic activity of purified aldolase. Enzymatic activities of rabbit aldolase are shown in **b** as the mean ± s.e.m.; *n* = 4 biological replicates for each concentration of aldometanib (see structure in **a**), and *P* values were determined by one-way analysis of variance (ANOVA), followed by Tukey’s test. **c**, Aldometanib is able to bind aldolase. Representative sensorgrams of SPR assays in which aldometanib were incubated with rabbit aldolase immobilized on a CM5 chip (solid line), and fitted curves for each measurement (dashed line) are shown. RU, response unit. **d**, Aldometanib prevents FBP from binding to aldolase. Rabbit aldolase was incubated with aldometanib, followed by determining the potential FBP and dihydroxyacetone phosphate (DHAP) binding by MS as described previously^64^. **e**,**h**, The Arg43 residue of aldolase is required for aldometanib binding. Enzymatic activities of bacterially expressed ALDOA-p.Arg43Ala (**e**), and AMPK activation in ALDO-TKD MEFs reintroduced with ALDOA-p.Arg43Ala (**h**) were determined. Data are the mean ± s.d. (**e**) or s.e.m. (**h**; quantification of immunoblots); *n* = 3 biological replicates for each condition, and *P* values were by one-way (**e**) or two-way (**h**) ANOVA, followed by Tukey’s test (**e** and **h**). **f**,**g**,**l**–**o**, Aldometanib activates AMPK via the lysosomal pathway. Wild-type MEFs (**f**) and MEFs *LKB1*^−/−^ (**l**), *CaMKK2*^−/−^ (**m**), *TRPV1-4*-QKO (**n**), *LAMTOR1*^−/−^ (**o**) or mouse primary hepatocytes (**g**) were treated with aldometanib. Quantification data for immunoblots are the mean ± s.e.m., *n* = 4 (p-ACC/ACC of **g**) or 3 (others) dishes of cells for each condition, with *P* values calculated by two-way ANOVA followed by Tukey’s test (**n** and **o**), or by one-way ANOVA, followed by Tukey’s test (**f** and **g**; comparison between control and aldometanib-treated groups). **i**–**k**, Aldometanib inhibits TRPVs and induces AXIN lysosomal translocation. MEFs were infected with TRPV4-GCaMP6s (**i**), pre-labelled with (**j**) or without (**k**) LysoSensor, and then treated with aldometanib, followed by determining the fluorescence intensity of TRPV4-GCaMP6s (**i**), LysoSensor (**j**) or staining AXIN and the lysosomal marker LAMP2 (**k**). Results are the mean ± s.d. (**i**) or s.e.m. (others); *n* = 19, 35, 18, 20, 19 and 18 cells from 5, 2, 4, 3, 4 and 3 dishes/fields for **i**, **j**, control of wild-type MEFs of **k**, aldometanib treatment of wild-type MEFs of **k**, control of *LAMTOR1*^−/−^ MEFs and aldometanib treatment of *LAMTOR1*^−/−^ MEFs, respectively; and *P* values were determined by two-sided Student’s *t*-test with Welch’s correction (**i** and **j**), or by two-way ANOVA followed by Tukey’s test (**k**). **p**, Aldometanib specifically activates lysosomally localized AMPK. MEFs or primary hepatocytes were treated with aldometanib, or AICAR (representing severe stress^9^), followed by analysis of p-AMPKα in lysosomes (Lyso), cytosol (Cyto), mitochondria (Mito) and total cell lysates (TCL). Quantification data are the mean ± s.e.m., from *n* = 3 biological replicates, with *P* values calculated by one-way ANOVA, followed by Tukey’s test (compared as in **f**). Experiments were performed three times, except in **b** and **e**, which were performed four times. WT, wild type. Source data

**Extended Data Fig. 1:**
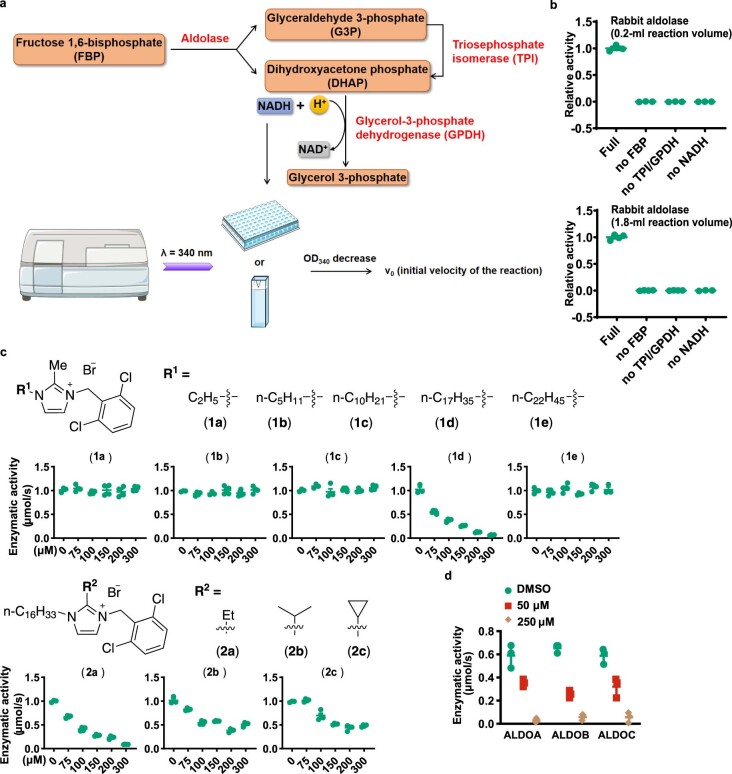
Aldometanib inhibits aldolase in vitro. **a**, Schematic diagram depicting the principle for determining aldolase activity in vitro. The activity of aldolase was determined through its ability to catalyse FBP to G3P and DHAP. The yielded G3P was converted to DHAP through TPI, and was further catalysed to glycerol 3-phosphate through GPDH, coupled with a transition of NADH to NAD^+^. The decreases of OD_340_ of NADH were then quantified through a microplate reader (in 0.2-ml reaction volume, in 96-well plates; for screening assays) or a spectrophotometer (in 1.8-ml reaction volume, in cuvettes; for quantifying absolute activity), and then converted to the changes of amount of NAD^+^ by the Beer-Lambert law for calculating the activity of aldolase. **b**, Validations of aldolase enzymatic assay. Some 50 nM rabbit aldolase (ammonium sulphate precipitates) was incubated with FBP, TPI/GPDH and NADH (labelled as “complete”) in 96-well plates (upper panel) or cuvettes (lower panel), followed by determining its enzymatic activity. See also reactions without FBP (no FBP), TPI/GPDH (no TPI/GPDH), or NADH (no NADH) addition as controls. Results are shown as mean ± s.e.m.; *n* = 4 (“Full”, “no FBP” and “no TPI/GPDH” of lower panel) or 3 (others) biological replicates for each condition. **c**, Structure-activity relationship of 1,3-disubstituted imidazole derivatives. Some 50 nM rabbit aldolase was incubated with indicated chemical compounds (at indicated concentrations) for 30 min, followed by determining the activities of aldolase. Results are mean ± s.e.m.; *n* = 3 (0 μM, 75 μM of 1c, 100 μM of 1b, and 300 μM of 1e), 5 (150 μM of 1b and 1c, 200 μM of 1b, and 75 μM of 1d), or 4 (others) biological replicates for each condition. **d**, aldometanib inhibits all isozymes of aldolase. Some 100 nM bacterially expressed and purified human ALDOA, ALDOB and ALDOC were incubated with aldometanib for 30 min, followed by determining their activities. Results are shown as mean ± s.d.; *n* = 3 biological replicates for each condition; and *P* value by one-way ANOVA followed by Dunnet. Experiments in this figure were performed three times. Source data

**Extended Data Fig. 2:**
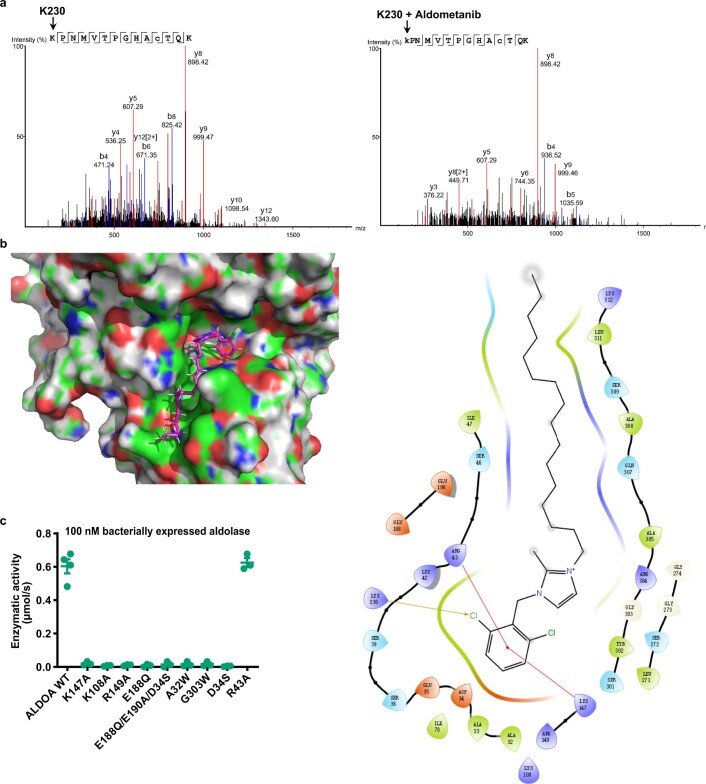
Aldometanib binds aldolase at the K230 residue. **a**, Typical spectrograms of K230 residues with (right) and without (left) aldometanib conjugation. Some 1 μg of rabbit aldolase was incubated with 50 μM aldometanib for 2 h, followed by SDS-PAGE and analysis of covalent modifications through mass spectrometry. **b**, In silico docking results showing that the groove near the K230 residue is required for aldometanib binding. **c**, Enzymatic activities of ALDOA (100 nM, bacterially expressed and purified) carrying mutations on residues that may be responsible for aldometanib binding. Results are shown as mean ± s.d.; *n* = 4 (ALDOA WT) or 3 (others) biological replicates for each condition. Experiments in this figure were performed three times. Source data

We aimed to identify compounds that would mimic conditions of glucose starvation to activate AMPK by inhibiting FBP binding to aldolase. To do so, we set up a drug screening strategy using aldolase as the target in a cell-free enzymatic assays system. The enzymatic activity of aldolase served as the readout and was monitored by changes of absorbance at 340 nm of NADH yielded during the conversion of FBP to phosphotrioses (depicted in Extended Data Fig. 1a, and validated in Extended Data Fig. 1b). After screening an in-house compound library (~5,000 compounds compiled based on chemical scaffolds), we identified 1,3-disubstituted imidazole derivatives as preliminary hits meeting the criteria of inhibiting at least 50% activity of aldolase (ALDOA, used as a representative isozyme of aldolase). Through structure–activity optimization, a compound (LXY-05-029, referred to as aldometanib hereafter) was identified as having the best inhibitory activity, with half-maximal inhibitory concentration (IC_50_) values of approximately 50 μM (Fig. 1a,b and Extended Data Fig. 1c,d; see Supplementary Information for synthesis procedures of LXY-05-029). Surface plasmon resonance (SPR) assays gave a dissociation constant (*K*_D_) value of approximately 20 μM for aldometanib binding to ALDOA, which is within a close range to the Michaelis constant (*K*_m_) value of FBP for ALDOA^49^ (Fig. 1c). Mass spectrometry (MS) analysis showed that aldometanib blocked FBP by directly occupying the active centre of aldolase through conjugation to Lys230 (a critical residue that resides in the active centre of aldolase for FBP binding; see ref. ^49^), as evidenced by an increase in the molecular weight of the lysine residue of 466 Da, equivalent to that of aldometanib (Fig. 1d and Extended Data Fig. 2a). We also performed an in silico docking study^50–52^ according to a previously reported structure of aldolase (PDB ID: 1ZAH)^53^ to predict the residue(s) of aldolase that may participate in aldometanib binding. The results of docking (Extended Data Fig. 2b) indicate that aldometanib may bind the region around the Lys230 residue of aldolase through its phenyl ring and imidazole ring groups (which orient the catalytic centre), and its alkyl chain (extends along the groove on the surface of aldolase). The amino acid residues around the catalytic centre of aldolase that may participate in aldometanib binding are: (a) Lys147, Arg149 and Lys108, which may interact with the phenyl group of aldometanib through their amino groups, forming the cation–π interaction (mutation of them to alanine would block this effect); (b) Glu188, Glu190 and Asp34, which may facilitate the protonation of Lys147, Arg149 and Lys108 through their carboxyl groups, which promotes the cation–π interaction (mutation of Glu188, Glu190 and Glu35 to glutamine will block this effect); and (c) Ala32 and Gly303, which are located near the site of aldometanib binding (mutation of them to tryptophan would spatially interfere aldometanib binding). However, mutations of residues mentioned above also disrupt the processes of aldolase for catalysing FBP, as reported previously^54–58^, which is also consistent with our results of enzymatic assays (Extended Data Fig. 2c). An exception was with Arg43, which forms a cation–π interaction (via its amino group) with aldometanib, further augmenting aldometanib binding to aldolase. Indeed, we found that mutation of Arg43 to alanine largely blocked the effects of aldometanib in aldolase inhibition as well as AMPK activation (Fig. 1e,h), without significantly affecting the activity of aldolase (Extended Data Fig. 2c). A kinome screening assay demonstrated that aldometanib did not show any considerable inhibition (inhibitory rate > 30%) on kinases examined (Supplementary Table 1).

**Extended Data Fig. 3:**
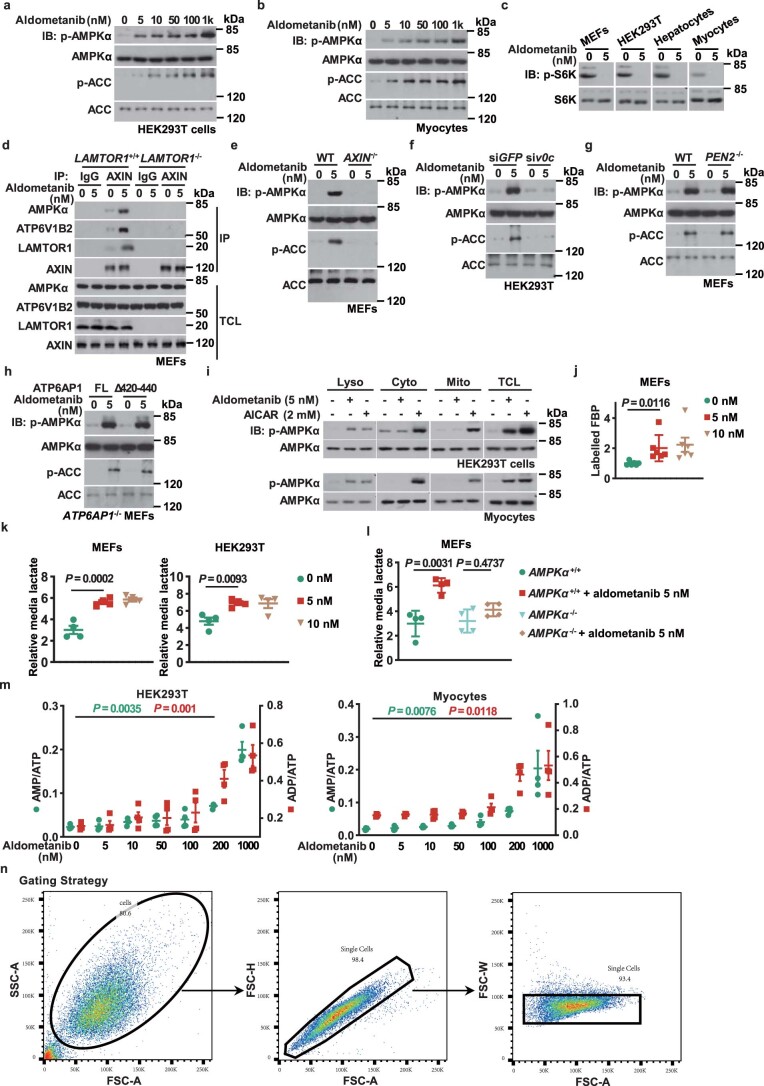
Aldometanib activates the lysosomal pool of AMPK. **a**, **b**, **e-h**, aldometanib activates AMPK via the lysosomal pathway. HEK293T cells (**a**), mouse primary myocytes (**b**), *AXIN*^-/-^ MEFs (**e**), HEK293T cells with *ATP6v0c* knocked down (**f**), *PEN2*^-/-^ MEFs (**g**), or *ATP6AP1*^-/-^ MEFs with the Δ420-440 mutant of ATP6AP1 re-introduced (**h**) were treated with aldometanib at indicated concentrations for 2 h, and the levels of p-AMPKα and p-ACC were determined by immunoblotting. **c**, aldometanib inhibits mTORC1. MEFs, HEK293T, primary hepatocytes and primary myocytes were treated with 5 nM aldometanib for 2 h, and the activity of mTORC1, as assessed by the phosphorylation levels of its substrate S6K (p-S6K), was determined through immunoblotting. **d**, **i**, aldometanib triggers the lysosomal pathway. MEFs were treated as in **c**, followed by immunoprecipitation (IP) of AXIN, or purification of lysosome (as in Fig. 1p). **j**-**l**, aldometanib does not inhibit, and even slightly enhances glycolysis. Wildtype MEFs (**j**, **k**), *AMPKα*^-/-^ MEFs (**l**), and HEK293T cells (**k**) were treated with aldometanib for 2 h (**j**) or 4 h (**k**, **l**), followed by determining glycolytic rates, either through labelled metabolites tracing [**j**, using the levels of labelled (M + 6) FBP as an indicator of glycolytic rate] or lactate production (**k**). Results are shown as mean ± s.d.; *n* = 6 (**j**) or 4 (**k**, **l**) dishes of cells for each condition; and *P* value by one-way ANOVA followed by Dunn (**j**) or Tukey (**k**), or by two-way ANOVA, followed by Tukey (**l**). **m**, aldometanib activates AMPK without elevation of AMP/ADP. HEK293T cells (left panel) or primary myocytes (right panel) were treated with aldometanib at indicated concentrations for 2 h, followed by determining the AMP:ATP and ADP:ATP ratios. Results are shown as mean ± s.e.m.; *n* = 4 dishes of cells for each treatment, and *P* value by one-way ANOVA followed by Dunnet (HEK293T) or Dunn (myocytes). **n**, The gating strategy for quantifying the populations of FITC-labelled cells. Experiments in this figure were performed three times, except **k** and **l** four times. Source data

We next examined the effects of aldometanib on AMPK activation, and found that aldometanib, at concentrations as low as 5 nM, was able to activate AMPK in HEK293T cells, mouse embryonic fibroblasts (MEFs), mouse primary hepatocytes and mouse primary skeletal muscle cells, as determined by phosphorylation of Thr172 of AMPKα and phosphorylation of Ser79 of ACC1 (Fig. 1f,g and Extended Data Fig. 3a,b). We also observed a decrease of phosphorylation of the mammalian/mechanistic TORC1 (mTORC1) substrate ribosomal S6 kinase (S6K) in aldometanib-treated cells, indicating that aldometanib also inhibits mTORC1 (Extended Data Fig. 3c). Mutations of the residues of aldolase that are required for binding or conjugation to aldometanib abrogated AMPK activation by the drug (Fig. 1h). We then determined if aldometanib indeed relies on the lysosomal pathway to activate AMPK, which is known to be triggered by FBP-free aldolase. We found that aldometanib inhibited the activity of TRPV, as the fluorescence from the indicator TRPV4-GCaMP6s^15^ decreased after aldometanib treatment (Fig. 1i). Aldometanib also inhibited v-ATPase, as determined by the signal from LysoSensor Green DND-189 dye, which decreases with increased lysosomal pH (refs. ^15,59^ and Fig. 1j), allowing AXIN/LKB1 to translocate to the lysosome and form a complex with the v-ATPase–Ragulator complex (Fig. 1k and Extended Data Fig. 3d). Knockout of *Lkb1* (*Stk11*) blocked the activation of AMPK by aldometanib (Fig. 1l), but not knockout of *Camkk2*, which is another upstream kinase of AMPK required for bulk calcium^60,61^ but not for the lysosomal pathway-induced AMPK activation^13^ (Fig. 1m). For the other factors involved in the lysosomal pathway, we found that knockout of TRPVs (*Trpv1*, *Trpv2*, *Trpv3* and *Trpv4* in MEFs, leaving those cells with little expression of other TRPVs as validated previously^15^), *Axin* (*Axin1*) or *Lamtor1*, or knockdown of the v0c subunit of v-ATPase (*ATP6v0c*), all blocked the activation of AMPK by aldometanib (Fig. 1k,n,o and Extended Data Fig. 3e,f), ascertaining that aldometanib utilizes the lysosomal components to activate AMPK. In comparison, PEN2, which comprises a separate incoming signalling route for metformin to intersect v-ATPase via the ATP6AP1 subunit for AMPK activation^62^, is not responsible for the aldometanib-mediated AMPK activation (Extended Data Fig. 3g,h). Cell fractionation assays showed that only the lysosomal AMPK was activated in these cells (Fig. 1p and Extended Data Fig. 3i), consistent with glucose starvation being unable to activate other pools of AMPK^9^.

### Aldometanib lowers blood glucose

**Fig. 2:**
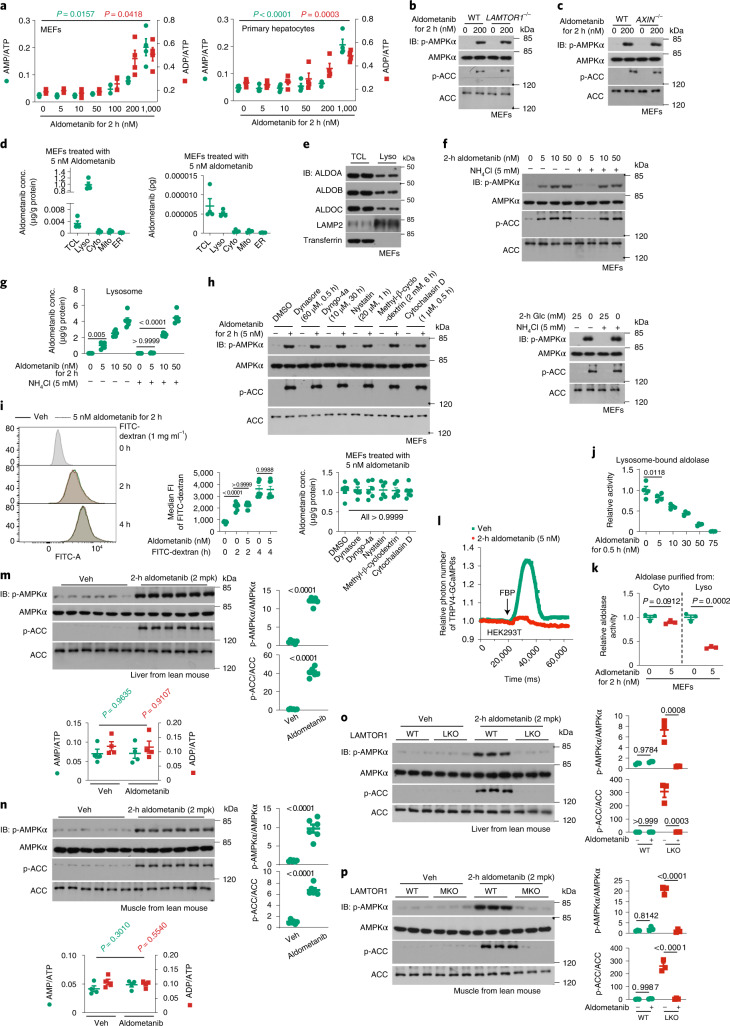
Aldometanib is accumulated in the lysosome. **a**–**c**, Aldometanib activates AMPK through the lysosomal pathway without causing elevation of AMP/ADP, unless high doses are used. In **a**, results of adenylate ratios are shown as the mean ± s.e.m.; *n* = 4 dishes of cells for each condition. *P* values for AMP:ATP (coloured in green, and same hereafter for all comparisons between ratios of AMP:ATP) or ADP:ATP (coloured in red, and same hereafter for ADP:ATP) ratios were determined by one-way ANOVA followed by Dunnet’s (MEFs) or Tukey’s (hepatocytes) test. **d**,**e**,**k**,**l**, Aldometanib is enriched in the lysosome. MEFs (**d**, **e** and **k**) or HEK293T cells (**l**; expressing TRPV4-GCaMP6s indicator) were treated with aldometanib, followed by fractionation as in Fig. 1p to analyse aldometanib contents and concentrations (**d**; see also the lysosomal protein levels of ALDOA, ALDOB and ALDOC in **e**) and the activity of aldolase (**k**) in the lysosomal fraction, or determining the activity of TRPV4, which is an outcome of lysosomal aldolase activity in situ^15^ (**l**). Data are the mean ± s.e.m. from *n* = 3 (**k**) or 4 (**d**) biological replicates for each treatment; *P* values were determined by two-sided Student’s *t*-test (**k**), or by one-way ANOVA followed by Tukey’s test (**d**). **f**–**i**, Lysosomal pH, but not endocytosis, contributes to the accumulation of aldometanib in the lysosome. MEFs were pretreated with NH_4_Cl, followed by incubation with aldometanib or starved for glucose (**f** and **g**). AMPK activation (**f**, upper part of **h**) and lysosomal concentrations of aldometanib (**g**, lower panel of **h**) were then determined. Aldometanib does not affect endocytosis, as evidenced by the fluorescence intensity (FI) of FITC in MEFs labelled with FITC-dextran via flow cytometry (**i**). Data are the mean ± s.e.m., from *n* = 7 (**i**) or 6 (**h**) biological replicates, with *P* values calculated by two-way ANOVA, followed by Tukey’s test (**i**), or by one-way ANOVA, followed by Dunn’s test (**h**). **j**, Intact lysosomes enhance the potency of aldometanib towards aldolase. Lysosomes from MEFs were purified, followed by incubation with aldometanib. Activities of lysosome-bound aldolase are the mean ± s.e.m. from *n* = 4 biological replicates for each treatment; *P* values were determined by one-way ANOVA, followed by Tukey’s test. **m**–**p**, Aldometanib activates AMPK in liver and muscle through the lysosomal pathway. Wild-type mice (**m** and **n**) or mice with *Lamtor1* specifically knocked out in liver (**o**; LKO) or muscle (**p**; MKO) were orally gavaged with aldometanib. After 2 h of gavaging, AMPK activation and the AMP:ATP and ADP:ATP ratios were determined. Data are the mean ± s.e.m. from *n* = 6 (**m** and **n**; AMPK activation), 3 (**o** and **p**; AMPK activation) or 4 (**m** and **n**; adenylate ratios) biological replicates for each treatment; *P* values were determined by two-sided Student’s *t*-test (**m** and **n**), or by two-way ANOVA followed by Tukey’ test (**o** and **p**). Experiments were performed three times. Source data

**Extended Data Fig. 4:**
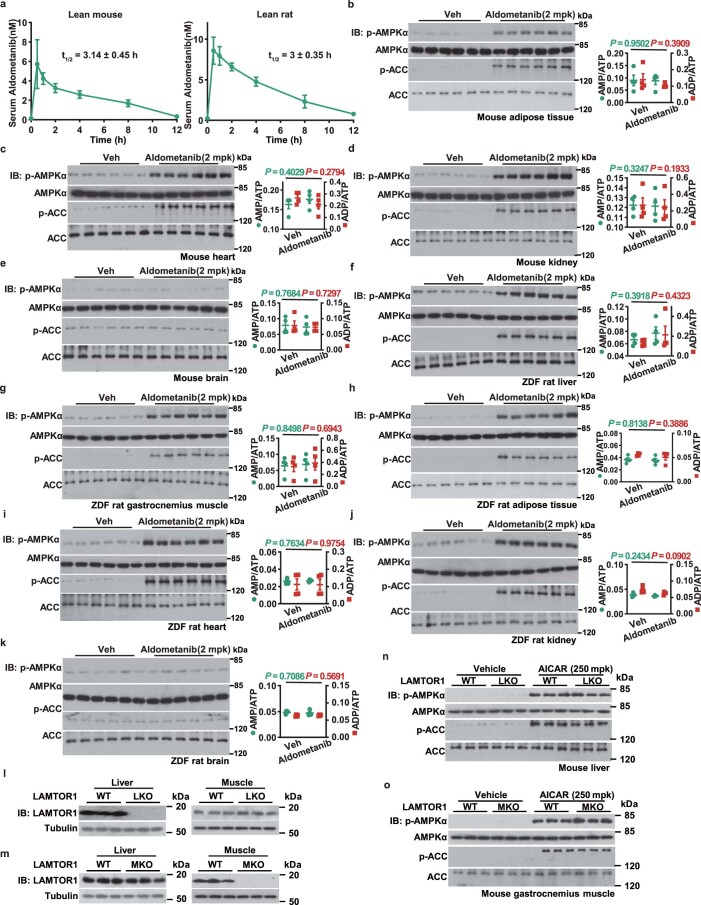
Aldometanib activates AMPK in rodent tissues. **a**, Serum aldometanib concentrations in lean mice and rats. Serum samples were collected at indicated time points from mice (left panel) or rats (right panel) after oral gavaging 2 mpk aldometanib. Data are shown as mean ± s.d.; *n* = 7 mice/rats. See also half-life (t_1/2_) of aldometanib on each panel. **b**-**e**, aldometanib activates AMPK in various mouse tissues. Mice were orally gavaged with 2 mpk aldometanib. Adipose (**b**), heart (**c**), kidney (**d**) or brain (**e**) tissues were collected by freeze-clamping after 1 h of aldometanib gavage, followed by determining AMPK activation (left panel), and AMP:ATP and ADP:ATP ratios (right panel; results are shown as mean ± s.e.m.; *n* = 4 mice for each treatment, and *P* value by two-sided Student’s *t*-test, except ADP:ATP ratios in **b**, **d** by two-sided Student’s *t*-test with Welch’s correction). **f**-**k**, aldometanib activates AMPK in various rat tissues. Rats were orally gavaged with 2 mpk aldometanib. Liver (**f**), gastrocnemius muscle (**g**), adipose (**h**), heart (**i**), kidney (**j**) or brain (**k**) tissues were collected by freeze-clamping after 1 h of aldometanib gavage, followed by determining AMPK activation (left panel) and AMP:ATP and ADP:ATP ratios (right panel; results are shown as mean ± s.e.m.; *n* = 4 rats for each treatment, and *P* values by two-sided Student’s *t*-test, except ADP:ATP ratios in **f** by two-sided Student’s *t*-test with Welch’s correction). **l**, **m**, Validation of knockout efficiency and specificity of LAMTOR1 in *LAMTOR1*-LKO (**l**) and *LAMTOR1*-MKO (**m**) mice. Mice were generated as described in Methods section, and the protein levels of hepatic and muscular LAMTOR1 were determined by immunoblotting. **n**, **o**, AICAR activates AMPK in muscle or liver tissues with LAMTOR1 knockout. *LAMTOR1*-MKO mice were intraperitoneally injected with 250 mpk AICAR for 2 h, followed by determining the levels of p-AMPK and p-ACC in muscle (**n**) and liver (**o**) tissues by immunoblotting. Experiments in this figure were performed three times. Source data

We next analysed the effect of aldometanib on glycolysis. As shown in Extended Data Fig. 3j,k, the overall glycolytic activity, as determined through monitoring glycolytic rates using labelled glucose (using the levels of labelled FBP as an indicator of glycolytic rate^63,64^) and lactate production, was not inhibited, and was even slightly enhanced in aldometanib-treated cells. The slight increase of glycolysis is likely due to the pro-glycolytic effects of AMPK^65^ activated by the drug (Extended Data Fig. 3l). Consistently, no decrease of cellular energy state, that is, elevation of ratios of AMP:ATP or ADP:ATP, was observed in these cells, unless a 200-nM or higher concentration of aldometanib was used (Fig. 2a–c and Extended Data Fig. 3m). The observation that aldometanib, as an inhibitor of aldolase, does not slow down glycolysis promoted us to wonder if this drug may be enriched in the lysosome. Indeed, we also observed that aldometanib was enriched in the lysosomal fraction compared to other fractions (that is, mitochondrial fraction, ER fraction and cytosolic fraction): according to MS data (Fig. 2d), aldometanib was only detected in lysosomal fractions. We therefore reasoned that the lysosomally localized aldolase was the ‘receptor’ for aldometanib inside cells. It was reported that the protein number (count) of ALDOA was 7,969,332.75 per cell^66^, which is, 1.3238 × 10^−^^17^ mol. The weight of ALDOA (45.12 kDa, or, 45.120 g mol^−^^1^) per cell was 5.973 × 10^−^^1^^3^ g, and the relative content of ALDOA was 2.715 × 10^−^^4^ g/g total cell protein (average cell volume of 2,000 μm^3^, as described previously^9^; cell density of 1.1 g ml^−1^; ref. ^67^). As for the lysosomal ALDOA, 5.135 × 10^−^^5^ g/g total lysosomal protein was reported previously^68^. Given the yield of lysosomes was approximately 0.65% (of total protein) after fractionation according to the results of our study and others^15,69^, the lysosomal fraction of ALDOA was 1/813.422 (that is, 5.135 × 10^−^^5^ × 0.65%/2.715 × 10^−^^4^) of total cell ALDOA. We also observed similar distributions of ALDOB and ALDOC, compared with ALDOA, in lysosomal fractions (Fig. 2e). Together, we estimated an 800-fold enrichment of aldometanib in the lysosome for the lysosomal aldolase to activate AMPK. This may explain why aldometanib at 5 nM is sufficient to activate AMPK inside cells (Fig. 1f,g and Extended Data Fig. 3a,b) and yet the IC_50_ for aldolase is 50 μM in in vitro enzymatic assays (Fig. 1b and Extended Data Fig. 1d). The lysosomal accumulation of aldometanib may be at least in part attributed to the local pH of the lysosome, as the ammonium chloride-treated cells in which lysosomes were deacidified while v-ATPase was left uninhibited^15,70^, showed lowered lysosomal aldometanib concentrations and impaired AMPK activation (Fig. 2f,g). In comparison, endocytic pathways are not involved in the enrichment of aldometanib (Fig. 2h) and aldometanib did not affect endocytosis (Fig. 2i and Extended Data Fig. 3n). Consistently, we found that the IC_50_ of aldometanib for lysosome-bound aldolase (in purified lysosomes) was approximately 15 nM (Fig. 2j), much lower (approximately 333 fold) than 50 μM for in vitro enzymatic assays using membrane-free aldolase (bacterially expressed and purified, or precipitated by ammonium sulfate), indicating that some as yet unclear conditions on the lysosomal membrane inside the live cell may somehow enhance the potency of aldometanib towards aldolase, and cause the enrichment of aldometanib. Moreover, subcellular fractionation assays showed that in cells treated with aldometanib, only the lysosomal aldolase was inhibited (Fig. 2k). We also found that microinjected FBP failed to restore the fluorescence signal of TRPV4-GCaMP6s in the aldometanib-pretreated cells, while FBP restored the fluorescence signal in control cells (Fig. 2l). We next determined the effects of aldometanib in AMPK activation in two rodent models, the C57BL/6 mouse and the ZDF rat. Titrating different doses, we found that administration of aldometanib at 2 mg per kg body weight (mpk; orally gavaged) sufficiently activated AMPK in the rodent liver, skeletal muscle, adipose tissue, heart and kidney, but not brain (Fig. 2m,n and Extended Data Fig. 4b–k). The accumulation of aldometanib in the rodent serum was estimated to be of a few nanomolars, concentrations similar to those used in cell-based experiments (Extended Data Fig. 4a). Similarly, no elevation of AMP:ATP or ADP:ATP ratios was observed in these tissues (Extended Data Fig. 4b–k). In addition, in mice with hepatic or muscular knockout of *Lamtor1*, the activation of AMPK by aldometanib was abrogated in the respective tissues (Fig. 2o,p; see validation data in Extended Data Fig. 4l,m). As an additional control, AICAR (an AMP mimetic) could still activate AMPK in muscle or liver tissues with LAMTOR1 knockout (Extended Data Fig. 4n,o).

**Fig. 3:**
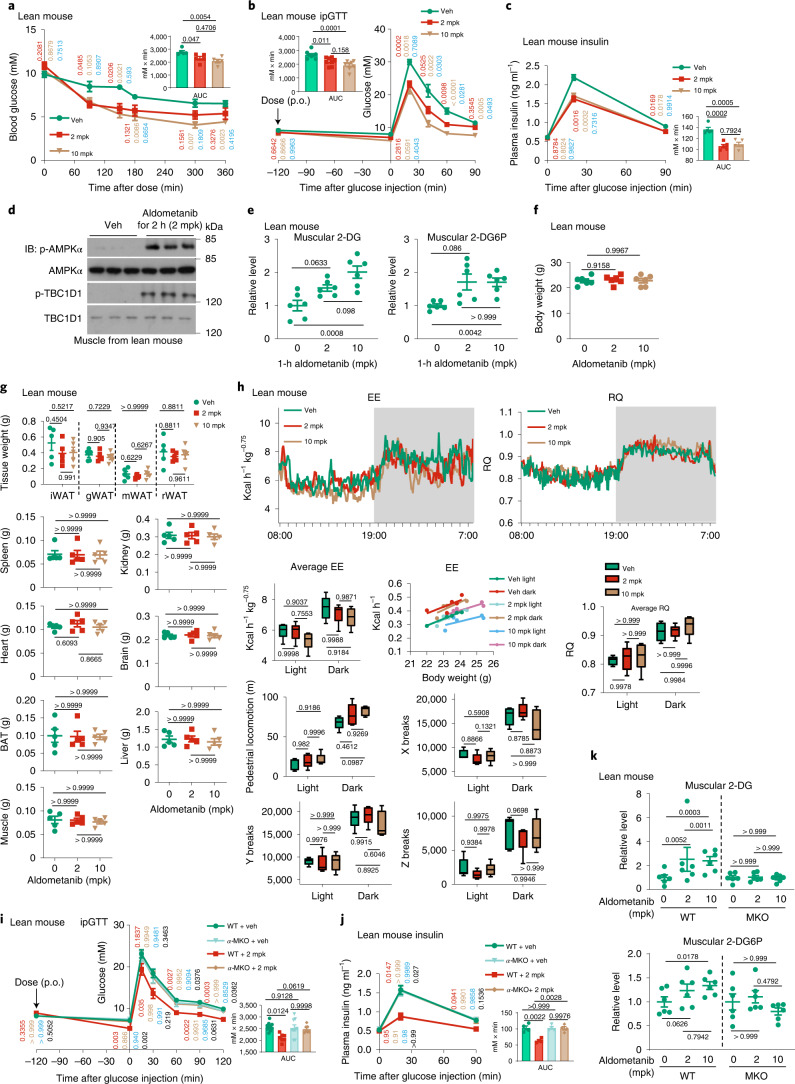
Aldometanib lowers blood glucose in lean mice. **a**–**c**,**i**,**j**, Acute aldometanib administration decreased fasting blood glucose and improved glucose tolerance in normoglycaemic mice. Wild-type and muscle-specific *Ampkα* knockout (*α*-MKO) mice were fasted for 2 h (**a**) or 6 h (**b**, **c**, **i** and **j**), followed by oral gavaging (p.o.) with aldometanib. Blood glucose levels in fasted mice or in mice that underwent an intraperitoneal glucose tolerance test (ipGTT) are shown as the mean ± s.e.m.; *n* = 5 (**a** and **c**), 8 (**b**), 10 (**i**; vehicle and WT), 9 (**i**; vehicle and *α*-MKO), 7 (**i**; 2 mpk) or 4 (**j**) mice for each treatment; *P* values were determined by two-way (blood glucose and insulin values), one-way (area under the curve (AUC) values of **a**–**c**) or two-way (AUC values of **i** and **j**) repeated-measures (RM) ANOVA followed by Tukey’s test, and are indicated on the curves: for **a**–**c**, comparisons between the control (Veh) and the 2-mpk group are coloured in red, control and 10-mpk group in beige, and 2-mpk and 10-mpk groups in blue; for **i** and **j**, WT + Veh and WT + 2 mpk are coloured in red, *α*-MKO + Veh and *α*-MKO + 2 mpk in beige, WT + Veh and *α*-MKO + Veh in blue, and WT + 2 mpk and *α*-MKO + 2 mpk in black. **d**,**e**,**k**, Aldometanib promoted muscular TBC1D1 phosphorylation and glucose uptake. Wild-type or *α*-MKO mice were starved for 5 h, followed by gavaging with aldometanib. After 1 h (**e** and **k**) or 2 h (**d**) of aldometanib gavaging, mice were killed and muscle tissue was collected for immunoblotting (IB) (**d**), or mice were intraperitoneally injected with 1.25 mpk glucose supplemented with 0.125 mpk 2-DG, followed by determining the muscular levels of 2-DG and 2-DG6P 2 h later (**e** and **k**). Results in **e** and **k** are shown as the mean ± s.e.m.; *n* = 6 mice for each treatment, and *P* values were determined by one-way ANOVA followed by Dunnet’s test (levels of 2-DG6P of **e**, and levels of 2-DG of **k**), Dunn’s test (levels of 2-DG6P of *α*-MKO of **k**) or Tukey’s test (others). **f**–**h**, Acute aldometanib administration does not change body weight or EE. Mice were treated as in **b**, followed by determining body weight (**f**), body composition (**g**) and EE (**h**; see also respiratory quotient (RQ) and ambulatory activity data). Data are shown as the mean ± s.e.m. (**f** and **g**), mean (left part of **h**, at 5-min intervals during a 24-h course after normalization to the body weight (kg^0.75^)), or as box-and-whisker plot (right part of **h**, in which the lower and upper bounds of the box represent the first and the third quartile scores, the centre line represents the median, and the lower and upper limits denotes minimum and maximum scores, respectively; and the same hereafter for all box-and-whisker plots); *n* = 6 (**f**) or 5 (**g** and **h**) mice for each treatment; and *P* values were determined by one-way (**f** and **g**) or two-way (**h**) ANOVA, followed by Dunn’s (heart, kidney, liver, muscle, spleen, BAT and brain of **g**) or Tukey’s (others) test. gWAT, gonadal white adipose tissue; mWAT, mesenteric adipose tissue; rWAT, perirenal WAT; BAT, brown adipose tissue. Experiments were performed three times, except in **a** (four times). Source data

**Fig. 4:**
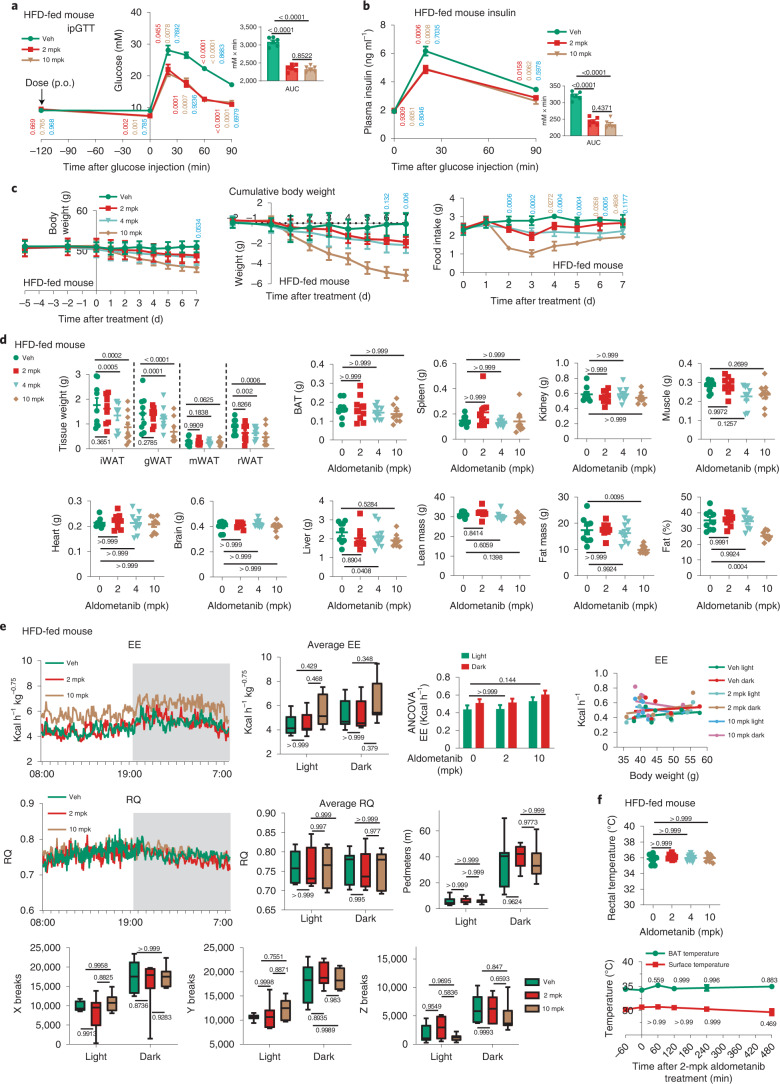
Aldometanib lowers blood glucose in obese hyperglycaemic mice. **a**,**b**, Treatment with aldometanib for 1 week decreased blood glucose in HFD-induced obese mice. Experiments were performed as in Fig. 3b, except that mice fed with a HFD for 16 weeks were used, and mice were gavaged with aldometanib twice daily for a week. Results are shown as the mean ± s.e.m.; *n* = 6 mice for each treatment; *P* values were determined by two-way RM (blood glucose and insulin values) or one-way (AUC values) ANOVA followed by Tukey’s test, and are labelled as in Fig. 3a. **c**–**f**, The 1-week treatment of aldometanib did not change body composition and energy metabolism in HFD-induced obese mice at effective doses. Mice were treated with 2 mpk, 4 mpk (effective doses) or 10 mpk (high dose) aldometanib for 1 week, followed by determination of body weight (**c**); body composition (**d**); EE, RQ and ambulatory activity (**e**); and rectal (**f**; upper) and surface (**f**, lower panel) temperatures. Data are shown as the mean ± s.e.m., except **e** as in Fig. 3h (for analysis of covariance (ANCOVA), the normalized weight was 45.2 g); *n* = 9 (**c**, **d** and **f**) or 7 (**e**) mice for each treatment; *P* values were determined by two-way RM ANOVA, followed by Tukey’s test (**c**; *P* values are indicated as in Fig. 3a) or Sidak’s test (lower part of **f**), by two-way ANOVA followed by Tukey’s test (**e**; except ANCOVA analysis), or by one-way ANOVA followed by Dunn’s test (BAT, spleen, kidney, heart and brain of **d**; and upper part of **f**), Dunnet’s test (fat mass of **f**) or Tukey’s test (others). Experiments were performed three times. Source data

**Fig. 5:**
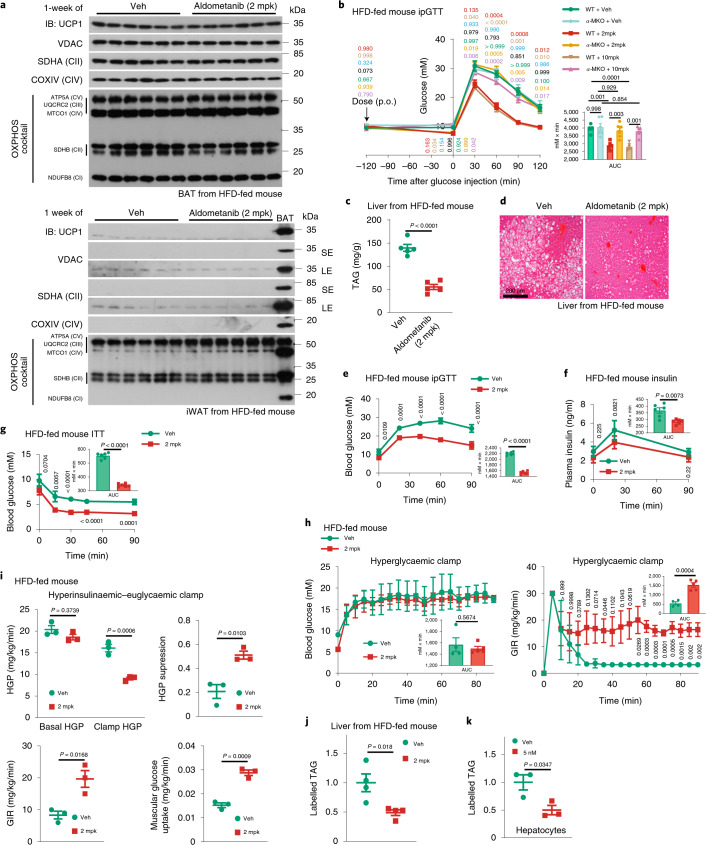
Aldometanib alleviates fatty liver. **a**, The 1-week treatment of aldometanib did not induce browning of adipose tissues in HFD-induced obese mice. LE, long exposure; SE, short exposure. **b**, The 1-week treatment of aldometanib lowered blood glucose in a muscular AMPK-dependent manner in HFD-induced obese mice. Experiments were performed as in Fig. 4a. Results are shown as the mean ± s.e.m.; *n* = 6 mice for each treatment; *P* values were determined by two-way RM (values of blood glucose) or two-way (values of AUC) ANOVA followed by Tukey’s test, and are indicated on the curves in colours: comparisons between the WT + Veh and WT + 2 mpk are coloured in red, WT + Veh and WT + 10 mpk in beige, *α*-MKO + Veh and *α*-MKO + 2 mpk in blue, *α*-MKO + Veh and *α*-MKO + 10 mpk in black, WT + Veh and *α*-MKO + Veh in green, WT + 2 mpk and *α*-MKO + 2 mpk in yellow, and WT + 10 mpk and *α*-MKO + 10 mpk in purple. **c**,**d**, The 1-month treatment of aldometanib reduced hepatic TAG in diet-induced obese mice. Mice were treated as in Fig. 4a, except that mice were gavaged with aldometanib twice daily for a month. Hepatic TAG levels (**c**; results are shown as the the mean ± s.e.m.; *n* = 5 mice for each treatment, and *P* values were determined by two-sided Student’s *t*-test) and representative images from H&E staining of the liver are shown (**d**). **e**–**g**, The 1-month treatment of aldometanib improved insulin sensitivity in obese mice. Mice were treated as in **c**, followed by ipGTT (**e**; see also serum insulin levels during ipGTT in **f**) or ITT (**g**). Data are shown as the mean ± s.d., *n* = 6 mice for each treatment, and *P* values were were determined by two-way RM ANOVA followed by Sidak’s test (glucose and insulin), by two-sided Student’s *t*-test (AUC values of **e** and **g**), or by two-sided Student’s *t*-test with Welch’s correction (AUC values of **f**). **h**,**i**, The 1-month treatment of aldometanib increased glucose disposal rates in obese mice. Mice were treated as in **c**, followed by performing a hyperglycaemic (**h**) or hyperinsulinaemic–euglycaemic (**i**) clamp. Mice were treated as in **c**, followed by determining the glucose infusion rate (GIR; right part of **h**) when plasma glucose concentrations were maintained at 16–18 mM (left in **h**), or the HGP (upper left of **i**; see also the percentage of HGP suppressed by insulin infused during the clamp in upper right of **i**) and the muscular glucose uptake rate (lower right of **i**) by hyperinsulinaemic–euglycaemic clamp; see also GIR values during the clamp in lower left of **i**). Data are the mean ± s.d. (**h**) or s.e.m. (**i**); *n* = 4 (**h**; veh), 5 (**h**; aldometanib-treated) or 3 (**i**) mice for each treatment, and *P* values were determined by RM two-way ANOVA followed by Sidak’s test (GIR), by two-way ANOVA followed by Tukey’s test (**i**; HGP), or by two-sided Student’s *t*-test (**h**, AUC; **i**, others) **j**,**k**, Aldometanib inhibits TAG synthesis in liver and primary hepatocytes. Rates of TAG synthesis were assessed by labelled TAG determined either from liver tissues excised from mice gavaged with aldometanib and infused with uniformly labelled ^13^C [U-^13^C]-glucose through the jugular vein (**j**), or from primary hepatocytes incubated with [U-^13^C]-glucose (**k**). Data are the mean ± s.e.m.; *n* = 4 mice (**j**) or 3 dishes of cells (**k**) for each treatment; *P* values were determined by two-sided Student’s *t*-test. Experiments were performed three times. Source data

**Extended Data Fig. 5:**
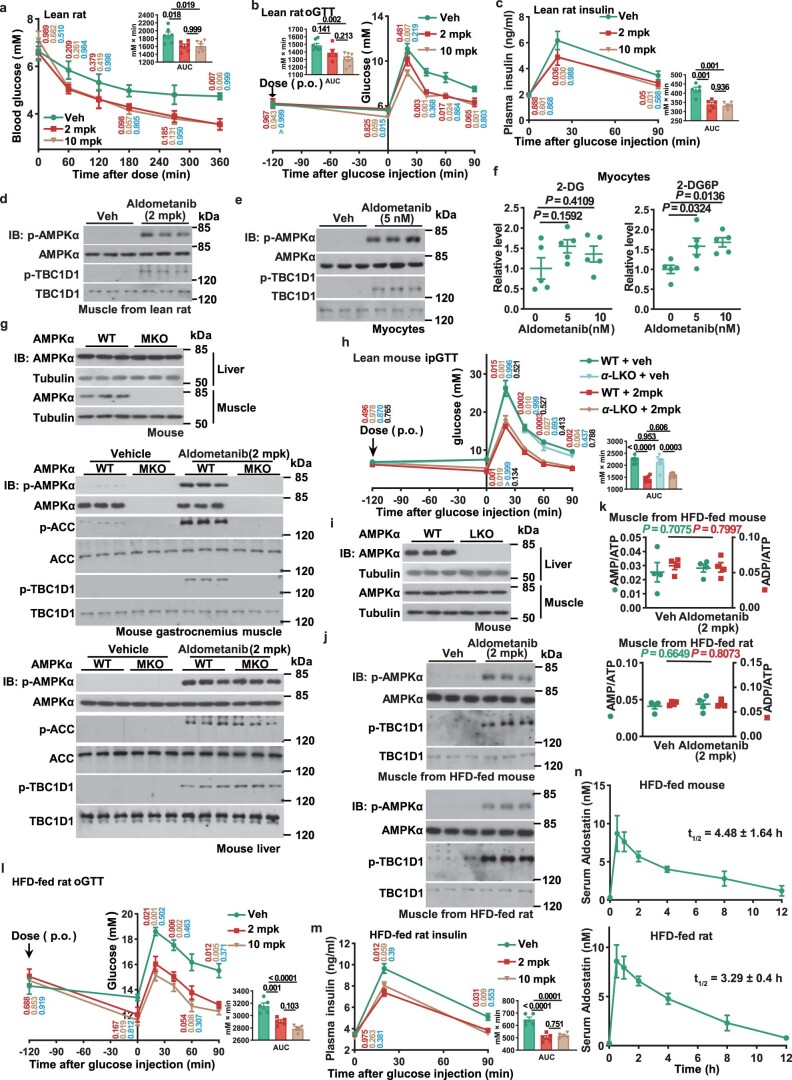
Aldometanib increases muscular glucose uptake to lower blood glucose. **a**-**c**, Acute aldometanib administration lowers blood glucose in normoglyceamic rats. Rats were fasted for 6 h, followed by gavaging of 2 mpk or 10 mpk aldometanib. Blood glucose levels in fasted rats or in rats undergo oral glucose tolerance test (oGTT) were measured and shown as mean ± s.e.m., *n* = 6 (**a**), 7 (vehicle of **b**), 5 (2 mpk of **b**; **c**) or 8 (10 mpk of **b**) rats for each treatment; *P* value by two-way RM (glucose) or one-way ANOVA (AUC) ANOVA, followed by Tukey (labelled as in Fig. 3a). **d**-**f**, **j**, aldometanib promotes glucose uptake. Levels of p-TBC1D1 in rat muscle (**d**), myocytes (**e**, treated as in Extended Data Fig. 3c) and HFD-induced obese mouse (**j**, upper) or rat (**j**, lower) muscle, and levels of 2-DG and 2-DG6P in myocytes (**f**) were determined. In **d**, **j**, rats were gavaged with 2 mpk aldometanib, and tissues were excised 2 h after gavaging. In **f**, primary myocytes were treated with 5 nM or 10 nM aldometanib for 2 h, followed by incubating with 0.1 mM 2-DG for another 10 min. Results of **f** are mean ± s.e.m.; *n* = 5 dishes of cells for each treatment, and *P* value by one-way ANOVA followed by Dunn (left panel) or Tukey (right panel). **g**, **i**, Validation of knockout efficiency and specificity of knockout in *α*-MKO (**g**) and *α*-LKO (**i**) mice. Tissue samples were collected (in the middle and lower panels of **g**, mice were treated as in **d**), followed by immunoblotting. **h**, aldometanib can lower blood glucose without hepatic AMPK. Experiments were performed as in Fig. 3i, except that liver-specific *AMPKα* knockout (LKO) mice were used. Results are shown as mean ± s.e.m.; *n* = 6 (WT, and LKO + aldometanib) or 7 (others) mice, and *P* values by two-way RM (glucose) or two-way (AUC) ANOVA, followed by Tukey (labelled as in Fig. 3i). **k**, aldometanib does not elevate AMP in obese rodent muscle. HFD-induced obese mice (upper panel) or rats (lower panel) were gavaged with aldometanib. At 2 h post-gavaging, muscular AMP:ATP and ADP:ATP ratios were determined. Results are mean ± s.e.m.; *n* = 4 mice/rats for each treatment, and *P* value by two-sided Student’s *t*-test. **l**, **m**, The 1-week treatment of aldometanib decreases blood glucose in HFD-induced obese rats. Experiments were performed as in **b**, except that rats fed with HFD for 12 weeks were used, and that aldometanib was gavaged twice a day for a week. Results are mean ± s.e.m.; *n* = 5 rats for each treatment, and *P* values by two-way RM (glucose) or one-way (AUC) ANOVA followed by Tukey (labelled as in Fig. 3a). **n**, Serum aldometanib concentrations in obese mice and rats. Experiment were performed as in Extended Data Fig. 4a; data are mean ± s.d.; *n* = 5 mice/rats. See also half-life (t_1/2_) of aldometanib on each panel. Experiments in this figure were performed three times. Source data

We next analysed the effects of aldometanib in glucose homeostasis. We found that acute aldometanib administration (single-dose, orally gavaged) at 2 mpk significantly decreased fasting blood glucose in regularly fed, normoglycaemic mice and rats (Fig. 3a and Extended Data Fig. 5a). Concomitantly, aldometanib improved glucose tolerance during glucose challenge (glucose tolerance test (GTT); Fig. 3b and Extended Data Fig. 5b). Such improved glucose homeostasis was accompanied by significantly lower plasma insulin levels compared to those in control mice (Fig. 3c and Extended Data Fig. 5c), implying a direct promotion of glucose absorption (rather than through stimulating insulin secretion). Indeed, phosphorylation of TBC1D1 was promoted in muscle tissues after aldometanib treatment (Fig. 3d and Extended Data Fig. 5d), and muscle uptake of 2-deoxy-d-glucose (2-DG), a non-fermentable glucose analogue, was also significantly enhanced, as indicated by the increased formation of its metabolite, 2-deoxy-d-glucose-6-phosphate (2-DG6P; Fig. 3e). Such effects were also observed in mouse primary muscle cells in vitro (Extended Data Fig. 5e,f). No changes in body weight, body composition and energy expenditure (EE) could be observed in mice acutely treated with aldometanib (Fig. 3g,h). Muscle-specific knockout of AMPK (both *AMPKα1* and *AMPKα2*) in mice abrogated the effects of aldometanib in glucose lowering (Fig. 3i–k; see validation data in Extended Data Fig. 5g), consistent with previous reports showing a prominent role of muscular AMPK in lowering blood glucose^42,71,72^. As an additional control, mice with liver-specific knockout of AMPK exhibited intact glucose-lowering effects of aldometanib (Extended Data Fig. 5h; see validation data in Extended Data Fig. 5i). We also tested for the antihyperglycaemic efficacy of aldometanib in high-fat diet (HFD)-induced obese mice and rats. Twice-daily administration of aldometanib for a week showed a continued promotion of TBC1D1 phosphorylation in skeletal muscle, without elevating AMP levels (Extended Data Fig. 5j,k), and significantly improved glucose tolerance as determined by GTT (Fig. 4a and Extended Data Fig. 5l), along with lowered serum insulin levels compared to those in control mice after glucose challenge (Fig. 4b and Extended Data Fig. 5m). Similarly to those observed in lean mice acutely treated with aldometanib, no changes in body weight (unless a higher dose, that is, 10 mpk was administered), body composition and EE could be detected in obese mice treated with aldometanib for 1 week (Figs. 4c–f and 5a). We also observed that, in obese mice with muscular knockout of AMPK, the effects of aldometanib in glucose lowering were abolished (Fig. 5b). Notably, the plasma concentrations of aldometanib in these rodents were comparable to those detected in lean animals (Extended Data Fig. 5n).

### Aldometanib alleviates fatty liver and nonalcoholic steatohepatitis

**Fig. 6:**
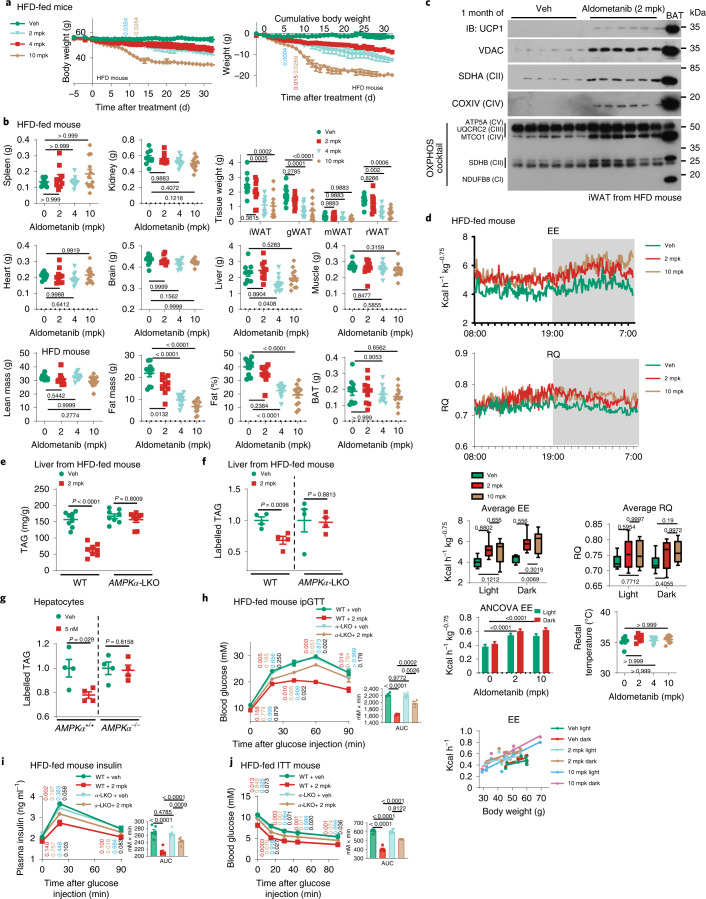
Aldometanib reduces fat mass. **a**–**d**, The 1-month treatment of aldometanib decreased fat mass, induced browning and elevated EE in HFD-induced obese mice. Mice were gavaged with aldometanib twice daily for a month, followed by determination of body weight (**a**), body composition (**b**), the mitochondrial contents of iWAT (**c**) and EE (**d**; *n* = 8 mice, shown and analysed as in Fig. 3h; and the normalized mouse weight for ANCOVA was 48.3604 g). Data in **a** and **b** are the mean ± s.e.m.; *n* = 10 (Veh and 2 mpk), 11 (4 mpk) or 12 (10 mpk) mice for each treatment; and *P* values were determined by two-way RM ANOVA, followed by Tukey’s test (**a**; labelled as in Fig. 4c), or by one-way ANOVA, followed by Dunn’s test (**b**; lean mass, BAT, spleen, kidney, brain and muscle) or Tukey’s test (**b**; others). See also rectal temperature measured in mice after 1 h of aldometanib gavage (**d**; mean ± s.e.m.; *n* = 7 mice for each treatment; *P* values were determined by one-way ANOVA, followed by Dunn’ test). **e**–**j**, Knockout of AMPK prevented aldometanib from alleviating fatty liver. Levels of hepatic TAG (**e**), rates of TAG synthesis in liver (**f**) and primary hepatocytes (**g**), glucose tolerance (**h**, ipGTT; see also serum insulin levels during GTT in **i**) and insulin sensitivity (**j**, ITT) are shown. Data were generated either from HFD-induced obese *α*-LKO mice (**e**, **f** and **g**–**j**; HFD-fed mice were treated with aldometanib as in Fig. 5c,e–g,j, except that the mice were injected with tamoxifen, three times a week for 1 week, to deplete hepatic AMPK before the aldometanib treatment), or from *AMPKα*^−/−^ primary hepatocytes (**g**; treated as in Fig. 5k, except that cells were isolated from *α*-LKO mice). Data are the mean ± s.e.m. from *n* = 8 (**e**), 4 (**f** and **h**) or 6 (**i** and **j**) mice, or four dishes of cells (**g**); *P* values were determined by two-way ANOVA, followed by Tukey’s test (**e**; AUC values of **h**, **i** and **j**), two-sided Student’s *t*-test (**f** and **g**), or two-way RM ANOVA, followed by Tukey’s test (glucose values of **h**–**j**; labelled as in Fig. 3i). Experiments were performed three times. Source data

**Extended Data Fig. 6:**
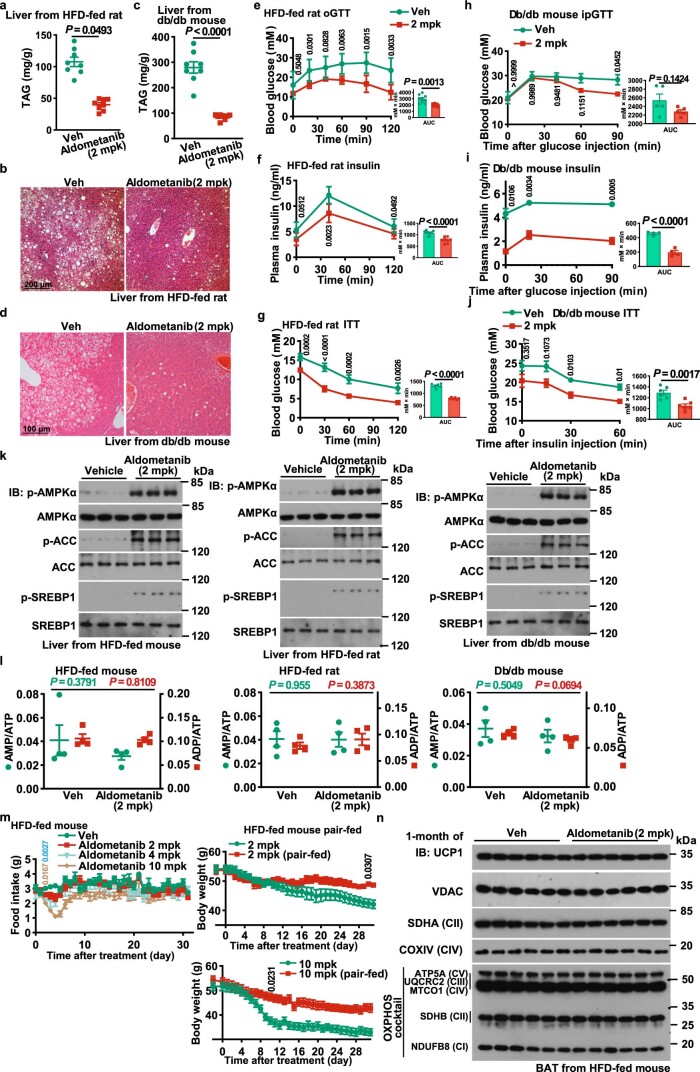
Aldometanib alleviates fatty liver. **a**-**d**, The 1-month treatment of aldometanib reduces alleviates fatty liver in obese rodents. Rats (**a**, **b**) and Db/db (**c**, **d**) mice were fed with HFD and standard diet, respectively, for 12 weeks. aldometanib was administered twice-daily for a month, followed by determining hepatic TAG levels. In **a**, **c**, data are mean ± s.e.m.; *n* = 8 mice/rats for each treatment, *P* value by two-sided Student’s *t*-test with Welch’s correction. See also representative images from hepatic H&E staining in **b**, **d**. **e**-**j**, The 1-month treatment of aldometanib improves insulin sensitivity in obese rodents. Rats and db/db mice were treated as in **a**, followed by performing oGTT (**e**, for rats; see also insulin levels in **f**), ipGTT (**h**, for db/db mice, insulin levels in **i**) and ITT (**g**, **j**). Data are mean ± s.d. (rats) or s.e.m. (db/db mice), *n* = 9 (**e**), 6 (**f**, **g**, **j**), 5 (**h**), or 4 (**i**) mice/rats for each treatment, and *P* value by two-way RM ANOVA followed by Sidak (glucose), by two-sided Student’s *t*-test (AUC of **f**, **g**, **j**, **i**), or by two-sided Student’s *t*-test with Welch’s correction (AUC of **e**, **h**). **k**, **l**, aldometanib promotes hepatic ACC and SREBP1 phosphorylation in obese rodents without elevating AMP. HFD-induced obese mice (left), HFD-induced obese rats (middle) or db/db mice (right) were gavaged with aldometanib, followed by determining p-ACC, p-SREBP, and adenylate ratios [mean ± s.e.m.; *n* = 4 mice/rats for each treatment, and *P* value by two-sided Student’s *t*-test with Welch’s correction (AMP:ATP of HFD mice) or two-sided Student’s *t*-test (others)] in liver tissues 2 h after gavaging. **m**, **n**, The 1-month treatment of aldometanib does not affect food intake, unless high doses are administered. Mice were treated as in Fig. 6a, and the daily food intake (**m**, left panel) and mitochondrial contents of BAT (**n**) were determined. See also body weight of mice in the pair-feeding experiments on the right panel of **m**. Data are shown as mean ± s.e.m.; *n* = 11 (4 mpk of food intake), 12 (10 mpk of food intake) or 10 (others) mice for each treatment; and *P* values by two-way RM ANOVA, followed by Tukey. Experiments in this figure were performed three times. Source data

**Extended Data Fig. 7:**
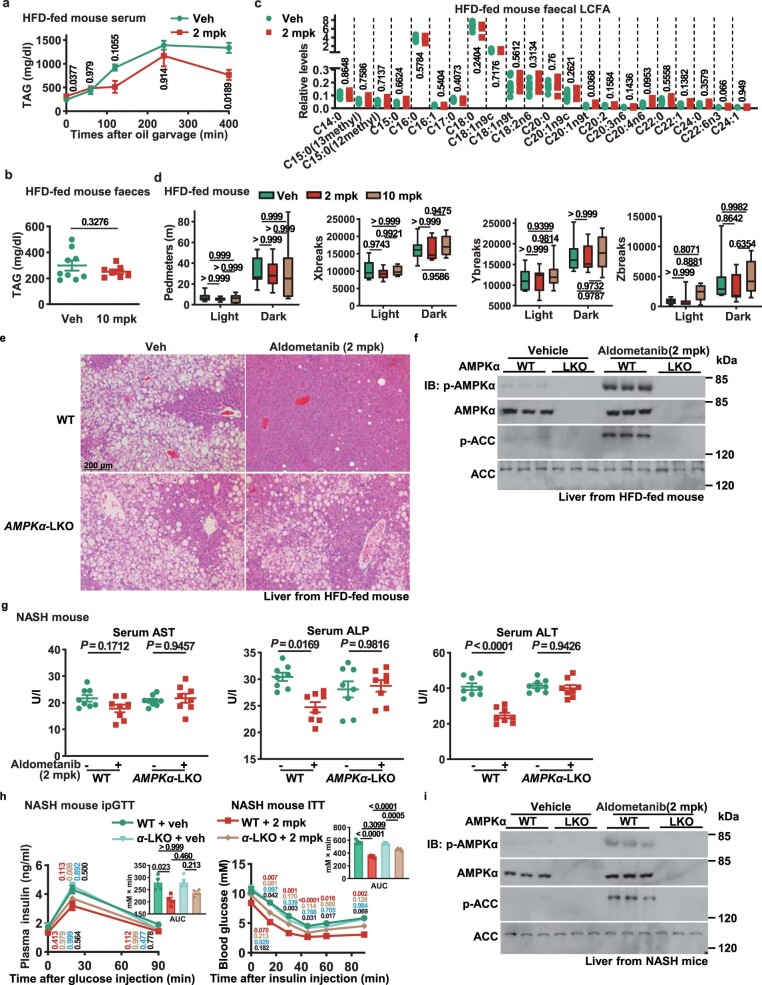
AMPK is required for aldometanib to alleviate fatty liver and NASH. **a**, aldometanib impairs post-prandial TAG absorption. The HFD-induced obese mice were starved for 8 h, followed by injection with Tyloxapol and gavaged with aldometanib. Some 0.5 h later, mice were gavaged with 10 ml/kg olive oil, followed by determining serum TAG. Data are mean ± s.e.m., *n* = 6 mice for each treatment, and *P* value by two-way ANOVA followed by Sidak (TAG). **b**, **c**, aldometanib does not change faecal lipid content. The HFD-induced obese mice were gavaged with aldometanib. At 1 h after gavaging, levels of faecal TAG (**b**) and long-chain fatty acids (**c**) were determined. Data are mean ± s.e.m., *n* = 9 (**b**, Veh), 7 (**b**, 2 mpk; **c**, Veh), and 6 (**c**, 2 mpk) mice for each treatment, and *P* value by two-sided Student’s *t*-test. **d**, The 1-month treatment of aldometanib administration does not change ambulatory activity. Experiments were performed as in Fig. 6d. Data are shown as box-and-whisker plots (*n* = 8 mice, *P* values by two-way ANOVA, followed by Tukey). **e**, Hepatic knockout of AMPK impairs the effects of 1-month aldometanib treatment in reducing hepatic TAG; experiments as in Fig. 5c, but using *AMPKα*-LKO mice. **f**, **i**, aldometanib promotes AMPK activation in the liver of obese and NASH mouse models. HFD-induced obese mice (**f**) and the AMLN-NASH mice (**i**) were gavaged with aldometanib, followed by determining AMPK activation in liver tissues 2 h after gavaging. **g**, **h**, Hepatic knockout of AMPK impairs the effects of aldometanib in alleviating NASH. Mice were treated as in Fig. 7a, followed by measuring serum AST (**g**, left panel), ALP (**g**, middle panel), ALT (**g**, right panel), insulin levels during the ipGTT (**h**, left panel), and performing ITT (**h**, right panel). Data are mean ± s.e.m.; *n* = 8 (**g**), 4 (**h**, left panel) or 5 (**h**, right panel) mice for each genotype/treatment, and *P* value by two-way (**g**, AUC of **h**), two-way RM (glucose of **h**) ANOVA, followed by Tukey (labelled as in Fig. 3i). Experiments in this figure were performed three times. Source data

We next determined the effects of aldometanib in alleviating fatty liver, another beneficial effect of AMPK activation^46,73–76^. We found that twice-daily administration of 2 mpk of aldometanib for a month significantly reduced hepatic triacylglycerol (TAG) levels in *db/db* mice, HFD-induced obese mice and HFD-induced obese rats (Fig. 5c,d and Extended Data Fig. 6a–d). Other physiological effects included improved glucose tolerance and insulin sensitivity (determined by GTT, insulin tolerance test (ITT), hyperglycaemic clamp and hyperinsulinaemic–euglycaemic clamp) and decreased hepatic glucose production (HGP; determined by hyperinsulinaemic–euglycaemic clamp; Fig. 5e–i and Extended Data Fig. 6e–j). Consistently, an increased phosphorylation of ACC and SREBP1 was observed in the livers of these animals (Extended Data Fig. 6k), and no AMP elevation was detected (Extended Data Fig. 6l). We also determined the de novo lipogenesis rates by monitoring the incorporation of ^13^C-glucose to hepatic TAG, and found that aldometanib significantly inhibited lipogenesis rates (Fig. 5j,k). We also observed a significant reduction of body weight, fat mass and body fat composition in mice treated with aldometanib for 1 month, although the lean mass was unchanged (Fig. 6a,b). Such reduced fat mass may be attributed to: (a) the lowered food intake, especially during the first 10 d of high-dose treatment (Extended Data Fig. 6m); (b) the browning of inguinal white adipose tissue (iWAT), as evidenced by the increased protein levels of mitochondrial complexes in iWAT (Fig. 6c and Extended Data Fig. 6n); (c) increased average EE (for both light and dark periods; Fig. 6d and Extended Data Fig. 7d), possibly due to the browning of iWAT; and (d) the decreased intestinal fat absorption, particularly at the postprandial state, as assessed by the oral fat tolerance test (Extended Data Fig. 7a–c). Liver-specific knockout of AMPK in mice blocked the effects of aldometanib in lowering hepatic TAG levels and hepatic de novo lipogenesis rates (Fig. 6e–g and Extended Data Fig. 7e,f), indicating that hepatic AMPK is required for the effects of aldometanib in alleviating fatty liver. Impaired glucose tolerance was also observed in the aldometanib-treated mice with hepatic knockout of AMPK (Fig. 6h–j). However, these mice still showed improved glucose tolerance compared with control mice (without aldometanib treatment; Fig. 6h–j), possibly due to the decrease of fat content, which, in addition to the fatty liver alleviation, is known to improve insulin sensitivity and hence improve glucose tolerance^77^.

**Fig. 7:**
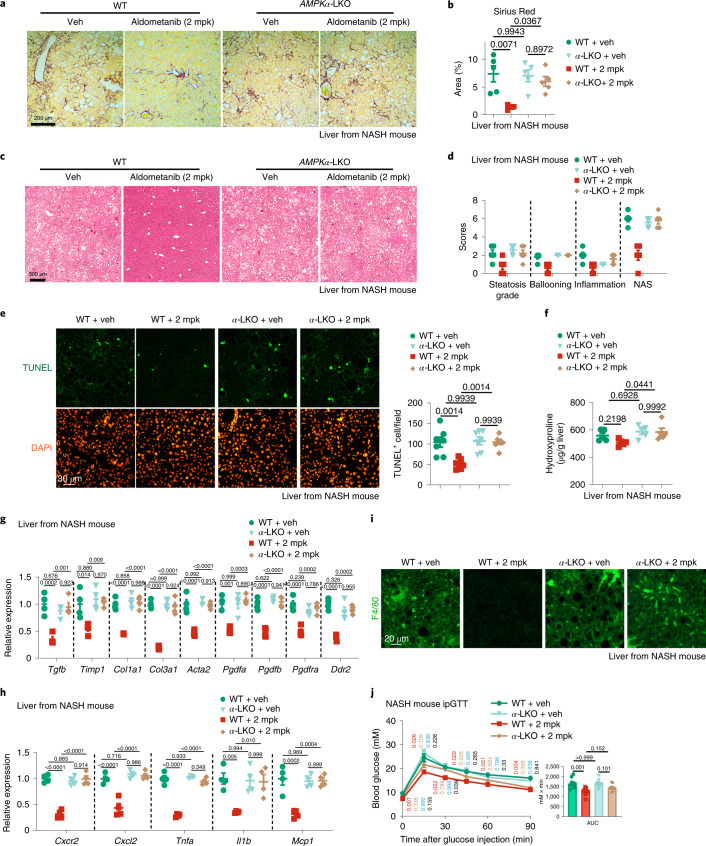
Aldometanib alleviates NASH. **a**,**b**,**f**,**g**, Aldometanib alleviated liver fibrosis in NASH mice. Mice were fed with AMLN diet for 30 weeks, and then twice-daily administration of 2 mpk aldometanib for a month. Hepatic AMPK was depleted by injecting tamoxifen 3 times a week for 1 week before the aldometanib treatment. Representative images of hepatic Sirius Red staining (**a**) and statistical analysis (**b**), hepatic levels of hydroxyproline (**f**) and the mRNA levels of fibrogenic genes (**g**) are shown. Data are the mean ± s.e.m.; *n* = 4 (**g**) or 5 (others) mice for each genotype/treatment; *P* values were determined by two-way ANOVA, followed by Tukey’s test. **c**,**d**, Aldometanib treatment decreased histological scores used to describe the features of NASH. Mice were treated as in **a**. Representative images from H&E staining of the liver (**c**), the NASs, ballooning scores, steatosis grades and the lobular inflammation scores (**d**; mean ± s.e.m. from *n* = 5 mice; *P* values were determined by two-way ANOVA, followed by Tukey’s test) are shown. **e**, Aldometanib reduces apoptosis rate of hepatic cells in NASH mice. Mice were treated as in **a**. Representative images from TUNEL/BrdU staining of the liver are shown, together with statistical analysis. Data are the mean ± s.e.m.; *n* = 7 mice for each genotype/treatment; *P* values were determined by two-way ANOVA, followed by Tukey’s test. **h**,**i**, Aldometanib inhibits inflammatory responses in the liver of NASH mice. Mice were treated as in **a**, and the mRNA levels of pro-inflammatory genes (**h**) are shown as the mean ± s.e.m. (*n* = 4 mice for each genotype/treatment). *P* values were determined by two-way ANOVA, followed by Tukey’s test. Representative images from F4/80 staining of the liver are shown (**i**). **j**, Aldometanib improved glucose tolerance of NASH mice. Mice were treated as in **a**, and results of ipGTT are shown as the mean ± s.e.m. (*n* = 9 mice for each genotype/treatment). *P* values were determined by two-way RM (glucose) or two-way (AUC) ANOVA, followed by Tukey’s test. Experiments were performed three times. Source data

We also determined whether aldometanib could alleviate nonalcoholic steatohepatitis (NASH), which is a progressive form of fatty liver that may develop liver injury and can lead to cirrhosis and hepatocellular carcinoma^46,48,78,79^. We found that twice-daily aldometanib (2 mpk) administration for 1 month significantly alleviated hepatic fibrosis, as determined by Sirius Red staining, and in mice fed with the amylin liver NASH (AMLN) diet for 30 weeks (Fig. 7a and quantified in Fig. 7b), which is a mouse model for human NASH in preclinical studies^80^. Consistently, the nonalcoholic fatty liver disease (NAFLD) activity scores (NASs), ballooning scores, steatosis grades and the lobular inflammation scores used to describe the histological features of NASH^81,82^, were all reduced in the liver from mice treated with aldometanib (Fig. 7c,d). The apoptosis rate of hepatic cells (assessed by TUNEL/BrdU staining; Fig. 7e), levels of liver fibrosis (determined by the levels of hepatic hydroxyproline, Fig. 7f; and the mRNA levels of fibrogenic genes, Fig. 7g) and the inflammatory responses in the liver (determined by the mRNA levels of pro-inflammatory genes, Fig. 7h; and the infiltration of macrophages, Fig. 7i) were all significantly inhibited in aldometanib-treated NASH mice. We also observed significantly decreased serum alanine aminotransferase (ALT) and alkaline phosphatase (ALP) activities, markers for liver injury, in aldometanib-treated NASH mice (Extended Data Fig. 7g). Similarly, glucose tolerance was significantly improved in these mice (Fig. 7j and Extended Data Fig. 7h). Liver-specific knockout of AMPK impaired all the effects of aldometanib in alleviating NASH in mice (Fig. 7 and Extended Data Fig. 7g–i).

### Aldometanib extends lifespan and healthspan

**Fig. 8:**
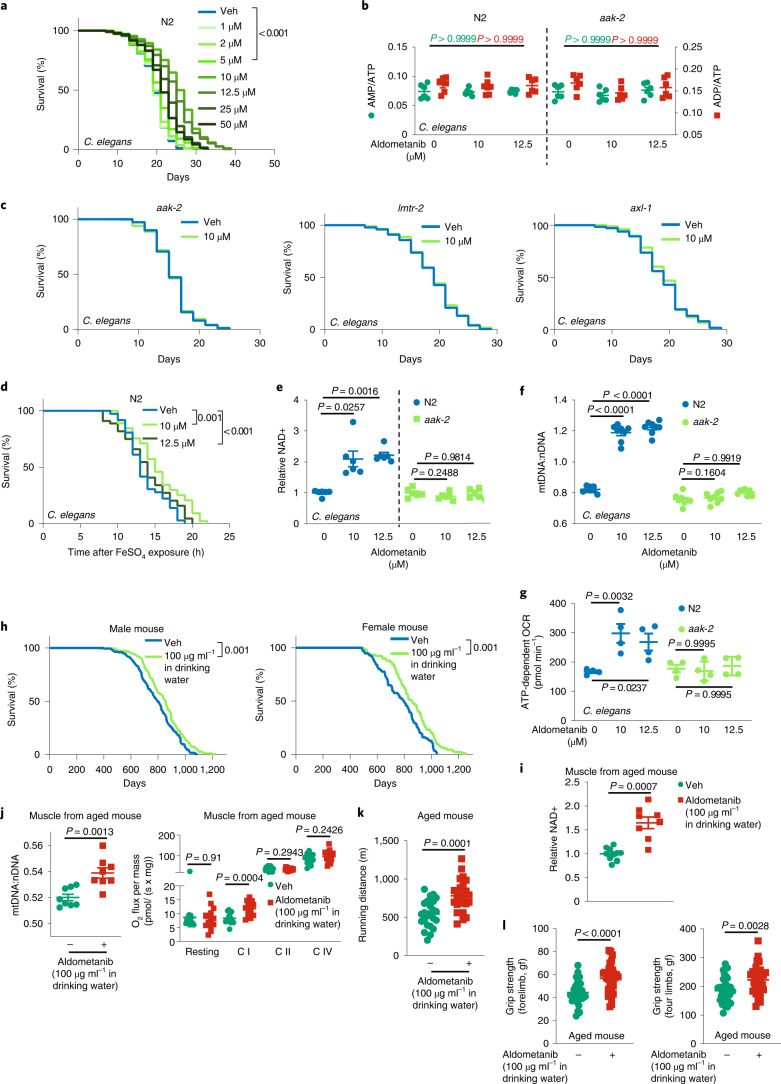
Aldometanib extends lifespan and healthspan. **a**–**c**,**e**, Aldometanib extended lifespan in *C*. *elegans* via the lysosomal pathway. Wild-type (N2) nematodes or nematodes with *AMPKα* (*aak*-*2*), *LAMTOR2* (*lmtr*-*2*) or *AXIN* (*axl*-*1*) knocked out were cultured on the agar plates containing aldometanib at indicated concentrations. Lifespan data are shown as Kaplan–Meier curves (**a** and **c**; see also statistical analyses on Supplementary Table 2, and the same hereafter for all lifespan data), and AMP:ATP and ADP:ATP ratios (**b**) and NAD^+^ (**e**) levels after 2-d treatment of aldometanib are shown as the mean ± s.e.m. (*n* = 6 dishes of worms for each treatment, and *P* values were determined by one-way ANOVA followed by Dunn’s (**b**; N2 of **e**) or Dunnet’s (others) test). **d**, Aldometanib promoted oxidative stress resistance in *C*. *elegans*. N2 nematodes were cultured on the agar plates containing aldometanib for 1 d, followed by treatment with 15 mM ferrous sulfate. **f**,**g**, Aldometanib promoted mitochondrial functions in *C*. *elegans*. The N2 and *aak*-*2* nematodes were cultured on the agar plates containing aldometanib for 1 d, followed by determining the mtDNA:nDNA (**f**) and OCRs (**g**). Data are shown as the mean ± s.e.m.; *n* = 8 (**f**) or 4 (**g**) dishes of worms for each treatment, and *P* values were determined by two-way ANOVA followed by Tukey’s test. **h**, Aldometanib extended lifespan in mice. Male or female C57BL/6 mice at 52 weeks of age were treated with aldometanib in drinking water. **i**,**j**, Aldometanib elevated NAD^+^ levels and mitochondrial oxidative respiration in aged mouse muscle. Mice were treated as in **h**, except for 4 months, followed by determination of levels of muscular NAD^+^ (**i**), the activities of muscular mitochondrial complex I to IV (**j**) and the levels of muscular mtDNA:nDNA (**j**). Data are the mean ± s.e.m., *n* = 10 (control of **i**), 8 (aldometanib group of **i**, and right part of **j**) or 14 (left part of **j**) mice, and *P* values were determined by two-sided Student’s *t*-test with Welch’s correction (**i**), two-tailed Mann–Whitney test (resting group of right part of **j**) or two-sided Student’s *t*-test (others). **k**,**l**, Aldometanib rejuvenates muscle function in aged mice. Mice were treated as in **i**, followed by determination of running distance (**k**) and grip strength (both the forelimbs, and the four limbs; **l**). Data are shown as the mean ± s.e.m. form *n* = 24 (**k**), 30 (**l**; control) or 32 (**l**; aldometanib-treated) mice for each treatment. *P* values were determined by two-sided Student’s *t*-test. Experiments were performed three times, except **d** and **e** (four times). Source data

**Extended Data Fig. 8:**
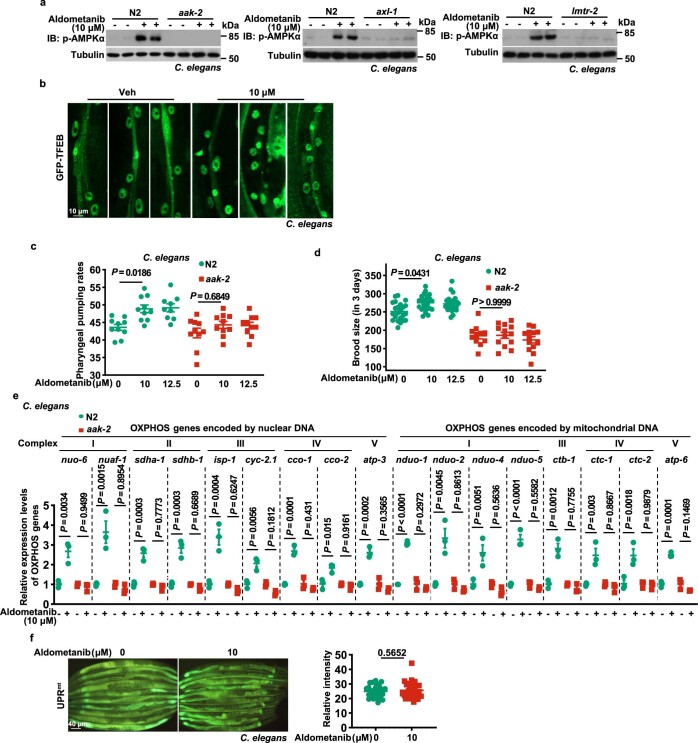
Aldometanib improves lifespan and healthspan in *C*. *elegans*. **a**, aldometanib activates AMPK in *C*. *elegans* via the lysosomal pathway. N2, *aak*-*2* (left), *lmtr*-*2* (middle), and *axl*-*1* (right) nematodes were cultured on the agar plates containing aldometanib at 10 μM, followed by determining levels of p-AMPKα by immunoblotting. **b**, aldometanib inhibits TORC1 in *C*. *elegans*. The N2 nematodes (HLH-30::GFP) expressing GFP-tagged HLH-30, nematode orthologue of TFEB, were cultured on the agar plates containing 10 μM aldometanib for 1 day, followed by determining the localisation of HLH-30 by fluorescent staining. Representative images are shown. **c**, **d**, aldometanib promotes pharyngeal pumping rates and brood size of *C*. *elegans*. N2 and *aak*-*2* nematodes were cultured on the agar plates containing aldometanib at 10 μM and 12.5 μM. Pharyngeal pumping rates (**c**) are mean ± s.e.m., *n* = 9 (N2 + 12.5 μM) or 10 (others) biological replicates. Brood size (**d**) are mean ± s.e.m., *n* = 23 (N2), 11 (*aak*-*2*, control) or 13 (*aak*-*2*, aldometanib-treated) biological replicates. *P* values are calculated by two-way ANOVA, followed by Tukey. **e**, aldometanib promotes mitochondrial OXPHOS gene expression in *C*. *elegans*. N2 and *aak*-*2* nematodes were cultured on the agar plates containing aldometanib at 10 μM for a day, followed by determining the levels of OXPHOS genes by RT-PCR. Data are shown as mean ± s.e.m., *n* = 3 biological replicates for each genotype/treatment, and *P* value by two-way ANOVA, followed by Tukey. **f**, aldometanib does not trigger UPR^mt^ in *C*. *elegans*. The *hsp-6*_*p*_*::gfp* nematodes were cultured on the agar plates containing aldometanib at 10 μM for a day, followed by determining the fluorescent intensities of GFP. Representative images are shown on the left panel, and statistical analysis data on the right panel (left panel; shown as mean ± s.e.m., *n* = 30 worms for each treatment, and *P* value by two-sided Student’s *t*-test with Welch’s correction). Experiments in this figure were performed three times. Source data

**Extended Data Fig. 9:**
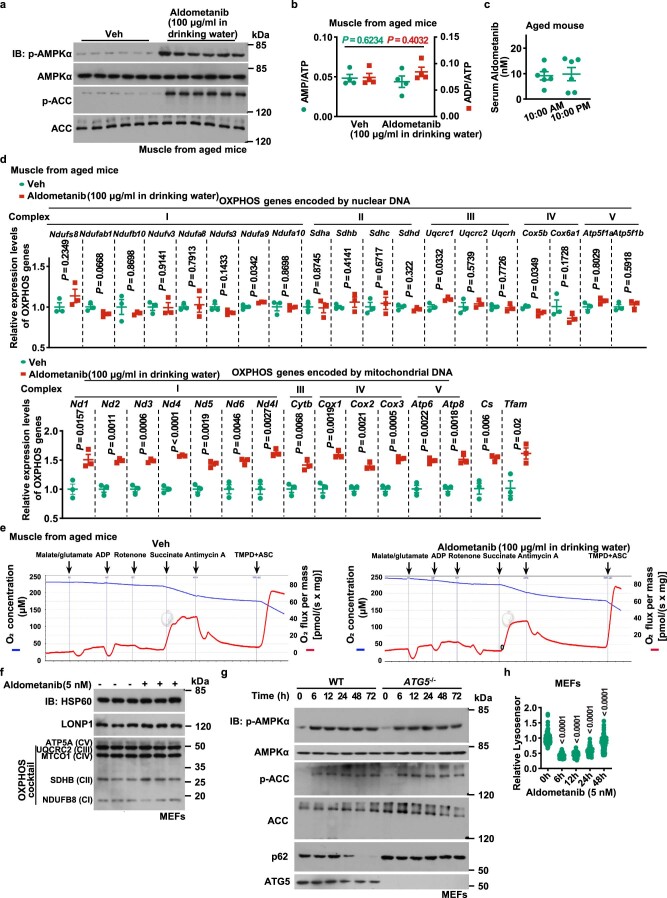
Aldometanib improves lifespan and healthspan in mice. **a**, **b**, aldometanib activates muscular AMPK in aged mice, without elevating AMP. Mice at 52 weeks of age (aged mice) were treated with aldometanib at 100 μg/ml in drinking water for a week, followed by determining levels of p-AMPKα and p-ACC (**a**), as well as AMP:ATP and ADP:ATP ratios (**b**, results are mean ± s.e.m.; *n* = 4 mice for each treatment, and *P* value by two-sided Student’s *t*-test) in muscle tissues. **c**, Serum aldometanib concentrations in aged mice. Aged mice were treated as in **a**, followed by determining serum aldometanib concentration at 10:00 a.m. and 10:00 p.m., respectively. Data are shown as mean ± s.e.m.; *n* = 6 mice. **d**, aldometanib promotes mitochondrial OXPHOS gene expression in mice. Aged mice were treated with aldometanib at 100 μg/ml in drinking water for 4 months, followed by determining the levels of OXPHOS genes by RT-PCR. Data are shown as mean ± s.e.m., *n* = 3 for each genotype/treatment, and *P* value by two-sided Student’s *t*-test. **e**, aldometanib elevates mitochondrial oxidative respiration in aged mice. Mice were treated as in **d**, followed by determining the muscular oxygen consumption rates by the Oxygraph-2k. Representative oxygraphs are shown. **f**, aldometanib does not trigger UPR^mt^ in mammalian cells. MEFs were treated 5 nM aldometanib for 24 h, followed by determining the protein levels of LONP1, HSP60 and MTCO1, makers of UPR^mt^ by immunoblotting. **g**, aldometanib promotes autophagy at later than AMPK activation. MEFs with *ATG5* knocked out, along with its wildtype control, were incubated with 5 nM aldometanib for indicated time periods, followed by determining levels of p62, p-AMPK and p-ACC by immunoblotting. **h**, Long-term treatment of aldometanib re-acidifies lysosomes. MEFs were incubated with 5 nM aldometanib for indicated time periods, followed by determining the intensities of lysosensor. Results are mean ± s.e.m.; *n* = 133 (0 h), 138 (6 h), 129 (12 h), 121 (24 h) or 126 (48 h) cells; and *P* value by one-way ANOVA followed by Dunnet. Experiments in this figure were performed three times. Source data

Finally, we investigated whether aldometanib would extend lifespan in nematodes and mice, which are associated with activation of AMPK and inhibition of TORC1. We cultured *C*. *elegans* on agar and found that aldometanib at 10 μM dissolved in the culture agar activated AMPK without elevating AMP levels (Fig. 8b and Extended Data Fig. 8a). Consistently, we observed a nuclear localization of HLH-30, the nematode ortholog of transcription factor EB (TFEB) that translocates to the nucleus following TORC1 inhibition^83^, under aldometanib treatment (Extended Data Fig. 8b). Such cultured nematodes showed a significant extension of lifespan from a median age of 18 to 26 d (Fig. 8a and Supplementary Table 2). Knockout of *aak*-*2*, *axl*-*1* or *lmtr*-*2*, the nematode orthologues for AMPKα2, AXIN or LAMTOR2, respectively, all abrogated the effects of aldometanib on lifespan extension (Fig. 8c and Extended Data Fig. 8a). We also observed significantly enhanced pharyngeal pumping rates, oxidative stress resistance and brood size (fecundity) in nematodes treated with aldometanib (Fig. 8d, Extended Data Fig. 8c,d and Supplementary Table 2), also indicative of an anti-ageing effect of aldometanib. Aldometanib also significantly elevated NAD^+^ levels in wild-type nematodes, but not in those lacking *aak*-*2* (Fig. 8e). Consistently, significant increases of ratio of mitochondrial to nuclear DNA (mtDNA:nDNA), gene expression of 17 mitochondrial genes and mitochondrial respiration rates (oxygen consumption rates (OCRs)) were observed in the aldometanib-treated nematodes (Fig. 8f,g and Extended Data Fig. 8e). Knockout of *aak*-*2* blocked all of these effects (Fig. 8f,g and Extended Data Fig. 8e), indicating that the aldometanib-enhanced mitochondrial functions in nematodes depend on AMPK. Note that although aldometanib increased the ratios of mtDNA:nDNA, it did not induce the mitochondrial unfolded protein response (UPR^mt^), as evidenced by the unchanged green fluorescence intensity in GFP-tagged, hsp-6-expressing nematodes, which is a reporter strain for UPR^mt^ (Extended Data Fig. 8f). The absence of UPR^mt^ is similar to the case of low-dose metformin, in which 50 mM metformin (dissolved in the medium agar) activates AMPK in nematodes through the AMP-independent lysosomal pathway, but failed to elicit UPR^mt^ (refs. ^62,84^). We also examined the effects of aldometanib on lifespan in mice. Titrating different doses, we found that aged (1-year-old) mice treated with aldometanib at 100 μg ml^−1^ in drinking water, which showed 8 nM of aldometanib in the serum (similarly to those from oral gavaging at 2 mpk), showed activation of AMPK in skeletal muscle without elevated AMP levels (Extended Data Fig. 9a–c). We observed a significant increase of lifespan (7.4% in male mice, and 7.7% in female mice) in these mice (Fig. 8h and Supplementary Table 2). Similarly to our observations in nematodes, we detected a significant increase of NAD^+^ levels, mtDNA:nDNA ratios (without inducing UPR^mt^), gene expression of mtDNA-encoded mitochondrial genes (particularly those comprising mitochondrial complex I, as described previously^85^) and (the complex I-supported) OCRs, in muscle tissues of aldometanib-treated mice (Fig. 8i,j and Extended Data Fig. 9d–f). Running distance, duration and grip strength were also significantly increased in 1-year-old mice treated with aldometanib (Fig. 8k,l).

**Extended Data Fig. 10:**
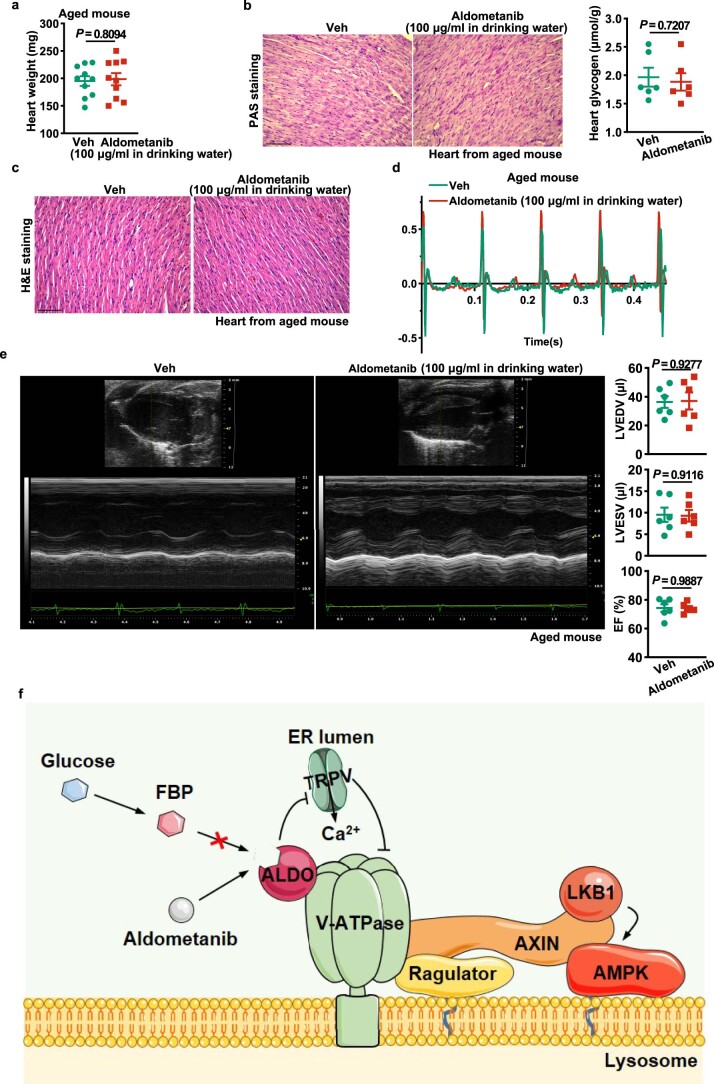
Aldometanib does not cause cardiac hypertrophy. **a**-**e**, Aged mice were treated with aldometanib at 100 μg/ml in drinking water for 4 months, followed by determining heart weight (**a**; data are mean ± s.e.m., *n* = 10 mice for each treatment, and *P* value by two-sided Student’s *t*-test), heart glycogen content (**b**, right panel; data are mean ± s.e.m., *n* = 6 mice for each/treatment, and *P* value by two-sided Student’s *t*-test; see also representative images from PAS staining of heart in the left panel), heart morphology (**c**; by H&E staining), cardiac function [assessed by electrocardiography (**d**) and echocardiography (**e**; representative echocardiograms are shown; see also levels of LVEDV, LVSEV and the percentage of EF; data are mean ± s.e.m., *n* = 6 mice for each treatment, and *P* value by two-sided Student’s *t*-test)]. **f**, Schematic diagram showing that aldometanib engenders a pseudostarvation of glucose to activate lysosomal AMPK. In high glucose, the glycolytic intermediate FBP occupies aldolase via the key residue K230. In low glucose, however, aldolase becomes unoccupied, and inhibits the cation channel TRPV, the latter of which then disrupts the association of the former with, and inhibits, v-ATPase. aldometanib binds aldolase K230 to prevent the binding of FBP, mimicking the state of low glucose. The inhibited v-ATPase, along with its associated Ragulator, provides docking sites for AXIN/LKB1, leading to the activation of AMPK on the lysosome. This panel was modified from elements created by Servier Medical Art (https://smart.servier.com/). Experiments in this figure were performed three times, except **d** and **e** four times. Source data

It is unclear how autophagy can still take place in aldometanib-treated cells. On one hand, aldometanib-activated AMPK may promote autophagy, which has been shown to play a role in AMPK-mediated lifespan extension^32,86^. On the other hand, aldometanib inhibits v-ATPase, which should abrogate acidification of lysosomes, which is required in the initiation of autophagy^87^. As shown in Extended Data Fig. 9g, we found an intact autophagy induction in cells treated with aldometanib, but at much later times than that of v-ATPase inhibition and AMPK activation: (a) the deacidification of lysosomes and the increase of p-AMPK signals emerged after 1 h of aldometanib treatment and p-AMPK signals remained relatively constant up to 72 h; and (b) the decrease of p62, as an autophagy indicator, became obvious only after 12 h of aldometanib treatment, which was then accompanied with re-emergence of the LysoSensor signal (Extended Data Fig. 9h). This pattern of AMPK activation and autophagy induction was also observed in cells that were starved for glucose or treated with metformin^9,62^, and the recovered LysoSensor signal might stem from newly formed lysosomes. Of note, mice treated with aldometanib did not exhibit changes of heart weight, or display changes in heart glycogen content (determined by PAS staining and biochemical assay) or morphological appearance (by H&E staining; Extended Data Fig. 10a–c), indicating that aldometanib does not cause cardiac hypertrophy as seen in mice treated with direct AMPK agonists^37^. Consistently, no electrocardiogram abnormality (increase in the amplitude of QRS, elongation of QRS or shortening of PR interval) or cardiac dysfunction (changes in ejection fraction and stroke volume) was observed in aldometanib-treated mice (Extended Data Fig. 10d,e).

## Discussion

Here, we identified a new inhibitor of aldolase, referred to as aldometanib, which activates AMPK by preventing aldolase from binding to FBP to engender a pseudo-starvation signal (Extended Data Fig. 10f). Unlike direct allosteric activators of AMPK, aldometanib specifically activates the lysosomal pool of AMPK via the intrinsic cellular signalling route that serves to sense physiological decline of glucose occurring during the starvation and calorie restriction. Such a local activation of AMPK may underlie the beneficial effects without causing adverse effects^36^. Indeed, we show that aldometanib effectively promotes glucose absorption by promoting the activation of muscular AMPK and inhibits lipogenesis by hepatic AMPK, thereby improving glucose homeostasis and alleviating fatty liver and NASH. In particular, long-term administration of aldometanib also significantly extends lifespan and healthspan of nematodes and mice, without causing discernible side effects such as cardiac hypertrophy. By contrast, indiscriminate AMPK activation, either by pan-AMPK activators or naturally occurring mutations in the PRKAG2 gene encoding the AMPKγ2 subunit, tends to cause cardiac disorders^37,88–90^. However, two other AMPK agonists, salsalate (a pro-drug of salicylic acid) and PXL770, that show the efficacy of lowering blood glucose and alleviating NAFLD^48,91,92^ have been under clinical trials and have shown favourable safety profiles^93–96^. Interestingly, both of the two agonists are AMPKβ1 specific, leaving the AMPKβ2-comprised AMPK complex inactivated^48,92^.

The specific activation of lysosomal AMPK by aldometanib is attributed to its serendipitous enrichment in the lysosome, which is supported by multiple lines of evidence. For example, subcellular fractionation assays showed that aldometanib could only be detected in the lysosomal fraction, but not ER or mitochondria (Fig. 2d). In addition, the enrichment fold of the average concentration of lysosomal aldometanib over that in the total cell lysates is about 150-fold (the total cellular concentration is 0.004 μg of aldometanib per gram of total cellular protein and the proteins of purified lysosomes were estimated to be approximately 0.65% of the total cellular protein; refs. ^15,69^). Moreover, data in Fig. 2d showed that the total amount of lysosomal aldometanib is similar to that detected in the total cell homogenates. Although we still do not know the entire mechanisms inside a live cell, the membrane composition of the lysosome, protein(s) other than aldolase on the lysosome, and the local biophysical natures of the lysosome–ER contact (for example, local pH) are possibly among the contributing factors, in line with the evidence that the presence of intact lysosomes significantly lowered the IC_50_ of aldometanib for aldolase, and that the cells in which lysosomes were deacidified showed impaired lysosomal accumulation of aldometanib. In addition, the molecular features, particularly the acyl chain of aldometanib, might also be involved in the lysosomal enrichment of aldometanib. Although still largely unknown at the molecular level, the accumulation of chemicals with the acyl chains of compounds in a specific organelle inside cells have also been observed for some other lipid molecules. For example, it was shown that C16:0 ceramides (particularly those synthesized by CerS6) tend to be localized in mitochondria and mitochondria-associated membranes^97^, while C22:0 ceramides are in lysosomes^98^. It is therefore reasonable to speculate that a complex network of other factors may collectively contribute to the accumulation of aldometanib in the lysosome. In comparison, other aldolase inhibitors, such as TDZD-8 (ref. ^99^), UM0112176 (ref. ^100^) and LYG-202 (ref. ^101^) impair glycolysis likely through inhibiting all pools of aldolase, and are able to cause cancer cell death. Together, these features of aldometanib endow it with a potential to be developed into a drug for the treatment of metabolic diseases in humans.

## Methods

### Data reporting

The chosen sample sizes were similar to those used in this field: *n* = 4–8 mice or rats were used to determine the effects of AMPK activators (for example, metformin and MK-8722) on blood glucose^37,102^, fatty liver^73,75^ and NASH^48,78^; *n* = 100–200 worms and mice were used to determine lifespan^103–105^; *n* = 9–23 worms and 6–32 mice were used to determine healthspan^106–108^; *n* = 3–6 mice or rats were used to determine the pharmacokinetics of aldometanib^71^; *n* = 18–35 cells from 3–8 dishes/fields were included for conclusions based on immunofluorescence staining (IF)^14,15^; *n* = 3–10 samples were used for evaluation of the levels of metabolites in cells and tissues^9,14,15^; *n* = 3 samples were used to determine the expression levels and phosphorylation levels of a specific protein^13^; *n* = 3 samples were used to determine the mRNA levels of a specific gene^13,73^; and *n* = 3–4 samples were used to determine the activity of aldolase in vitro^109^. No statistical methods were used to predetermine sample size. All experimental findings were repeated as stated in the figure legends, and all additional replication attempts were successful. For animal experiments, mice, rats or nematodes were housed under the same condition or place. For cell experiments, cells of each genotype were cultured in the same condition and were seeded in parallel for different treatments. Each experiment was designed and performed along with proper controls, and samples for comparison were collected and analysed under the same conditions. Randomization was applied wherever possible. For example, during MS analyses (metabolites, proteins and pharmacokinetics), samples were processed and subjected to the mass spectrometer in random orders. For animal experiments, sex-matched (only for rodents), age-matched littermate animals in each genotype were randomly assigned to pharmacological treatments. In cell experiments, cells of each genotype were randomly assigned to different treatments. Otherwise, randomization was not performed. For example, when performing IB, samples needed to be loaded in a specific order to generate the final figures. Blinding was applied wherever possible. For example, samples, cages or agar plates during sample collection and processing were labelled as code names that were later revealed by the individual who picked and treated animals or cells, but who did not participate in sample collection and processing, until assessing outcome. Similarly, during microscopy data collection and statistical analyses, the fields of view were chosen on a random basis, and were often performed by different operators, preventing potentially biased selection for desired phenotypes. Otherwise, blinding was not performed, such as during the measurement of aldolase activity in vitro, as different reagents were added for particular reactions.

### Synthesis of aldometanib

Aldometanib was synthesized from 2-methyl-1*H*-imidazole (see detailed procedures in Supplementary Information) and was obtained as white powder. The structures of the synthesized products were determined by nuclear magnetic resonance (NMR) and MS. ^1^H NMR (600 MHz, Chloroform-*d*) δ 7.63 (d, *J* = 2.2 Hz, 1H), 7.48–7.44 (m, 2H), 7.41 (dd, *J* = 9.3, 6.6 Hz, 1H), 6.96 (d, *J* = 2.3 Hz, 1H), 5.65 (s, 2H), 4.30 (t, *J* = 7.5 Hz, 2H), 3.03 (d, *J* = 2.2 Hz, 3H), 1.90–1.84 (m, 2H), 1.36–1.19 (m, 26H), 0.88 (t, *J* = 7.0 Hz, 3H). ^13^C NMR (150 MHz, CDCl_3_) δ 144.13, 136.69, 132.14, 129.27, 128.41, 122.31, 120.07, 49.53, 48.27, 31.90, 29.82, 29.68–29.50 (overlapping), 29.39, 29.34, 29.05, 26.31, 22.66, 14.11, 12.56. MS (ESI) *m/z*: 466[M]^+^. High-resolution MS coupled with electrospray ionization (ESI) was calculated for C_27_H_43_N_2_Cl_2_ [M]^+^, 465.2798; found, 465.2798.

### Formulation of aldometanib

For cell-based experiments, aldometanib powder was dissolved in DMSO, and was aliquoted and stored at 4 °C. The solution was incubated in a 37 °C water bath for 10 min (until no precipitate was visible) before adding to the culture medium. For rodent experiments, aldometanib was formulated in 10% (wt/vol) Kolliphor HS 15 within 1 week before the experiment. Briefly, to prepare 3 l of 1 mg ml^−1^ aldometanib stock solution, 3 g of aldometanib was dissolved in 300 ml of ethanol, followed by mixing with 300 g of Kolliphor HS 15. The mixture was stirred at room temperature until Kolliphor HS 15 was completely dissolved. The ethanol in the solution was then evaporated on a rotary evaporator (Rotavapor R-300, BUCHI) at 90 r.p.m., at 45 °C, followed by mixing with 2,700 ml of water. The control vehicle was similarly prepared, with no aldometanib added to the ethanol–Kolliphor HS 15 solution. Both the aldometanib solution and the vehicle were stored at room temperature. For nematode experiments, aldometanib was freshly dissolved in ethanol and was added to warm (cooled to approximately 60 °C after autoclaving) nematode growth medium (NGM, containing 0.3% (wt/vol) NaCl, 0.25% (wt/vol) bacteriological peptone, 1 mM CaCl_2_, 1 mM MgSO_4_, 25 mM KH_2_PO_4_-K_2_HPO_4_, pH 6.0, 0.02% (wt/vol) streptomycin, and 5 μg ml^−1^ cholesterol) supplemented with 1.7% (wt/vol) agar before pouring to make the NGM plates. The plates were stored at 20 °C.

### Rodent strains

Protocols for all rodent experiments were approved by the Institutional Animal Care and the Animal Committee of Xiamen University (XMULAC20180028). Wild-type C57BL/6J mice (000664) were obtained from The Jackson Laboratory. *Axin*^*fl/fl*^ and *Lamtor1*^*fl/fl*^ mice were generated and maintained as described previously^13^. *Trpv1*^−/−^ mice were obtained from The Jackson Laboratory provided by D. Julius (003770). *Trpv1*^−/−^ mice with knockdown of *TRPV2*–*TRPV4* or *GFP* were generated as described previously^15^. *Ampkα1*^*fl/fl*^ (Jackson Laboratory, 014141) and *Ampkα2*^*fl/fl*^ mice (Jackson Laboratory, 014142) were obtained from Jackson Laboratory, provided by S. Morrison. *Camkk2*^−/−^ mice (MGI: 4941485) were obtained from The Jackson Laboratory, provided by T. Chatila. *Db/db* (*Lepr*^db^) mice were obtained from The Jackson Laboratory (000697). *Lkb1*^fl/fl^ mice (MGI: 2387402) were obtained from Frederick National Laboratory for Cancer Research, provided by R. DePinho (BRC no. RBRC02975). *Atg5*^fl/fl^ mice were obtained from RIKEN, provided by N. Mizushima (BRC no. RBRC02975). *Lamtor1*^fl/fl^ and *AMPKα1*/*2*^fl/fl^ mice were crossed with *Mck*-*Cre* or *Alb-Cre* mice to generate muscle-specific knockout (*Lamtor1*-MKO) or liver-specific *Lamtor1* knockout (*LAMTOR1*-LKO) mice, and muscle-specific (*Ampkα*-MKO) or liver-specific *Ampkα* knockout (*Ampkα*-LKO) mice. *AMPKα1*/*2*^fl/fl^ were also crossed with *Alb-CreERT2* mice to generate inducible liver-specific *Ampkα* knockout mice (for deleting AMPK in established fatty liver and NASH mouse models to analyse the roles of AMPK, after activation by aldometanib, in fatty liver or NASH alleviation), in which hepatic *AMPKα* was deleted by intraperitoneally injecting the *Alb-CreERT2*-carrying *Ampkα1*/*2*^fl/fl^ mice (fed with HFD for 16 weeks, or AMLN diet for 30 weeks) with tamoxifen (dissolved in corn oil) at 200 mpk, three times a week. Knockout efficiencies were analysed at 1 week after the last injection by western blotting. All mouse strains used in this study were on the C57BL/6J background. ZDF rats (123) were obtained from Vital River Laboratory Animal Technology (Beijing, China), a branch of Charles River Laboratories. In this study, only male rodents were used, except in Fig. 8h, where both male and female mice were used. Unless stated otherwise (for example, aged mice for lifespan determination), rodents at 4 weeks old were used.

### Aldometanib treatment in mice and rats

Unless stated otherwise, mice (3–4 per 385 × 210 × 174 mm^3^ cage) and rats (2 per 482 × 336 × 268 mm^3^ cage) were housed with free access to water and standard diet (65% (wt/wt) carbohydrate, 11% (wt/wt) fat and 24% (wt/wt) protein). The light was on from 08.00 to 20.00, with the temperature kept at 21–24 °C and humidity at 40–70%. Except those used for determining lifespans, only male mice and rats were used in the study. Littermate controls were used throughout the study. Aldometanib was supplied either through oral gavaging or in drinking water. For determining the acute, glucose-lowering effects of aldometanib on normoglycaemic (lean) rodents, 4-week-old C57BL/6 mice or ZDF rats were used. For creating the diabetic mouse model, C57BL/6 mice were fed with a HFD (60% calories from fat; D12492, Research Diets) for 4 months starting at 4 weeks old, and *db/db* mice were fed with a standard diet for 3 months starting at 4 weeks old. For creating the diabetic rat model, ZDF rats were fed with a HFD for 3 months starting at 8 weeks old. For creating the NASH model, C57BL/6 mice were fed with an AMLN diet (40% calories from fat, 20% calories from fructose, and 2% (wt/wt) cholesterol; D09100301, Research Diets) for 30 weeks starting at 4 weeks old.

### Evaluation of mouse lifespan

Mouse lifespan was determined according to a previous report^110^, with minor modifications. Briefly, mice were examined every morning for signs of illness, and were killed and recorded if they were severely moribund. A mouse was considered severely moribund if it showed more than one of the following clinical signs: (a) inability to eat or to drink; (b) severe lethargy, as indicated by lack of response such as a reluctance to move when gently prodded with a blunt-tip tweezer; (c) severe balance instability or gait disturbance; (d) rapid weight loss (>3 g) over a period of 1 week; or (e) a severely ulcerated or bleeding tumour. Mice found dead were also noted at each daily inspection.

In the cohort shown in Fig. 8h and Supplementary Table 2, a total of 577 mice (380 males and 197 females) were initially included. Of which, 46 mice (34 males and 12 females) were removed (censored) from the study because of fighting (25 males), accidental injury or death (cage flooding, tail clipped by the cage lid, and so on; 5 males and 8 females), or because of technical error (error in drug supply; 4 males and 4 females). Such censored mice were not included in the calculation of lifespan.

### Determination of mouse running capacity and grip strength

The maximal running capacity was determined as described previously^111^, with minor modifications. Briefly, mice were trained on Rodent Treadmill NG (Ugo Basile, 47300) at 10 m min^−1^ for 5 min for 2 d with a normal light–dark cycle, and tests were performed during the dark period. Before the experiment, mice were fasted for 2 h. The treadmill was set at a 15° incline, and the speed of the treadmill was set to increase in a ramp mode (10 m min^−1^ for 10 min followed by an increase to a final speed of 18 m min^−1^ within 15 min). Mice were considered to be exhausted, and removed from the treadmill, following the accumulation of five or more shocks (0.1 mA) per minute for two consecutive minutes. The distances travelled were recorded as the running capacity.

Grip strength was determined on a grip strength meter (Ugo Basile, 47200) following the protocol described previously^108^. Briefly, the mouse was held by its tail and lowered (‘landed’) until all four limbs grasped the T‐bar connected to a digital force gauge. The mouse was further lowered to the extent that the body was horizontal to the apparatus, and was then slowly, steady drawn away from the T‐bar until all four limbs were removed from the bar, which gave rise to the peak force in grams. The experiment for each mouse was repeated five times with 5-min intervals between measurements.

### Serology and glucose and insulin tolerance tests

Mice or rats were individually caged for a week before each experiment. For determining the effects of aldometanib on fasting glucose, mice were fasted for 2 h (8:00 to 10:00), while rats were fasted for 6 h (8:00 to 14:00). Blood glucose was measured at the indicated time points through tail vein bleeding using the OneTouch UltraVue automatic Glucometer (LifeScan). For GTT, mice or rats were fasted for 6 h (8:00 to 14:00), then administered with glucose at 1.5 g per kg body weight (for lean mice, intraperitoneally injected), 1 g per kg body weight (for diabetic mice, or mice with NASH, intraperitoneally injected), or 2 g per kg body weight (for rats, orally gavaged). For ITT, mice or rats were treated as in GTT, except that 1 U kg body weight of insulin was intraperitoneally injected. Effects of aldometanib on fasting glucose, GTT and ITT were analysed using different batches of rodents to avoid the interference from any stress caused by earlier blood collection.

For measuring insulin levels, approximately 100 μl of blood was collected at each time point (from submandibular vein plexus), and was placed at room temperature for 20 min, followed by centrifugation at 3,000*g* for 10 min at 4 °C. Some 5 μl of the resultant serum was used to determine the levels of insulin using the Mouse or Rat Ultrasensitive Insulin ELISA kit according to the manufacturer’s instructions. The five-parameter logistic fitted standard curve for calculating the concentrations of insulin was generated from the website of Arigo Biolaboratories (https://www.arigobio.cn/ELISA-calculator/). For measuring ALT, aspartate transaminase (AST) and ALP levels, serum samples were prepared according to those used for insulin measurements. Some 10 μl, 50 μl and 100 μl of serum were used to determine the levels of ALT, AST and ALP, respectively, using kits according to the manufacturer’s instructions.

For measuring serum TAG contents during the olive oil gavage (to determine intestinal fat absorption rate, as described previously^112^), mice were starved for 8 h (0:00 to 8:00), and were intravenously (via tail vein) injected with 500 mpk tyloxapol (prepared by diluting tyloxapol in PBS to a 50 mg ml^−1^ stock solution), a lipoprotein lipase inhibitor, to prevent plasma TAG hydrolysis during olive oil gavage assay at 30 min before oil gavage. Some 10 μl of serum was used to determine serum TAG level using LabAssay triglyceride reagent.

### Glucose clamps

The hyperinsulinaemic–euglycaemic clamp technique was performed as described previously^113^, in which a two-phase protocol consisting of a 120-min equilibration period (*t* = −120 min to 0 min) and a 120-min experimental period (*t* = 0 min to 120 min) was applied. Mice were cannulated on their right jugular veins to establish a catheter for glucose infusion at 5 d before the experiment. On the day of experiment, mice were fasted for 6 h (8:00 to 14:00) in a restrainer. Blood samples (collected from tail vein) were obtained at *t* = −120 min, followed by determining the initial glucose level. Some 5 μCi bolus of [3-^3^H]-glucose was given at *t* = −120 min, and was infused for 120 min at a rate of 0.05 μCi min^−1^. At *t* = −30 min and 0 min, blood samples were taken for the assessment of basal levels of glucose and insulin, and the rates of glucose turnover. The clamp began at *t* = 0 min with a prime-continuous infusion of insulin (300 mU/kg priming, followed by 2.5 mU/kg min^−1^ continuous infusion). The [3-^3^H]-glucose infusion rate was then increased to 0.1 μCi min^−1^ during the remaining time of the experiment. Euglycaemia (6.5–7.5 mM) was maintained during clamping by measuring blood glucose every 10 min (starting at *t* = 0 min) and infusing 20% glucose when necessary. Blood samples were taken every 10 min from *t* = 80 min to 120 min for determining the radioactive activities of glucose.

The hyperglycaemic clamp technique was performed as described previously^114^. Briefly, an individual 6-h-fasted mouse housed in a restrainer was infused with 20% glucose until plasma glucose levels reached 16–18 mM. Blood samples (50 μl each) were collected from the tail vein during the clamp, and glucose levels were measured.

### Histology

For H&E staining of liver tissues, tissues excised from blood-drained mice or rats were cut into pieces, and were fixed in 4% (vol/vol) paraformaldehyde for 24 h at room temperature, then transferred to embedding cassettes. The cassettes were then washed in running water for 12 h, followed by successive soaking each for 1 h in 70% ethanol (vol/vol in water), 80% ethanol and 95% ethanol. The fixed tissues were further dehydrated in anhydrous ethanol for 1 h twice, followed by immersing in 50% xylene (vol/vol in ethanol) for 30 min, two changes of xylene, 15 min each; and two changes of paraffin wax (58–60 °C), 1 h each. The dehydrated tissues were embedded in paraffin on a HistoCore Arcadia Paraffin Embedding Machine (Leica). Paraffin blocks were then sectioned at a thickness of 3 μm, dried on an adhesion microscope slide, followed by rehydrating in the following order: two changes of xylene at 70 °C, 10 min each; two changes of anhydrous ethanol, 5 min each; two changes of 95% ethanol, 5 min each; one change each for 5 min of 80% ethanol, 70% ethanol, and 50% ethanol, and then briefly in water. The sections were then stained in haematoxylin solution for 8 min, then washed in running water for 5 min, differentiated in 1% hydrochloric acid (in ethanol) for 30 s, washed in running water for 1 min, and immersed in 0.2% (vol/vol in water) ammonium hydroxide solution for 30 s, washed in running water for 1 min, and stained in eosin Y-solution for 30 s. The stained sections were dehydrated in 70% ethanol for 5 min; twice in 95% ethanol, 5 min each; twice in anhydrous ethanol, 5 min each; and two changes of xylene, 15 min each. The stained sections were mounted with Canada balsam and visualized on a Leica DM4 B.

The NASs and individual histological scores for steatosis grade, hepatocellular ballooning and lobular inflammation were determined based on the observation of microscopic images taken from the H&E-stained liver sections of NASH mice as described previously^81,82^. Briefly, steatosis grade was determined by the percentage of lipid droplets in the total tissue area, lobular inflammation was determined by the number of inflammatory foci, and hepatocellular ballooning was determined by the number of ballooned hepatocytes. NASs were obtained from the sum of the above three scores.

For Sirius Red staining, the liver tissues were fixed, dehydrated, embedded, sectioned and rehydrated as in H&E staining, and were then stained using the Picro Sirius Red Stain kit according to the manufacturer’s instructions. Briefly, sections were stained with Picro Sirius Red Solution for 60 min, and then quickly rinsed in two changes of acetic acid solution, followed by another three changes of ethanol. The stained sections were mounted with Canada balsam and visualized on a Leica DM4 B.

For H&E staining of iWAT, tissues were fixed, dehydrated, embedded, sectioned, rehydrated and stained as in H&E staining of liver tissues, except that the tissue sections were stained in haematoxylin solution for 2 min, and eosin Y-solution for 1 min.

For H&E staining of heart tissues, tissues were washed in two changes of PBS, and then fixed in 4% (vol/vol) paraformaldehyde for 48 h at 4 °C before transferring to embedding cassettes. The cassettes were washed with one change of distilled water, and were dehydrated according to the following steps: soaked in 50% ethanol (vol/vol in water) for 1 h, 70% ethanol for 1 h and 80% ethanol for 40 min; two changes of 95% ethanol, 40 min each; and two changes of anhydrous ethanol, 1 h each. The cassettes were then soaked in 50% xylene (vol/vol in ethanol) for 30 min, two changes of xylene, 15 min each; and two changes of paraffin wax (58–60 °C), 1 h each. Tissues were embedded and sectioned in the same way as for liver tissues, except that the thickness of the sections was 5 μm. Each section was placed in 10% ethanol at room temperature for 3 min, and was then transferred (using a nonadhesive glass slide) to a 42 °C water bath for another 5 min of incubation. Sections were dried on an adherent glass slide, and were incubated in a 45 °C hot-air oven overnight. The dried sections were deparaffinized and rehydrated in the same way as those for liver tissues, and were stained in haematoxylin solution for 40 s, then washed in running water for 5 min, and directly stained with eosin Y-solution for 20 s. The stained sections were dehydrated in 50% ethanol for 30 s, 70% ethanol for 30 s, 80% ethanol for 30 s and 95% ethanol for 30 s; twice in anhydrous ethanol, 5 min each; and two changes of xylene, 5 min each. The stained sections were mounted with Canada balsam and visualized on a Leica DM4 B.

For periodic acid–Schiff (PAS) staining, heart tissues were fixed, dehydrated, embedded, sectioned and rehydrated as in H&E staining, and were then stained using the PAS Staining System Kit according to the manufacturer’s instructions. Briefly, sections were incubated in periodic acid solution for 5 min at room temperature, followed by rinsing with three changes of distilled water. Sections were then immersed in Schiff’s Reagent for 15 min at room temperature, followed by washing with running water for 5 min, and then stained in haematoxylin solution, gill no. 3, for another 90 s. Sections were quickly washed with running water, and then dehydrated in 90% ethanol for 2 min and anhydrous ethanol for another 2 min. Sections were then incubated with two changes of xylene, 5 min each. The stained sections were mounted with Canada balsam and visualized on a Leica DM4 B.

For immunohistochemistry (IHC) staining of F4/80, liver tissues were fixed, dehydrated, embedded, sectioned and rehydrated as in H&E staining, and were washed with PBS twice, 5 min each at room temperature. The sections were then incubated in preheated (approximately 95 °C) Tris-EDTA buffer (10 mM Tris, pH 9.0, 1 mM EDTA, 0.05% (vol/vol) Tween-20) for 10 min, followed by cooling at room temperature for another 30 min. The sections were then washed with TBS buffer supplemented with Triton X-100 (20 mM Tris, pH 7.6, 150 mM NaCl, 0.025 (vol/vol) Triton X-100) twice, 5 min each at room temperature, and then incubated in 3% (wt/wt) H_2_O_2_ (in methanol) solution at room temperature for 30 min, followed by washing with TBS buffer supplemented with Triton X-100 twice, 5 min each at room temperature. The sections were then incubated in 1% (wt/vol) BSA (dissolved in TBS buffer supplemented with Triton X-100) solution at room temperature for 2 h. After draining off BSA solution, the liver sections were circled by a PAP pen (Z377821, Sigma), and were incubated with rabbit anti-F4/80 antibody (diluted in 1% BSA solution) for 16 h at 4 °C in a dark, humidified chamber, followed by washing with TBS buffer supplemented with Triton X-100 for three times, 5 min each at room temperature. The sections were then incubated with Alexa Fluor 488 goat anti-rabbit IgG (diluted in 1% BSA solution) for 6 h at room temperature in a dark, humidified chamber, followed by washing with TBS buffer supplemented with Triton X-100 for three times, 5 min each at room temperature. After draining off TBS buffer supplemented with Triton X-100, sections were mounted with DAPI Fluoromount-G mountant and visualized on a Leica DM4 B.

For TUNEL/BrdU staining, the liver tissues were fixed, dehydrated, embedded, sectioned and rehydrated as in H&E staining, and were stained using the Apo-BrdU In Situ DNA Fragmentation Assay Kit according to the manufacturer’s instructions. Briefly, the rehydrated sections were incubated in 0.85% for 5 min, PBS for 5 min, and 4% (vol/vol) formaldehyde for 15 min, all at room temperature. The sections were rinsed with PBS twice, 5 min each at room temperature, followed by incubation in 20 μg ml^−1^ of proteinase K solution (prepared by mixing 2 μl of 10 mg ml^−1^ protease K with 998 μl of 100 mM Tris-HCl, pH 8.0 and 50 mM EDTA to generate a 1-ml solution) and rinsing with PBS, 5 min each at room temperature. The sections were then fixed with 4% (vol/vol) formaldehyde for 5 min at room temperature, followed by rinsing with PBS for 5 min at room temperature. The sections were washed with wash buffer twice, 5 min each at room temperature, and then incubated with DNA Labeling Solution (by mixing 10 μl of TdT Reaction Buffer, 0.75 μl of TdT Enzyme, 8 μl of Br-dUTP with 32.25 μl of double-distilled water to generate a 50-μl solution) for 1 h at 37 °C in a dark, humidified chamber. The sections were then washed with PBS twice, 5 min each at room temperature, followed by incubating with Antibody Solution (by mixing 5 μl of anti-BrdU-FITC antibody and 95 μl of rinse buffer) for 0.5 h at room temperature in a dark, humidified chamber. The sections were then incubated with propidium iodide/RNAse A solution for 0.5 h at room temperature in a dark, humidified chamber, and then washed twice with double-distilled water, 5 min each at room temperature. Sections were mounted with ProLong Diamond Antifade Mountant, and were visualized on an LSM 980 (Zeiss) with a ×63 1.4-NA oil objective, during which a diode laser module (BLD-RT 48830 TN01, Lasos) at 488 nm (to excite FITC) and a DPSS laser module (YLK- XT 5948 F01, Lasos) at 594 nm (to excite propidium iodide) were used.

All histochemical images, as mentioned above, were taken and processed by LAS X software (v.3.0.2.16120, Leica), except those of TUNEL/BrdU staining by Zen Blue 3.3 software (Zeiss). Images were formatted by Photoshop 2022 software (Adobe).

For measuring hepatic TAG contents, mice or rats were killed by cervical dislocation, and the livers were immediately removed and rinsed in PBS three times. Some 50 mg of tissue was homogenized by a hand-held homogenizer (T 10 basic ULTRA-TURRAX equipped with an S10N-5G dispersing tool, IKA; and same hereafter, unless stated otherwise) in 1 ml of PBS containing 5% (vol/vol) Triton X-100. The homogenates were boiled for 5 min, followed by centrifugation at 20,000*g* at 25 °C for 10 min. The contents of TAG (the supernatant) were determined using the LabAssay triglyceride reagent.

For measuring faecal TAG contents, approximately 0.1 g of fresh faecal sample was homogenized with 600 μl of chloroform methanol mixture (1:1), followed by mixing with 200 μl of chloroform and 200 μl of water, by 20 s of vortexing each. After centrifugation at 15,000*g* for 15 min at 4 °C, 600 μl of organic (lower) phase was collected, followed by lyophilization with nitrogen blow on a pressured gas blowing concentrator (MGS-2200, EYELA) at room temperature. The lyophilized samples were then dissolved with 100 μl of 1% (vol/vol) Triton X-100, and 20 μl of dissolved samples was used for measuring TAG contents in the same way as for measuring those in the liver tissues.

Levels of hepatic hydroxyproline were measured using the Hydroxyproline Assay kit according to the manufacturer’s instructions. Briefly, 100 mg of liver tissue was homogenized in 1 ml of double-distilled water in a 2-ml Dounce homogenizer (D8938, Sigma). Some 100 μl of homogenate was used for determining hydroxyproline levels.

Cardiac glycogen contents were determined using the Glycogen Assay Kit according to the manufacturer’s instructions. Briefly, mouse heart was quickly excised and immediately frozen in liquid nitrogen. Some 10 mg of heart tissue was homogenized in 100 μl of double-distilled water on ice, then boiled for 5 min, and then centrifuged at 20,000*g* for 10 min. Then, 20 μl of supernatant was mixed with 30 μl of hydrolysis buffer, and then mixed with 2 μl of hydrolysis enzyme mix, followed by incubation for 30 min at room temperature. Next, 50 μl of the master reaction mix (through mixing 46 μl of development buffer, 2 μl of development enzyme mix with 2 μl of fluorescent peroxidase substrate) was added to the sample, followed by incubation for 30 min at room temperature in the dark. The OD_570_ was then recorded by a SpectraMax M5 microplate reader (Molecular Devices) using the SoftMax Pro software (v.5.4.1.1, Molecular Devices; and same hereafter), and the contents of glycogen were then calculated according to the standard curves generated with Glycogen Standards.

### Cardiac function studies

For electrocardiography, mice were anaesthetized by inhaling 2% (vol/vol) isoflurane by means of a laboratory anaesthesia machine (R550, RWD Life Science), and were placed in a supine position on a flat operating desk. The mouse limbs were stuck on the desk with medical tapes, and were inserted with electrodes connected with a PowerLab biological signal acquisition and analysis system (ML4856, ADInstruments) in a three-lead manner. Signals from leads I, II, III, aVR, aVL and aVF were recorded using LabChart (v.8.1.12, ADInstruments), and the PR, QRS and QT intervals, and heart rates were calculated accordingly. The Bazett’s formula was then applied for calculating the corrected QT interval (QTc).

An echocardiogram was performed as described previously^115,116^. Briefly, mice anaesthetized as in the electrocardiogram were placed in a supine position on a heated (to 37 °C) ultrasound platform to maintain body temperature. The paw of the mouse was attached to the sensor recording the electrocardiogram and the respiratory rate on a Vevo 2100 Ultrasound Imaging System (with MS 550D transducer, FUJIFILM VisualSonics). The abdominal hair was removed using depilatory cream, and the exposed skin was daubed with ultrasound gel. The ultrasound transducer was then placed on the chest over the heart. A B-mode tracing of the left ventricular (LV) endocardial border on the parasternal long axis was performed to assess the LV end-diastolic volume (LVEDV) and LV end-systolic volume (LVESV) using the Vevo 2100 software (v.1.6.0, FUJIFILM VisualSonics). LV ejection fraction (EF) was then calculated using the formula: EF = [(LVEDV − LVESV)/LVEDV] × 100.

### Determination of body temperature

Mouse body temperature was monitored by a FLIR A5 camera (Teledyne FLIR) as described previously^115^. Briefly, the mouse was gavaged with aldometanib or vehicle control, followed by placement in a customized tail-first restrainer with its scruff and back exposed to the camera. Data were recorded for 2 h at 1-min intervals, followed by another 6 h at 30-min intervals. Data were processed by BM IR software (v7.3.0.28, Teledyne FLIR) during which the BAT temperature (the interscapular BAT depot temperature) and the non-BAT temperature (temperature of other regions on the mouse back, regarded as normal surface temperature) were calculated.

The rectal temperature was measured using a thermal probe (daubed with Vaseline) connected to a Type K Digital Thermometer (421501, Extech Instruments).

### Determination of body composition

Lean and fat body mass were measured by quantitative magnetic resonance (EchoMRI -100H Analyzer; Echo Medical Systems) according to the manufacturer’s instructions. Briefly, the system was calibrated with oil standard before the measurement. Mice were individually weighed and inserted into a restrainer tube, and were immobilized by gently inserting a plunger. The mouse was then positioned to a gesture that curled up like a donut, with its head against the end of the tube. Body composition of each mouse was measured with two repeated runs, and the average values were taken for further analysis.

### Determination of energy expenditure

Mouse EE was determined by a metabolic cage system (Promethion Line, CAB-16-1-EU; Sable Systems International) as described previously^117^. Briefly, the system was maintained in a condition identical to that for housing mice (as described in ‘Aldometanib treatment in mice and rats’). Each metabolic cage in the 16-cage system consisted of a cage with standard bedding, a food hopper and water bottle, connected to load cells for continuous monitoring. To minimize the stress of a new environment, mice were acclimatized (by individually housing in the gas-calibrated chamber) for 1 week before data collection. Mice treated with vehicle or aldometanib were randomly assigned/housed to prevent systematic errors in measurement. Body weights and fat proportion of mice were determined before and after the acclimation and the food and water intake daily. Mice found not acclimatized to the metabolic cage (for example, resistance to eating and drinking) were removed from the study. Data acquisition (5-min intervals for each cage) and instrument control were performed using MetaScreen software (v.2.3.15.12, Sable Systems) and raw data were processed using Macro Interpreter (v.2.32, Sable Systems). Ambulatory activity and position were monitored using XYZ beam arrays with a beam spacing of 0.25 cm (beam breaks), and the mouse pedestrial locomotion (walking distance) within the cage was calculated accordingly. Respiratory gases were measured using the GA-3 gas analyser (Sable Systems) equipped with a pull-mode, negative-pressure system. Air flow was measured and controlled by FR-8 (Sable Systems), with a set flow rate of 2,000 ml min^−1^. Oxygen consumption (VO_2_) and carbon dioxide production (VCO_2_) were reported in ml per minute. Water vapour was measured continuously and its dilution effect on O_2_ and CO_2_ was compensated mathematically in the analysis stream. EE was calculated using the Weir equation: kcal h^−1^ = 60 × (0.003941 × VO_2_ + 0.001106 × VCO_2_. Differences of average EE were analysed by ANCOVA using body weight as the covariate. The RQ was calculated as VCO_2_/VO_2_.

### *Caenorhabditis elegans* strains

Nematodes (hermaphrodites) were maintained on NGM plates spread with *Escherichia coli* OP50 as standard food. All worms were cultured at 20 °C. Wild-type (N2 Bristol) and *aak*-*2*(*ok524*) strains were obtained from *Caenorhabditis* Genetics Center, and the *aak*-*2*(*ok524*) strain was outcrossed six times to N2 before the experiments. The *axl*-*1*, *lmtr*-*2*, HLH-30::GFP and *hsp-6*_*p*_*::gfp* strains that have been outcrossed six times to N2, as described previously^118^, were gifts from Y. Liu from the Institute of Molecular Medicine, Peking University. Unless stated otherwise, nematodes at the L4 stage were used.

### Evaluation of nematode lifespan and healthspan

To determine the lifespan of nematodes, the growth of nematodes was first synchronized: worms were washed off from agar plates with 15 ml of M9 buffer (22.1 mM KH_2_PO_4_, 46.9 mM Na_2_HPO_4_, 85.5 mM NaCl and 1 mM MgSO_4_) supplemented with 0.05% (vol/vol) Triton X-100 per plate, followed by centrifugation at 1,000*g* for 2 min. The worm sediment was suspended with 6 ml of M9 buffer containing 50% synchronizing bleaching solution (by mixing 25 ml of NaClO solution (5% active chlorine), 8.3 ml of 25% (wt/vol) NaOH, and 66.7 ml of M9 buffer, for a total of 100 ml), followed by vigorous shaking for 2 min, and centrifugation for 2 min at 1,000*g*. The sediment was washed with 12 ml of M9 buffer twice, then suspended with 6 ml of M9 buffer, followed by rotating at 20 °C, 30 r.p.m. for 12 h. Synchronized worms were cultured to L4 stage before transfer to desired agar plates (containing aldometanib or not) for determining lifespan. Worms were transferred to new plates every 2 d. Live and dead worms were counted during the transfer. Worms that displayed no movement after gentle touching with a platinum picker were judged as dead. Kaplan–Meier curves were graphed in Prism 9 (GraphPad Software) and the statistical analysis data in SPSS 27.0 (IBM).

Pharyngeal pumping rates, assessed as the numbers of contraction–relaxation cycles of the terminal bulb on nematode pharynx within 1 min, were determined as described previously^119^, with minor modifications. Briefly, the synchronized nematodes were cultured to the L4 stage, and aldometanib was administered thereafter. The 1-day-old nematodes were then picked and placed on a new NGM plate containing *E*. *coli*. After 10 min of incubation at room temperature, the contraction–relaxation cycles of the terminal bulb of each worm were recorded on a stereomicroscope (M165 FC, Leica) through a ×63 objective for a consecutive 4 min using the Capture software (v.2021.1.13, Capture Visualisation), and the average contraction–relaxation cycles per min were calculated using the Aimersoft Video Editor software (v.3.6.2.0, Aimersoft).

Brood size was determined as described previously^120^. Briefly, synchronized nematodes were singly cultured in plates at the L4 stage, after which aldometanib was administered. Throughout the assay, the adult worms were transferred to a new plate every day and the total numbers of eggs and young larvae were recorded.

The resistance of nematodes to the oxidative stress was determined as described previously^106^. Briefly, synchronized worms were cultured to L4 stage after which aldometanib was administered. After 1 day of aldometanib treatment, 20 worms were transferred to an NGM plate containing 15 mM ferrous sulfate. Worms were then cultured at 20 °C on such a plate, during which the live and dead worms were counted at every hour.

### Reagents

Rabbit anti-phospho-AMPKα-T172 (2535; 1:1,000 dilution for IB), anti-AMPKα (2532; 1:1,000 dilution for IB), anti-phospho-ACC-Ser79 (3661; 1:1,000 dilution for IB), anti-ACC (3662; 1:1,000 dilution for IB), anti-ALDOA (8060; 1:2,000 dilution for IB), anti-AXIN1 (2074; 1:1,000 dilution for IB), anti-LAMTOR1 (8975; 1:500 dilution for IB), anti-phospho-p70 S6K-S389 (9234; 1:1,000 dilution for IB), anti-p70 S6K (2708; 1:1,000 dilution for IB), anti-phospho-SREBP1c-S372 (9874; 1:500 dilution for IB), anti-phospho-TBC1D1-S660 (6928; 1:500 dilution for IB), anti-TBC1D1 (4629; 1:1,000 dilution for IB), anti-ATG5 (12994; 1:1,000 dilution for IB), anti-p62 (23214; 1:1,000 dilution for IB), anti-SDHA (11998; 1:1,000 dilution for IB) and anti-COX4 (4850; 1:1,000 dilution for IB) antibodies were purchased from Cell Signaling Technology. Rabbit anti-transferrin (ab1223; 1:500 dilution for IB), anti-ATP6V1B2 (ab73404; 1:2,000 dilution for IB), anti-UCP1 (ab10983; 1:1,000 dilution for IB), anti-VDAC1 (ab34726; 1:1,000 dilution for IB), anti-LONP1 (ab103809; 1:1,000 dilution for IB), anti-F4/80 (ab111101; 1:250 dilution for IHC), mouse anti-total oxidative phosphorylation (OXPHOS) complex (ab110413; 1:1,000 dilution for IB homogenates of MEFs, and 1:5,000 dilution for adipose tissues), rat anti-LAMP2 (ab13524; 1:1,000 dilution for IB or 1:120 for IF) and horseradish peroxidase (HRP)-conjugated goat anti-Rat IgG (ab7097; 1:2,000 dilution for IB) antibodies were purchased from Abcam. Rabbit anti-ALDOB (18065-1-AP; 1:1,000 dilution for IB), anti-HSP60 (15282-1-AP; 1:1,000 dilution for IB), and anti-tubulin (10068-1-AP, 1:1,000 dilution for IB nematode tubulin; and 66031-1-Ig, 1:20,000 dilution for IB mammalian tubulin) antibodies were purchased from Proteintech. Goat anti-AXIN (sc-8567; 1:100 dilution for immunoprecipitation (IP) and 1:120 dilution for IF), rabbit anti-SREBP1 (sc-366; 1:1,000 dilution for IB), mouse anti-HA (sc-7392; 1:1,000 dilution for IB) and mouse anti-goat IgG-HRP (sc-2354; 1:5,000 dilution for IB) antibodies were purchased from Santa Cruz Biotechnology. Mouse anti-ALDOC (AM2215b; 1:2,000 dilution for IB) antibody was purchased from Abgent. Normal rabbit control IgG (CR1; 1:100 dilution for IP) was purchased from Sino Biological. The HRP-conjugated goat anti-mouse IgG (115-035-003; 1:5,000 dilution for IB) and goat anti-rabbit IgG (111-035-003; 1:5,000 dilution for IB and 1:120 dilution for IHC) antibodies were purchased from Jackson ImmunoResearch. Alexa Fluor 488 donkey anti-goat IgG (A11055; 1:100 dilution for IF), Alexa Fluor 594 donkey anti-rat IgG (A21209; 1:100 dilution for IF) and Alexa Fluor 488 goat anti-rabbit IgG (A11008; 1:100 dilution for IHC) antibodies were purchased from Thermo Fisher. See validation information for antibodies in the Reporting Summary.

DMSO (D2650), methanol (646377), ethanol (459836), isopropanol (34863), chloroform (C7559), dichloromethane (650463), glucose (G7021), 2-DG (D8375), NaCl (S7653), KCl (P9333), CaCl_2_ (C5670), MgSO_4_ (M2643), H_2_O_2_ (H1009), KH_2_PO_4_ (P5655), K_2_HPO_4_ (P9666), NaH_2_PO_4_ (S3014), Na_2_HPO_4_ (S7907), NaOH (S8045), H_2_SO_4_ (339741), Na_2_SO_4_ (239313), NaBH_4_ (480886), KOH (484016), KI (207969), NH_4_Cl (A4514), MgCl_2_ (M8266), CaCO_3_ (C4830), CsCl (289329), SDS (436143), HEPES (H4034), MES (69889), streptomycin (85886), cholesterol (C3045), insulin (I1882), isopropyl-ß-d-thiogalactopyranoside (I6758), paraformaldehyde (158127), glycine (G8898), taurine (T8691), glycerol (G5516), NaHCO_3_ (S5761), EDTA (E6758), EGTA (E3889), sucrose (S7903), ATP (A6419), ADP (01897), phosphocreatine (V900832), formic acid (5.43804), acetic acid (27225), ammonium formate (70221), ammonium acetate (73594), ammonium sulfate (A4418), acetonitrile (34888), ethanolamine (411000), methoxyamine hydrochloride (89803), MTBSTFA (with 1% t-BDMCS; M-108), hexane (34859), pyridine (270970), imidazole (792527), sodium pyrophosphate (P8135), β-glycerophosphate (50020), AICAR (A9978), oligomycin A (75351), FCCP (C2920), antimycin A (A8674), rotenone (R8875), NADH (N8129), dithiothreitol (DTT; D9779), dithioerythritol (D8161), TMPD (T3134), lactobionate (L3375), l-glutamic acid (G1251), l-ascorbic acid (A5960), l-malic acid (M7397), succinic acid (S9512), sodium azide (S2002), formaldehyde solution (formalin; F8775), sodium hypochlorite solution (239305), PBS (P5493), xylene (534056), d-mannitol (M4125), sodium *tert*-butoxide (*t*-BuONa; 359270), THF (34865), 1-bromohexadecane (234451), 2-methylglyoxaline (2-methyl-1H-imidazole; M50850), saponin (S4521), octyl β-d-glucopyranoside (ODG; O8001), Triton X-100 (T9284), IGEPAL CA-630 (NP-40; I3021), Tween-20 (P9416), Trizma base (Tris; T1503), TEA (V900257), poly-l-lysine solution (P8920), leupeptin (L2884), OptiPrep (D1556), Percoll (P4937), Kolliphor HS 15 (42966), polybrene (H9268), tyloxapol (T0307), cytochalasin D (C2618), FITC-dextran (90718), sodium iodoacetate (I2512), hydrazine (309400), β-mercaptoethanol (M6250), ammonium persulfate (APS; A3678), tetramethylethylenediamine (TEMED; T9281), Coomassie Brilliant Blue R-250 (1.12553), diethylpyrocarbonate (DEPC)-treated water (693520), agar (A1296), haematoxylin solution (03971), eosin Y-solution (318906), ammonium hydroxide solution (338818), hydrochloric acid in ethanol (1.00327), Canada balsam (C1795), triosephosphate isomerase (TPI)/GPDH (G1881), rabbit aldolase (ammonium sulfate precipitated; A8811), trypsin (T1426), Non-fat-Dried Milk bovine (M7409), agarose (A9539), PAS Staining System Kit (395B), Glycogen Assay Kit (MAK016), Lysosome Isolation Kit (LYSISO1), Endoplasmic Reticulum Isolation Kit (ER0100), collagenase B (11088831001), dispase II (4942078001), (*N*-methyl-d3)-palmitoyl-l-carnitine (55107), myristic-d27 acid (68698), tridecanoic acid (91988), BSA (A2153) and fatty acid-free BSA (SRE0098) were purchased from Sigma. 2-(bromomethyl)-1,3-dichlorobenzene (D832438) was purchased from Macklin. Polyethylenimine (23966) was purchased from Polysciences. FBP (sc-214805) was purchased from Santa Cruz Biotechnology. Dynasore (S8047), Dyngo-4a (S7163) and nystatin (S1934) were purchased from Selleck. Methyl-β-cyclodextrin (HY-101461) was purchased from MedChemExpress. ReverTra Ace qPCR RT Master Mix with gDNA Remover (FSQ-301) was purchased from Toyobo. [3-^3^H]-glucose (NET331C001MC) was purchased from PerkinElmer. LabAssay triglyceride reagent (290-63701) was purchased from Wako Pure Chemical Industries. Bacteriological peptone (LP0037), yeast extract (LP0021) and tryptone (LP0042) were purchased from Oxoid. Internal Standards 1 (H3304-1002) and Internal Standards 3 (H3304-1104) were purchased from Human Metabolome Technologies. rProtein A Sepharose Fast Flow (17127904), Protein G Sepharose 4 Fast Flow (17061806), Superdex 200 Increase 10/300 GL (28990944), PBS-P (28995084), Series S Sensor Chip CM5 (BR100530) and Amine Coupling kit (with 1-ethyl-3-(3-dimethylaminopropyl)carbodiimide and *N*-hydroxysuccinimide (NHS) included; BR100050) were purchased from Cytiva. WesternBright ECL and Peroxide solutions (210414-73) were purchased from Advansta. Acrylamide/Bis Solution (30%), 29:1 (1610156) was purchased from Bio-Rad. Protease inhibitor cocktail (70221) was purchased from Roche. Seahorse XF base medium (103334) was purchased from Agilent. LysoSensor Green DND-189 (L7535), ProLong Diamond Antifade Mountant (P36970), ProLong Live Antifade Reagent (P36975), NeutrAvidin Agarose (29204), Lipofectamine 2000 (11668500), DMEM—high glucose (12800082), DMEM—no glucose (11966025), MEM non-essential amino acids solution (11140050), Maxima SYBR Green/ROX qPCR Master Mix (K0223), Trypan Blue Stain (T10282), FBS (10099141 C), penicillin–streptomycin (15140163), Ham’s F-10 medium (11550043), William’s E medium, no glutamine (12551032), Liver Perfusion Medium (17701), Liver Digest Medium (17703), GlutaMAX (35050061), sodium pyruvate (11360070), basic fibroblast growth factor (13256-029), EZ-Link Sulfo-NHS-SS-Biotin (21331), Prestained Protein MW Marker (26612) and TRIzol (15596018) were purchased from Thermo Scientific. Isoflurane was purchased from RWD Life Science. Mouse Ultrasensitive Insulin ELISA kit (80-INSMSU-E10) and Rat Ultrasensitive Insulin ELISA kit (80-INSRTU-E01) were purchased from ALPCO. Lactic acid assay kit (A019-2-1), ALT assay kit (C009-2-1), AST assay kit (C010-2-1) and ALP assay kit (A059-2-2) were purchased from Nanjing Jiancheng Bioengineering Institute. RNAprotect Tissue Reagent (76106) was purchased from Qiagen. Depilatory cream was purchased from Veet. Ultrasound gel (30809) was purchased from 3B Scientific. Paraplast High Melt Paraffin (39601095) was purchased from Leica. TAG (15:0/15:0/15:0; 26962) was purchased from Cayman. [U-^13^C]-glutamine (184161-19-1), [U-^13^C]-palmitic acid (PA; CLM-409) and [U-^13^C]-glucose (CLM-1396) were purchased from Cambridge Isotope Laboratories. Picro Sirius Red Stain kit (ab150681) and Hydroxyproline Assay kit (ab222941) were purchased from Abcam. DAPI Fluoromount-G mountant (0100-20) was purchased from SouthernBiotech. Apo-BrdU In Situ DNA Fragmentation Assay Kit (K401) was purchased from BioVision. Biospin Tissue Genomic DNA extraction Kit (BSC04M1) was purchased from BioFlux.

### Plasmids

Full-length cDNAs used in this study were obtained either by PCR using cDNA from MEFs, or by purchasing from Origene or Sino Biological. TRPV4-GCaMP6s were constructed as described previously^15^ (154150, Addgene). Mutations of ALDOA were performed by PCR-based site-directed mutagenesis using PrimeSTAR HS polymerase (R40A, Takara). Expression plasmids for various epitope-tagged proteins were constructed in the pcDNA3.3 vector for transfection (ectopically expressed in mammalian cells), in the pBOBI vector for lentivirus packaging (stable expression in mammalian cells), or in the pET-28a (bacterial expression) vectors. PCR products were verified by sequencing (Invitrogen, China). The lentivirus-based vector pLV-H1-EF1a-puro was used for expression of siRNA in MEFs and HEK293T cells. All plasmids were purified using the CsCl density gradient ultracentrifugation method.

### Primary hepatocytes and myocytes

Mouse primary hepatocytes were isolated with a modified two-step perfusion method using Liver Perfusion Media and Liver Digest Buffer. Before isolation of hepatocytes, male mice were first anaesthetized, followed by insertion of a 0.72 mm × 19 mm intravenous catheter into postcava. After cutting off the portal vein, mice were perfused with 50 ml of Liver Perfusion Media at a rate of 5 ml min^−1^, followed with 50 ml of Liver Digest Buffer at a rate of 2.5 ml min^−1^. The digested liver was then briefly rinsed by PBS, and then dismembered by gently tearing apart the Glisson’s capsule with two sterilized, needle-pointed tweezers on a 6-cm dish containing 3 ml of PBS. The dispersed cells were mixed with 10 ml of ice-cold William’s medium E plus 10% FBS, and were filtered by passing through a 100-μm cell strainer (BD Falcon). Cells were then centrifuged at 50*g* at 4 °C for 2 min, followed by washing twice with 10 ml of ice-cold William’s medium E plus 10% FBS. Cells were then immediately plated (at 60–70% confluence) in collagen-coated six-well plates in William’s medium E plus 10% FBS, 100 IU penicillin and 100 mg ml^−1^ streptomycin, and were maintained at 37 °C in a humidified incubator containing 5% CO_2_. After 4 h of attachment, the medium was replaced with fresh William’s medium E with 1% (wt/vol) BSA for another 12 h before further use.

Mouse primary myocytes were isolated as described previously^121^. Briefly, mice were killed by cervical dislocation, and hindlimb muscles from both legs were excised. Tissues were minced and digested in a collagenase B/dispase/CaCl_2_ solution for 1.5 h at 37 °C on a shaking bath. DMEM supplemented with 10% FBS was then added to the digested tissues, the mixtures were gently triturated, followed by loading onto a 70-μm strainer filter (BD Falcon). Cell suspensions were then centrifuged at 1,000*g* for 5 min, and the pellets were resuspended in growth medium (Ham’s F-10 medium supplemented with 20% FBS and 2.5 ng ml^−1^ basic fibroblast growth factor). Cells were then plated on collagen-coated dishes (354456, Corning) at 60–70% confluence.

### Cell lines

In this study, no cell line used is on the list of known misidentified cell lines maintained by the International Cell Line Authentication Committee (https://iclac.org/databases/cross-contaminations/). HEK293T cells and MEFs were maintained in DMEM (high glucose) supplemented with 3.7 g/l NaHCO_3_, 10% FBS, 100 IU penicillin and 100 mg ml^−1^ streptomycin at 37 °C in a humidified incubator containing 5% CO_2_. All cell lines were verified to be free of mycoplasma contamination and authenticated by STR sequencing. Polyethylenimine at a final concentration of 10 μM was used to transfect HEK293T cells. Total DNA to be transfected for each plate was adjusted to the same amount by using relevant empty vector. Transfected cells were collected 24 h after transfection.

Lentiviruses, including those for knockdown or stable expression, were packaged in HEK293T cells by transfection using Lipofectamine 2000. At 30 h after transfection, medium (DMEM supplemented with MEM non-essential amino acids; approximately 2 ml) was collected and centrifuged at 5,000*g* for 3 min at room temperature. The supernatant was mixed with 10 μg ml^−1^ (final concentration) polybrene, and was added to MEFs or HEK293T cells, followed by centrifuging at 3,000*g* for 30 min at room temperature (spinfection). Cells were incubated for another 24 h (MEFs) or 12 h (HEK293T cells) before further treatments.

*Lamtor1*^fl/fl^, *Axin*^fl/fl^, *Lkb1*^fl/fl^, *Camkk2*^−/−^ and *Atg5*^fl/fl^ MEFs were established by introducing SV40 T antigen via lentivirus into cultured primary embryonic cells from male mouse litters. *Lamtor1*^−/−^ MEFs were generated by infecting *Lamtor1*^fl/fl^ MEFs with adenoviruses expressing the Cre recombinase for 12 h, so do *AXIN*^−/−^, *LKB1*^−/−^ and *ATG5*^−/−^ MEFs. The infected cells were then incubated in fresh DMEM for another 12 h before further treatments. ALDO-TKD MEFs, TRPV-QKO MEFs, *PEN2*^−/−^ MEFs, *ATP6AP1*^−/−^ MEFs and si*ATP6v0c* HEK293T cells were generated as described previously^13–15,62^.

### Immunoprecipitation and immunoblotting

For determining the AMPK-activating complex formation, AXIN was immunoprecipitated and analysed as described previously^13^ with minor modifications. Briefly, four 15-cm dishes of MEFs (grown to 80% confluence) were collected for IP of AXIN. Cells were lysed with 750 μl per dish of ice-cold ODG buffer (50 mM Tris-HCl, pH 8.0, 50 mM NaCl, 1 mM EDTA, 2% (wt/vol) ODG, 5 mM β-mercaptoethanol with protease inhibitor cocktail), followed by sonication and centrifugation at 4 °C for 15 min. Cell lysates were incubated with anti-AXIN antibody overnight. Overnight protein aggregates were pre-cleared by centrifugation at 20,000*g* for 10 min, and protein A/G beads (1:250 dilution, balanced with ODG buffer) were then added into the lysate/antibody mixture for another 3 h at 4 °C. The beads were centrifuged and washed with 100 times the volume of ODG buffer three times (by centrifuging at 2,000*g*) at 4 °C and then mixed with an equal volume of 2× SDS sample buffer and boiled for 10 min before IB.

To analyse the levels of p-AMPKα and p-ACC in MEFs, HEK293T cells, primary hepatocytes and primary myocytes, cells grown to 70–80% (except for hepatocytes 60–70%) confluence in a well of a six-well dish were lysed with 250 μl of ice-cold lysis buffer (20 mM Tris-HCl, pH 7.5, 150 mM NaCl, 1 mM EDTA, 1 mM EGTA, 1% (vol/wt) Triton X-100, 2.5 mM sodium pyrophosphate, 1 mM β-glycerophosphate, with protease inhibitor cocktail). The lysates were then centrifuged at 20,000*g* for 10 min at 4 °C and an equal volume of 2× SDS sample buffer was added into the supernatant. Samples were then boiled for 10 min and then directly subjected to IB.

To analyse the levels of p-AMPKα and p-ACC in tissues, mice or rats were anaesthetized after indicated treatments. Freshly excised (or freeze-clamped) tissues were immediately lysed with ice-cold lysis buffer (10 μl per mg tissue weight for liver, heart, kidney and brain, and 5 μl per mg tissue weight for adipose tissue and muscle), followed by homogenization and centrifugation as described above. The lysates were then mixed with 2× SDS sample buffer, boiled, and samples subjected to IB. To analyse the levels of p-AMPKα and p-ACC in nematodes, about 150 nematodes cultured on the NGM plate were collected for each sample. Worms were quickly washed with ice-cold M9 buffer containing Triton X-100, and were lysed with 150 μl of ice-cold lysis buffer. The lysates were then mixed with 5× SDS sample buffer, followed by homogenization and centrifugation as described above, and then boiled before being subjected to IB. To analyse the protein levels of mitochondrial OXPHOS complexes in adipose tissues, mice were anaesthetized after indicated treatments. Freshly excised tissues were immediately lysed with ice-cold lysis buffer (10 μl per mg tissue weight for iWAT, and 40 μl per mg for BAT), followed by homogenization and centrifugation as described above. The lysates were then mixed with 2× SDS sample buffer, and subjected to IB. All samples were subjected to IB on the same day of preparation, and any freeze–thaw cycles were avoided.

For IB, the SDS–PAGE gels were prepared in-house. Briefly, the resolving gel solution (8%, 10 ml) was prepared by mixing 1.9 ml of 30% Acryl/Bis solution, 1 ml of 10× Lower Buffer (3.5 M Tris, 1% (wt/vol) SDS, pH 8.8), and 0.48 ml of 65% (wt/vol) sucrose (dissolved in water) with 6.62 ml of water; and the stacking gel solution (5 ml) was prepared by mixing 668 μl of 30% Acryl/Bis solution, and 1.25 ml of 4× Stacking Buffer (0.5 M Tris, 0.4% (wt/vol) SDS, pH 6.8) with 3.08 ml water. For each glass gel plate (with 1.0-mm spacer; 1653308 and 1653311, Bio-Rad), approximately 7 ml of resolving gel solution and 2.5 ml of stacking gel solution were required. APS (to 0.1% (wt/vol) final concentration) and TEMED (to 0.1% (vol/vol) final concentration) were added to the resolving gel solution. The resolving gel was overlayed with 2 ml of 75% (vol/vol) ethanol before the acrylamide polymerization. After around 20 min (when a clear line between resolving gel and ethanol is seen), the overlaid ethanol was poured off, dried by filter paper, and followed by placing at room temperature for another 15 min to let ethanol evaporate completely. The gel cassette was then filled with APS/TEMED-supplemented stacking gel solution, followed by placing a 15-well comb into the cassette, and then placed it at room temperature for 20 min. After removing the comb, the gel was rinsed with Running Buffer (25 mM Tris, 192 mM glycine, 1% (wt/vol) SDS, pH 8.3) before sample loading. Samples of less than 10 μl were loaded into wells, and the electrophoresis was run at 100 V by a Mini-PROTEAN Tetra Electrophoresis Cell (Bio-Rad). In this study, all samples were resolved on 8% resolving gels, except those of UCP1 and VDAC, which were prepared as those of 8%, except that a final concentration of 10% Acryl/Bis was added to the resolving gel solution, and those of LAMTOR1, COX4 and total OXPHOS 15%. To transfer the resolved proteins, the pre-cut PVDF membrane (0.45 μm; IPVH00010, Merck) was incubated in methanol for 1 min, followed by equilibrating and soaking in pre-cooled Transfer Buffer (25 mM Tris, 192 mM glycine, 10% (vol/vol) methanol) for more than 5 min. After preparing the gel/membrane sandwich, the transfer was performed at a voltage at 100 V in a Mini Trans-Blot Cell (Bio-Rad) for 1 h at 4 °C. The blotted PVDF membrane was then incubated in blocking buffer (5% (wt/vol) BSA or 5% (wt/vol) non-fat milk (according to the instructions from the antibody suppliers) dissolved in TBST, which comprises 40 mM Tris, 275 μM NaCl, 0.2% (vol/vol) Tween-20, pH 7.6) for another 2 h on an orbital shaker at room temperature, followed by rinsing with TBST for twice, 5 min each. The PVDF membrane was incubated with the desired primary antibody overnight at 4 °C on an orbital shaker with gentle shaking, followed by rinsing with TBST three times, 5 min each at room temperature, and then the membrane was incubated with the secondary antibody for 3 h at room temperature with gentle shaking. The secondary antibody was then removed, and the PVDF membrane was further washed with TBST for three times, 5 min each at room temperature. PVDF membranes were incubated in ECL mixture (by mixing equal volumes of ECL solution and peroxide solution for 5 min), then each membrane was placed onto a plastic wrap and laid with Medical X-Ray Film (FUJIFILM) in a light-proof cassette for a desired period of time. The films were then developed with X-OMAT MX Developer and Replenisher and X-OMAT MX Fixer and Replenisher solutions (Carestream) on a Medical X-Ray Processor (Carestream) using Developer (Model 002, Carestream). The developed films were scanned using a Perfection V850 Pro scanner (Epson) using Epson Scan software (v.3.9.3.4), and were cropped using Photoshop 2022 (Adobe). Levels of total proteins and phosphorylated proteins were analysed on separate gels, and representative immunoblots are shown. The band intensities on developed films were quantified using ImageJ (v.1.8.0, National Institutes of Health Freeware).

### Quantification of mRNA levels of mitochondrial genes

Mice treated with aldometanib were killed by cervical dislocation, immediately followed by dissecting the gastrocnemius muscle. The muscle tissue was roughly sliced to cubes with edge lengths of approximately 2 mm, and then soaked in RNAprotect Tissue Reagent (1 ml per 100 mg of tissue) for 24 h at room temperature. The tissue was then incubated in 1 ml of TRIzol, followed by three rounds of freeze–thaw cycles, and was then homogenized. The homogenate was centrifuged at 12,000*g* for 15 min at 4 °C, and 900 µl of clear supernatant (not the lipid layer on the top) was transferred to an RNase-free tube. The supernatant was then added with 200 µl of chloroform, followed by vigorous vortexing for 15 s. After centrifugation at 12,000*g* for 15 min at 4 °C, 450 µl of the upper aqueous layer was transferred to an RNase-free tube. The RNA was then precipitated by adding 450 µl of isopropanol, followed with centrifugation at 12,000*g* for 30 min at 4 °C. The pellet was washed twice with 75% ethanol, and once with 100% ethanol, and was dissolved with 20 µl of DEPC-treated water. The concentration of RNA was determined by a NanoDrop 2000 spectrophotometer (Thermo Fisher). A total of 1 µg of RNA was diluted with DEPC-treated water to a final volume of 10 µl, heated at 65 °C for 5 min, and chilled on ice immediately. The Random Primer Mix, Enzyme Mix and 5× RT buffer (all from the ReverTra Ace qPCR RT Master Mix) were then added to the RNA solution, followed by incubation at 37 °C for 15 min, and then at 98 °C for 5 min on a thermocycler. The reverse-transcribed cDNA was quantified with Maxima SYBR Green/ROX qPCR Master Mix on a LightCycler 480 II System (Roche) with the following programmes: pre-denaturing at 95 °C for 10 min; denaturing at 95 °C for 10 s, then annealing and extending at 65 °C for 30 s in each cycle (determined according to the amplification curves, melting curves, and bands on agarose gel of serial pilot reactions (in which a serial annealing temperature was set according to the estimated annealing temperature of each primer pair) of each primer pair, and the same hereafter), for a total of 45 cycles. Primer pairs for mouse *Nd1*, *Nd2*, *Nd3*, *Nd4*, *Nd4l*, *Nd5*, *Nd6*, *Ndufab1*, *Cytb*, *Uqcrc1*, *Uqcrc2*, *Atp5f1b* (*Atp5b*), *Cox6a1*, *Atp6*, *Atp8*, *Cox1* and *Cox3* were generated as described previously^85^, and others using the Primer-BLAST website (https://www.ncbi.nlm.nih.gov/tools/primer-blast/index.cgi; see the complete DNA primers list in Supplementary Table 3). The mRNA level was then calculated using the comparative ΔΔct method using the LightCycler software (v.96 1.1, Roche; same hereafter).

Nematodes at L4 stage treated with aldometanib for 1 d were used for analysis of mitochondrial gene expression. Some 1,000 worms were collected with 15 ml of M9 buffer containing 0.05% Triton X-100 (vol/vol), followed by centrifugation for 2 min at 1,000*g*. The sediment was then washed with 1 ml of M9 buffer twice, and then lysed with 1 ml of TRIzol. Worms were then frozen in liquid nitrogen, thawed at room temperature, and then the freeze–thaw cycle was repeated another two times. The worm lysates were then placed at room temperature for 5 min, then mixed with 0.2 ml of chloroform, followed by vigorous shaking for 15 s. RNA extraction and quantification were performed as for muscle tissues. Primer pairs used for qPCR are as previously described^122,123^, except those of *C*. *elegans ctb-1* were designed using the Primer-BLAST website (Supplementary Table 3).

### Analysis of mitochondrial DNA copy numbers in mice and *C*. *elegans*

Mouse tissue DNA was extracted with the Biospin tissue genomic DNA extraction kit (BioFlux) following the manufacturer’s instruction, with minor modifications. Briefly, mice treated with aldometanib were killed by cervical dislocation, quickly followed by dissecting the gastrocnemius muscle. The muscle tissue was then grinded on a ceramic mortar in liquid nitrogen. Next, 50 mg of grinded tissue was then transferred to a 1.5-ml microcentrifuge tube, followed by addition of 600 µl of FL buffer and 10 µl of PK solution containing 2 µl of 100 mg ml^−1^ RNase A. The mixture was then incubated at 56 °C for 15 min, followed by centrifuging at 12,000*g* for 3 min. Then, 500 µl of supernatant was transferred to a 2-ml microcentrifuge tube, followed by mixing with 700 µl of binding buffer and 300 µl of absolute ethanol. The mixture was then loaded onto a Spin column, and was centrifuged at 10,000*g* for 1 min. The flow-through was discarded, and 500 µl of the PW buffer was added to the Spin column, followed by centrifuging at 10,000*g* for 30 s. Some 600 µl of washing buffer was then added to the spin column, followed by centrifuging at 10,000*g* for 30 s, and then repeated once. The Spin column was then centrifuged for 1 min at 10,000*g* to completely remove the washing buffer, and the DNA on the column was eluted by 100 µl of elution buffer (added to Spin column, followed by incubation at room temperature for 5 min, and then centrifuged at 12,000*g* for 1 min). Total DNA was quantified with Maxima SYBR Green/ROX qPCR Master Mix on a LightCycler 480 II System (Roche) with the following programmes: 70 ng of DNA was pre-denatured at 95 °C for 10 min, and then subjected to PCR for a total of 45 cycles; denaturing at 95 °C for 10 s, annealing and extending at 65 °C for 30 s in each cycle. Primer pairs used for qPCR are as previously described^124^ (see the complete DNA primers list in Supplementary Table 4). The mtDNA:nDNA ratio was then calculated using the comparative ΔΔct method.

Nematode DNA copy numbers were determined from worm lysates. Briefly, 30 synchronized early L4 worms were collected, and were lysed with 10 μl of worm lysis buffer (50 mM HEPES, pH 7.4, 1 mM EGTA, 1 mM MgCl_2_, 100 mM KCl, 10% (vol/vol) glycerol, 0.05% (vol/vol) NP-40, 0.5 mM DTT and protease inhibitor cocktail). The worm lysate was frozen at −80 °C overnight, followed by incubating at 65 °C for 1 h and 95 °C for 15 min. Nematode DNA was then quantified with Maxima SYBR Green/ROX qPCR Master Mix on a LightCycler 480 II System (Roche) with the following programmes: pre-denaturing at 95 °C for 10 min and then for a total of 45 cycles of denaturing at 95 °C for 10 s, and annealing and extending at 65 °C for 30 s in each cycle. Primer pairs used for qPCR are designed as described previously^107^ (Supplementary Table 4). The mtDNA:nDNA ratio was then calculated using the comparative ΔΔct method.

### Quantification of mRNA levels of inflammation-related and fibrogenesis-related genes

NASH mice treated with vehicle or aldostatin were killed by cervical dislocation, immediately followed by dissection of the liver. The mRNA of liver tissue was extracted and reverse transcribed to cDNA in the same way as that of muscle, except that 100 µl of DEPC-treated water was used to dissolve mRNA. The reverse-transcribed cDNA was quantified as with the muscle tissue, and the primer pairs are designed as described previously^78,125^ (see the complete DNA primers list in Supplementary Table 5). The mRNA level was then calculated using the comparative ΔΔct method using the LightCycler software (v.96 1.1, Roche; same hereafter).

### Microscopy

For determining the lysosomal localization of AXIN, cells grown to 80% confluence on coverslips in six-well dishes were fixed for 20 min with 4% (vol/vol) formaldehyde in PBS at room temperature. The coverslips were rinsed twice with PBS and permeabilized with 0.1% (vol/vol) Triton X-100 in PBS for 5 min at 4 °C. After rinsing twice with PBS, the coverslips were incubated with anti-AXIN and anti-LAMP2 (both 1:100, diluted in PBS) overnight at 4 °C. The cells were then rinsed three times with 1 ml of PBS, and then incubated with secondary antibodies (Alexa Fluor 488 donkey anti-goat IgG and Alexa Fluor 594 donkey anti-rat IgG) for 8 h at room temperature in the dark. The coverslips were washed for another four times with 1 ml of PBS, and then mounted on slides using ProLong Diamond Antifade Mountant. Confocal microscopic images were taken using an LSM 780 with a ×63 1.4-NA oil objective using ZEN 2012 software.

For detecting the pH of lysosomes, cells were grown on 35-mm glass-bottom dishes (D35-20-1-N, Cellvis), and were cultured to 60–80% confluence. Cells were treated with 1 μM (final concentration) LysoSensor Green DND-189 for 1 h, then washed twice with PBS and incubated in fresh medium for another 30 min. In the meantime, ProLong Live Antifade Reagent was added into the medium for staining nuclei before taking images. MEFs expressing GCaMP6s-fused TRPV protein were directly imaged after adding ProLong Live Antifade Reagent to the medium. During imaging, live cells were kept at 37 °C, 5% CO_2_ in a humidified incubation chamber (Incubator PM S1, Zeiss). Images were taken using an LSM 780 with a ×63 1.4-NA oil objective using ZEN 2012.

For detecting nuclear translocation of GFP-tagged HLH-30 expressed in nematodes, synchronized nematodes cultured to the L4 stage were treated with aldometanib for 24 h. Nematodes were then placed on an injection pad prepared by placing two drops (approximately 50 μl) of boiling 4% agarose (wt/vol) onto the centre of a glass coverslip (24 × 50 mm, 0.13–0.15 mm thickness), immediately followed by flattening with another coverslip, then dried at room temperature for 24 h. Images were taken with an LSM 900 with a ×20, 0.8-NA plan-Apochromat air objective by ZEN 3.1 software (Zeiss).

For detecting expression levels of HSP-6 in nematodes, the zcIs13[*hsp-6p::gfp*] strain, as a reporter, was used. Synchronized nematodes cultured to the L4 stage were treated with aldostatin for 24 h. Nematodes were incubated in M9 buffer containing 100 nM NaN_3_ for 15 min at room temperature, and then placed on a 4% agarose (wt/vol) injection pad and visualized on a Leica DM4 B.

For taking images using an LSM 780, LysoSensor, GCaMP6s and Alexa 488 dyes were visualized with an Ar gas laser (laser module LGK 7812) at 488 nm; Alexa 594 was visualized with a HeNe gas laser (LGK 7512 PF) at 594 nm. When images were taken using the LSM 900, GFP was excited with laser module URGB (400102-9301-000, Toptica) using a 10-mW laser line. The parameters, including ‘PMT voltage’, ‘offset’, ‘pinhole’ and ‘gain’, were kept unchanged between each picture taken. The resolution of image was 1,024 × 1,024 pixels. Images were processed and analysed on ZEN 2012 (for LSM 780) or ZEN 3.1 (for LSM 900), and formatted on Photoshop 2022 (Adobe).

### Determination of endocytosis in aldometanib-treated cells

The effects of aldometanib on endocytosis were determined by a flow cytometry-based method as described previously^126,127^. Briefly, MEFs grown to 70–80% confluence in a well of a six-well dish were incubated in 2 ml of DMEM containing 1 mg ml^−1^ (final concentration) FITC-dextran for 2 or 4 h at 37 °C in a humidified incubator containing 5% CO_2_. Cells were then washed with 3 ml of DMEM (preheated to 37 °C) three times, and then trypsinized. Next, 1 × 10^6^ of trypsinized cells were resuspended with 0.5 ml of PBS supplemented with 2% (mass/vol) BSA, and were filtered by passing through a 100-μm cell strainer. Flow cytometry was performed on an LSRFortessa Cell Analyzer (BD Biosciences), with the 488-nm (50 mW) laser and the 530/30 filter used to excite and detect the fluorescence of FITC-dextran. Detector voltages were optimized using a modified voltage titration approach^128^ and were standardized from day to day using the median fluorescence intensity target values and six-peak Ultra Rainbow Beads^129^ (Spherotec, URCP-38-2K). Gating strategies, including the FSC-SSC gate, used for analytical samples are shown in Extended Data Fig. 3n. Gate boundaries either were set based on control samples, or followed density distributions based on best practices. Data were collected by the FACSDiva software (v8.0.2, BD Biosciences) and were exported in the FCS 3.1 format for analysis by FlowJo software (v10.6.x, BD Biosciences). During the analysis, a combination of manual gating and computational analysis approaches^130^, with doublets being excluded by FSC-A versus FSC-H gating, was used.

### Measurement of oxygen consumption rates

For measuring OCR in MEFs, cells were plated at 10,000 cells per well on a 96-well Seahorse XF Cell Culture Microplate (Agilent) in full medium (DMEM containing 10% FBS) overnight before the experiment. Medium was then changed to Seahorse XF Base Medium supplemented with 25 mM glucose, 4 mM glutamine (GlutaMAX) and 1 mM pyruvate 1 h before the experiment. Cells were then placed in a CO_2_-free, XF96 Extracellular Flux Analyzer Prep Station (Agilent) at 37 °C for 1 h. OCR was then measured at 37 °C in an XF96 Extracellular Flux Analyzer (Agilent), with a Seahorse XFe96 sensor cartridge (Agilent) pre-equilibrated in Seahorse XF Calibrant solution (Seahorse Bioscience, Agilent) in a CO_2_-free incubator at 37 °C overnight. The assay was performed on a Seahorse XFe96 Analyzer (Agilent) at 37 °C following the manufacturer’s instruction. Concentrations of respiratory chain inhibitors used during the assay were: oligomycin at 10 μM, FCCP at 10 μM, antimycin A at 1 μM and rotenone at 1 μM (all final concentrations). Data were collected using Wave 2.6.1 Desktop software (Agilent) and exported to Prism 9 (GraphPad) for further analysis according to the manufacturer’s instructions.

The OCR of nematodes was measured as described previously^131^. Briefly, nematodes were washed with M9 buffer three times to remove *E*. *coli*. Around 15 to 25 nematodes were then suspended in 200 μl of M9 buffer, and were added to a well on a 96-well Seahorse XF Cell Culture Microplate. The measurement was performed as for MEFs, except that 10 μM FCCP and sodium azide (40 mM) were added to nematodes during the assay, and the temperature of Seahorse XFe96 Analyzer was set at 20 °C. Data were collected and analysed as in MEFs. At the end of the assay, the exact number of nematodes in each well was determined on a Cell Imaging Multi-Mode Reader (Cytation 1, BioTek) and was used for normalizing/correcting OCR results.

Muscular OCR was determined as described previously^132^. Briefly, the gastrocnemius muscle was dissected into thin fibre bundles and then immersed in ice-cold Isolation Solution A (10 mM Ca-EGTA buffer (2.77 mM CaK_2_EGTA and 7.23 mM K_2_EGTA), pH 7.1, 20 mM imidazole, 20 mM taurine, 49 mM K-MES, 3 mM K_2_HPO_4_, 9.5 mM MgCl_2_, 5.7 mM ATP, 15 mM phosphocreatine and 1 mM leupeptin) and was then permeabilized by addition of 50 μg ml^−1^ saponin by gently mixing at 4 °C for 10 min. The fibre bundles were then washed three times by Respiration Medium B (0.5 mM EGTA, 3 mM MgCl_2_.6H_2_O, pH 7.1, 20 mM taurine, 10 mM KH_2_P0_4_, 20 mM HEPES, 1 g l^−1^ BSA, 60 mM potassium-lactobionate, 110 mM mannitol and 0.3 mM DTT) before the assay. A total of 5 mg of muscle fibre bundles suspended in Respiration Medium B was transferred to an oxygraphy chamber on an Oxygraph-2k (Oroboros Instruments), followed by incubation for 5 min. Glutamate (final 10 mM) and malate (5 mM) were added to the chamber to determine the resting complex I-supported respiration (without ADP addition), followed by addition of 5 mM ADP to determine the maximal complex I-supported respiration. The complex I-supported respiration was then inhibited by addition of 1 μM rotenone, followed by addition of 10 mM succinate, to determine the complex II-supported respiration. The complex II-supported respiration was then inhibited by addition of 5 μM antimycin A, followed by addition of 0.5 mM TMPD and 2 mM ascorbate to determine the complex IV-supported respiration. Data were collected using DatLab software (v.7.3.0.3, Oroboros Instruments) and exported to Prism 9 for further analysis.

### Subcellular fractionation

Lysosomes were purified using the Lysosome Isolation Kit according to the manufacturer’s instructions, with minor modifications. Briefly, MEFs from 60 10-cm dishes (60–80% confluence), or 200 mg of mouse livers were collected by directly scraping at room temperature, followed by centrifugation for 5 min at 500*g* at 37 °C. Cells were resuspended in 7 ml of 1× Extraction Buffer containing protease inhibitor cocktail at room temperature, and were dounced in a 7-ml Dounce homogenizer (D9063, Sigma) for 120 strokes on ice followed by centrifugation for 10 min at 1,000*g*, at 4 °C, yielding post-nuclear supernatants (PNS). The PNS samples were then centrifuged for 20 min at 20,000*g* and the pellet was suspended by 1× Extraction Buffer by gentle pipetting, generating the crude lysosomal fraction (CLF). The volume of CLF was adjusted to 2.4 ml and then equally divided into six 1.5 ml microcentrifuge tubes (400 μl per tube). In total, 253 μl of OptiPrep and 137 μl of 1× OptiPrep dilution buffer were added to each CLF, and mixed by gentle pipetting. The mixture is defined as the diluted OptiPrep fraction. Each diluted OptiPrep fraction (0.8 ml) was loaded onto an 11 × 60-mm centrifuge tube at the top of 27% (0.4 ml) and 22.5% (0.5 ml) OptiPrep solution cushions, and then overlaid with 16% (1 ml), 12% (0.9 ml) and 8% (0.3 ml) OptiPrep solutions. The tube was then centrifuged on a SW60 Ti rotor (Beckman) at 150,000*g* for 4 h at 4 °C, and the fraction at the top of 12% OptiPrep solution was collected as the CLF. The fraction was diluted with two volumes of PBS, followed by centrifugation at 20,000*g* for 20 min. The supernatant was then aspirated, and the sediment was the lysosomal fraction.

Mitochondria were purified as described previously^133^, with minor modifications^9^. Briefly, 40 10-cm dishes of metformin-treated MEFs (60–80% confluence) were collected by scraping at room temperature, followed by centrifugation for 5 min at 500*g* at 37 °C. Cells were then resuspended in 20 ml of ice-cold IB_cells_-1 buffer (225 mM mannitol, 75 mM sucrose, 0.1 mM EGTA and 30 mM Tris-HCl, pH 7.4), and dounced for 100 strokes in a 40-ml Dounce homogenizer (D9188, Sigma), followed by centrifugation twice for 5 min at 600*g* at 4 °C. The supernatants were then collected and centrifuged for 10 min at 7,000*g* at 4 °C. The pellets were then washed twice with 20 ml of ice-cold IB_cells_-2 buffer (225 mM mannitol, 75 mM sucrose and 30 mM Tris-HCl, pH 7.4). The suspensions were centrifuged at 7,000*g*, and again at 10,000*g*, both for 10 min at 4 °C. The pellets were then resuspended in 2 ml of ice-cold MRB buffer (250 mM mannitol, 5 mM HEPES (pH 7.4) and 0.5 mM EGTA), and were loaded on top of 10 ml of Percoll medium (225 mM mannitol, 25 mM HEPES (pH 7.4), 1 mM EGTA and 30% Percoll (vol/vol)) in 14 × 89-mm centrifuge tubes (344059, Beckman). The tubes were then centrifuged on a SW41 rotor (Beckman) at 95,000*g* for 0.5 h at 4 °C, and the dense band located approximately at the bottom of each tube was collected. The collected fractions were diluted with 10 volumes of MRB buffer, followed by centrifugation at 6,300*g* for 10 min at 4 °C; the pellets were resuspended and washed with 2 ml of MRB buffer, followed with centrifugation at 6,300*g* for 10 min at 4 °C. The pellets contained pure mitochondria.

ER was purified according to the protocol optimized by combining the traditional microsome-based density gradient isolation method (Endoplasmic Reticulum Isolation Kit, Sigma) with the cell surface biotinylation reaction method (developed and optimized by Pierce), and was described previously^15^. Briefly, MEFs from 40 10-cm dishes (80% confluence) were quickly washed with ice-cold PBS (10 ml in each dish) twice, followed by incubating with 250 μg ml^−1^ of sulfo-NHS-SS-biotin (freshly dissolved in ice-cold PBS, 10 ml in each dish) for 30 min with gentle agitation on an orbital shaker at 4 °C. Approximately 500 μl of 1 M Tris (pH 8.0 at 4 °C) was then added to each dish to quench the biotinylation reaction. Cells were collected afterwards by directly scraping, followed by centrifugation at 600*g* for 5 min, and then washed with 40 ml of ice-cold PBS twice. Cells were then resuspended in 10 ml of 1× Hypotonic Extraction Buffer and then incubated at 4 °C for 30 min, with gentle mixing in the middle. Cells were then centrifuged at 600*g* at 4 °C for 5 min, and the pellet was resuspended with 6 ml of 1× Isotonic Extraction Buffer, followed by mixing in a 7-ml Dounce homogenizer for ten strokes. The homogenate was centrifuged at 1,000*g* for 10 min at 4 °C, and the PNS was further centrifuged at 12,000*g* for 15 min at 4 °C, yielding the supernatant as the post-mitochondrial fraction. The post-mitochondrial fraction was loaded in two 11 × 60-mm centrifuge tubes and then centrifuged on an SW60 Ti rotor (Beckman) at 100,000*g* for 1 h at 4 °C. The pellet was resuspended with 0.5 ml of 1× Isotonic Extraction Buffer, and was mixed in a 2-ml Dounce homogenizer for 20 strokes, yielding the microsomal suspension. The suspension was mixed with 0.25 ml of OptiPrep, and was carefully layered on the top of 1 ml of 30% OptiPrep solution (by mixing 0.5 ml of OptiPrep with 0.5 ml of 1× Isotonic Extraction Buffer) in an 11 × 60-mm centrifuge tube. Then, 2 ml of 15% OptiPrep solution (0.5 ml of OptiPrep mixed with 1.5 ml of 1× Isotonic Extraction Buffer) was then carefully layered on the top of the sample. The tube was then centrifuged on an SW60 Ti rotor at 150,000*g* for 3 h at 4 °C. The top 0.6 ml of 15% OptiPrep solution was discarded, and the following 200 μl of fraction was collected as the crude ER fraction. The fraction was then incubated with 100 μl of NeutrAvidin Agarose (pre-balanced by 1× Isotonic Extraction Buffer) for another 2 h. The supernatant was the ER fraction.

Cytosol was purified as described previously^134^. Briefly, ten 10-cm dishes of cells were homogenized in 800 μl of the homogenization buffer (HB; containing 250 mM sucrose, 3 mM imidazole, pH 7.4). Homogenates were then passed through a 22-gauge needle attached to a 1-ml syringe for a total of six times, and were then centrifuged at 2,000*g* for 10 min to yield PNS. PNS samples were then loaded onto the top of 11 × 60-mm centrifuge tubes that were loaded sequentially with 1 ml of 40.6% sucrose (dissolved in HB), 1 ml of 35% sucrose (dissolved in HB) and 1 ml of 25% sucrose (dissolved in HB). Tubes were then centrifuged on an SW60 Ti rotor (Beckman) at 35,000 r.p.m. for 1 h at 4 °C, and the top fractions (about 200 μl) were collected as the cytosolic fraction.

### Protein purification

To purify recombinant aldolase, full-length cDNAs encoding ALDOA, ALDOB and ALDOC were cloned into pET-28a (Novagen) vectors, and transformed into the *E*. *coli* strain BL21 (DE3). The transformed cells were induced with 0.1 mM isopropyl-ß-d-thiogalactopyranoside at an OD_600_ of 1.0. After incubating for another 12 h at 16 °C, the cells were collected and homogenized in an ice-cold His binding buffer (50 mM sodium phosphate, pH 7.4, 150 mM NaCl, 1% Triton X-100, 5% glycerol and 10 mM imidazole). The homogenates were then sonicated, and were subjected to ultracentrifugation at 150,000*g* for 30 min at 4 °C, followed by purification with Nickel Affinity Gel. The Nickel Affinity Gel was then washed with 100 times the volume of ice-cold His wash buffer (50 mM sodium phosphate (pH 7.4), 150 mM NaCl and 20 mM imidazole). Aldolase was eluted from the gel by ice-cold His elution buffer (50 mM sodium phosphate (pH 7.4), 150 mM NaCl and 250 mM imidazole). Proteins were concentrated to approximately 3 mg ml^−1^ by ultrafiltration (Millipore, UFC905096) at 4 °C, then subjected to a gel filtration column (Cytiva, Superdex 200) balanced with a buffer containing 50 mM Tris-HCl (pH 7.4) and 150 mM NaCl.

To purify endogenous aldolase from lysosomal and cytosolic fractions, lysosomes purified from 480 10-cm dishes and cytosol from 10 10-cm dishes were used. The procedures were as previously described^135^, with minor modifications. Briefly, the purified lysosomes were lysed with 20 ml of ice-cold lysis buffer, and cytosol diluted with ice-cold HB to 20 ml, followed by centrifugation at 20,000 *g* for 15 min at 4 °C. The supernatants were mixed with 5.56 g of ammonium sulfate (to 45% saturation) with continuous stirring at 4 °C for 1 h. The suspensions were centrifuged at 20,000*g* for 15 min at 4 °C, and the supernatants were mixed with 1.96 g of ammonium sulfate (to 60% saturation) with continuous stirring at 4 °C, followed by adjusting the pH to 7.4 with ammonium hydroxide. The suspensions were then incubated at 4 °C with continuous stirring for another 2 h, followed by centrifugation at 20,000*g* for 15 min. The pellets were dissolved in 2 ml of 10 mM Tris-HCl (pH 7.4) and 1 mM EDTA, and then subjected to Superdex 200 column balanced with a buffer containing 10 mM Tris-HCl (pH 7.4) and 1 mM EDTA.

### Enzymatic activity

Enzymatic activity of aldolase was determined using FBP as a substrate through an aldolase/TPI/GPDH-coupled assay system as described previously^136,137^, with minor modifications. Briefly, the assay was carried out at 25 °C in a Reaction Buffer containing 50 mM triethanolamine-HCl (pH 7.4), 10 mM EDTA, 20 mM dithioerythritol, 0.2 mM NADH, 10 U ml^–1^ TPI and 1 U ml^–1^ GPDH. For screening assays, a 0.2-ml reaction volume was used, and 50 nM rabbit aldolase (ammonium sulfate precipitates dissolved in 100 μl of reaction buffer) was incubated with an individual chemical compound, each at 100 μM, at 25 °C for 30 min on a glass-bottom, 96-well microplate (3635, Corning). The reaction was initiated by addition of 500 μM FBP dissolved in 100 μl of reaction buffer, followed by mixing on a SpectraMax M5 microplate reader. Data were collected using the SoftMax Pro software and exported to OriginPro software (v.9.2.0, OriginLab) for further analysis. The relative effects of each compound on aldolase activity were assessed with the rates of NAD^+^ formation during the reaction through which the OD_340_ was decreased. For assays determining the activities of aldolase purified from lysosomes and cytosol, 1 μg of protein was used, and the assay was performed in the same way as that in drug screening assays. For other assays that quantified the absolute activity of aldolase, a 1.8-ml reaction volume was used. Briefly, some 1768.2 μl of reaction buffer was added into a 3-ml cuvette on a Lambda 365 Spectrophotometer (PerkinElmer) equipped with magnetic stirrer, with a stirring speed of 140 r.p.m., and temperature set to 25 °C. After 3 min of incubation, the cuvette was taken out and was gently tapped to remove the bubbles. The cuvette was incubated on the spectrophotometer until the baseline OD_340_ became flat and smooth, with the change of baseline OD_340_ value less than 0.001. The reaction was initiated through addition of 30 μl of 50 nM rabbit aldolase (ammonium sulfate precipitates), or 100 nM His-tagged aldolase (bacterially expressed and purified) pre-incubated with compounds at the desired concentration at 25 °C for 30 min, to the cuvette. Data were collected using UV WinLab software (v.7.1.0.68, PerkinElmer) and exported to OriginPro software for further analysis. The concentrations of FBP consumed were calculated by the extinction coefficient for NADH at 340 nm, which is 6,220 cm^–1^ M^–1^.

To determine the activity of lysosome-bound aldolase, a hydrazine-based calorimetric assay in which the 3-phosphoglyceraldehyde reacts with hydrazine to form hydrazone that absorbs at 240 nm (ref. ^138^), was performed. In brief, lysosomes purified from 80 10-cm dishes of MEFs were collected and washed with PBS. Lysosomes were incubated with EDTA (0.1 mM final concentration), iodoacetate (0.2 mM final concentration) and hydrazine (2.33 mM final concentration) solution to a final volume of 300 μl. A blank read was taken at 240 nm. FBP (5 mM final concentration) was added and the OD_240_ was determined at 1-min intervals by a SpectraMax M5 microplate reader (Molecular Devices). Enzymatic activity was determined in triplicate and normalized to the control.

### Kinome screening

The KINOME*scan* profiling was carried out as described previously^139^ by Eurofins DiscoverX. Briefly, aldometanib was profiled against a panel of 468 kinases using KINOME*scan* technology, which is an active site-dependent competition binding assay at 1 µM. Results were reported as a percentage of control DMSO (ctrl%), in which lower values represent higher affinity binding; ctrl% = (signal of test compound − signal of positive control)/(signal of negative control − signal of positive control) × 100; where the negative control was set at 100%, and the positive control compound was set at 0%. The value of 35% represents moderate binding affinity.

### Determination of aldometanib pharmacokinetics

To determine the pharmacokinetics of aldometanib, cells collected from a 10-cm dish (60–70% confluence), lysosomes purified from 40 10-cm dishes of cells, mitochondria purified from 20 10-cm dishes of cells, cytosol purified from 2 10-cm dishes of cells, 100 µl of serum or 150 nematodes were required. Samples were lysed with 1 ml of ice-cold 80% (vol/vol) methanol in water containing 2.3 ng ml^−1^ d_3_-l-carnitine C16:0 as an internal standard, followed by centrifuging at 20,000*g* for 15 min at 4 °C. Some 600 µl of supernatant was collected, lyophilized in a vacuum concentrator (CentriVap Benchtop Centrifugal Vacuum Concentrator, equipped with a CentriVap −84 °C Cold Trap and a Scroll Vacuum Pump, Labconco) at 4 °C, and then dissolved in 100 µl of 70% (vol/vol, in water) methanol. Samples were analysed on a QTRAP MS (QTRAP 6500+, SCIEX) interfaced with a UPLC system (Acquity I-class, Waters). Some 2 µl of each sample was loaded onto a reverse-phase column (ACQUITY UPLC BEH C18, 1.7 μm, 2.1 × 50 mm; 186002350, Waters). The mobile phase consisted of 0.1% formic acid in LC–MS-grade water (mobile phase A) and LC–MS-grade methanol (mobile phase B) run at a flow rate of 0.2 ml min^−1^. The analytes were separated with the following gradient programme: 70% B increased to 100% B in 5 min, held for 2 min, and the post time was set to 3 min. The QTRAP mass spectrometer used a Turbo V ion source and ran in positive mode with a spray voltage of 5,500 V, source temperature of 400 °C, gas 1 of 40 psi and gas 2 of 50 psi and curtain gas of 40 psi. Aldometanib were measured using the multiple reaction monitoring (MRM) mode, and declustering potentials and collision energies were optimized through the use of analytical standards. The following transitions were used for monitoring each compound: 465.3/240.6 and 465.3/159.1 for aldometanib and 403.4/85 for d_3_-l-carnitine C16:0 as internal standard. Data were collected using Analyst software (v.1.6.3, SCIEX), and the relative amounts of aldometanib were analysed using MultiQuant software (v.3.0.2, SCIEX). The half-life of aldometanib was determined by Phoenix WinNonlin software (v.8.1, Certara).

### Determination of the binding affinity of aldometanib for aldolase

The binding affinity of aldometanib for aldolase was determined through an SPR assay. Briefly, experiments were performed at 25 °C on a BIAcore 8K instrument (GE Healthcare) using CM5 sensor chips, and data were analysed using evaluation software equipped for the BIAcore 8K instrument following the manufacturer’s instructions. In brief, a cell on the CM5 sensor chip was activated with a mixture of 200 μM 1-ethyl-3-(3-dimethylaminopropyl)carbodiimide and 50 μM NHS at 10 μl min^−1^ for 10 min. A total of 0.5 μl of rabbit aldolase protein (7 mg ml^−1^) adjusted to pH 4.0 (by mixing with 150 μl of 10 mM sodium acetate solution, pH 4.0) was then immobilized on the surface of the cell at 10 μl min^−1^ for 5 min. The cell was then blocked with 1 M ethanolamine (10 μl min^−1^ for 10 min). A neighbouring cell that served as a reference was similarly activated and blocked, except that PBS adjusted to pH 4.0 was used for immobilization. Both of the cells were then equilibrated with PBS-P. Aldometanib stock solution (10 mM) was diluted to a series of concentrations (all in PBS-P), and was flowed at 30 μl min^−1^ for 120 s of association and 200 s of dissociation in each run. At the end of each flow, cells were regenerated for 30 s with 10 mM glycine-HCl (pH 1.5) solution at 30 μl min^−1^. Data from the sample cell were collected, and were subtracted by those from the reference cell. The association (*K*_a_) and dissociation (*K*_d_) constants were obtained by global fitting of the data to a 1:1 Langmuir binding model using Biacore Insight Evaluation Software (v.3.0.11.15423) for the BIAcore 8K. Data were exported to Origin 7 software (v.7.0552, OriginLab) for generation of the final figures.

### Measurements of glycolytic intermediates, adenylates and NAD^+^

Levels of glycolytic intermediates were analysed by capillary electrophoresis-based mass spectrometry (CE–MS) as described previously^14^. Briefly, each measurement required cells collected from one 10-cm dish (60–70% confluence). Cells were incubated in glucose-free DMEM supplemented with 25 mM [U-^13^C]-glucose and 10% FBS for another 10 min, followed by rinsing with 20 ml of 5% (mass/vol) mannitol solution (dissolved in water), and instantly freezing in liquid nitrogen. Cells were then lysed with 1 ml of methanol containing IS1 (used to standardize the metabolite intensity and to adjust the migration time). The lysate was then mixed with 1 ml of chloroform and 400 μl of water by 20 s of vortexing. After centrifugation at 15,000*g* for 15 min at 4 °C, 450 μl of aqueous phase was collected and was then filtrated through a 5-kDa-cutoff filter (UFC3LCCNB-HMT, Millipore) by centrifuging at 12,000*g* for 3 h at 4 °C. In parallel, quality-control samples were prepared by combining 10 μl of the aqueous phase from each sample and then similarly filtered. The filtered aqueous phase was then freeze dried in a vacuum concentrator at 4 °C, and then dissolved in 100 μl of water containing IS3 (to adjust the migration time). Then, 20 μl of redissolved solution was then loaded into an injection vial with a conical insert for CE-QTOF MS (Agilent Technologies 7100, equipped with 6545 mass spectrometer) analysis. Data were collected using MassHunter LC–MS acquisition 10.1.48 (Agilent), and were processed using Qualitative Analysis B.06.00 (Agilent).

To determine levels of ATP, ADP, AMP and NAD^+^ in cells, mouse tissues or nematodes, high-performance liquid chromatography (HPLC)–MS was performed. For cells, a 10-cm dish (60–70% confluence) of cells was collected as in CE–MS. For mouse tissues, 50 mg of freshly excised (using a freeze clamp) tissue was immediately frozen in liquid nitrogen, and homogenized in 1 ml of ice-cold methanol. For nematodes, 150 nematodes maintained on NGM (containing aldometanib or not) for 2 d were washed with ice-cold M9 buffer containing Triton X-100, followed by removing bacteria by quickly centrifuging the slurry at 100*g* for 5 s, and then instantly lysing in 1 ml of methanol. The lysates were then mixed with 1 ml of chloroform and 400 µl of water (containing 4 µg ml^−1^ [U-^13^C]-glutamine), followed with 20 s of vortexing. After centrifugation at 15,000*g* for another 15 min at 4 °C, 800 µl of aqueous phase was collected, lyophilized in a vacuum concentrator at 4 °C, and then dissolved in 30 µl of 50% (vol/vol, in water) acetonitrile. Measurements of adenylate and NAD^+^ levels were based on ref. ^140^, using a QTRAP MS (QTRAP 5500, SCIEX) interfaced with a UPLC system (ExionLC AD, SCIEX). THen, 2 µl of each sample were loaded onto a HILIC column (ZIC-pHILIC, 5 μm, 2.1 × 100 mm; 1.50462.0001, Millipore). The mobile phase consisted of 15 mM ammonium acetate containing 3 ml l^−1^ ammonium hydroxide (>28%, vol/vol) in the LC–MS-grade water (mobile phase A) and LC–MS-grade 90% (vol/vol) acetonitrile in LC–MS-grade water (mobile phase B) run at a flow rate of 0.2 ml min^−1^. Metabolites were separated with the following HPLC gradient elution programme: 95% B held for 2 min, then to 45% B in 13 min, held for 3 min, and then back to 95% B for 4 min. The mass spectrometer was run on a Turbo V ion source in negative mode with a spray voltage of −4,500 V, source temperature of 550 °C, gas no.1 of 50 psi, gas no. 2 of 55 psi and curtain gas of 40 psi. Metabolites were measured using the MRM mode, and declustering potentials and collision energies were optimized through use of analytical standards. The following transitions were used for monitoring each compound: 505.9/158.9 and 505.9/408.0 for ATP; 425.9/133.9, 425.9/158.8 and 425.9/328.0 for ADP; 345.9/79.9, 345.9/96.9 and 345.9/133.9 for AMP, 662.0/540.1 for NAD^+^, and 149.9/114 for [U-^13^C]-glutamine. Data were collected using Analyst software (v.1.7.1, SCIEX), and the relative amounts of metabolites were analysed using MultiQuant software (v.3.0.3, SCIEX). Note that a portion of ADP and ATP could lose one or two phosphate groups during in-source fragmentation thus leaving the same *m/z* ratios as AMP and ADP, which were corrected according to their different retention times in column.

### Determination of muscular 2-deoxy-d-glucose uptake

Muscular glucose uptake, as assessed by 2-DG uptake and its conversion to 2-DG6P, was determined as described previously^37^, with minor modifications. Briefly, cells grown on a 10-cm dish (60–70% confluence) were treated with aldometanib for 2 h. At 10 min before collection, 0.1 mM 2-DG was added to the culture medium. For muscle tissues, mice were starved for 5 h (8:00 to 13:00), followed by orally gavaging aldometanib. Some 1 h later, mice were intraperitoneally injected with 1.25 mpk glucose supplemented with 0.125 mpk 2-DG (dissolved in water) at 10 μl per gram. Some 1 h later, 70 mg of gastrocnemius muscle was excised and was immediately frozen in liquid nitrogen, followed by homogenizing in 1 ml of ice-cold 80% (vol/vol) methanol in water containing 4 µg ml^−1^ [U-^13^C]-glutamine as an internal standard, followed by centrifuging at 20,000*g* for 15 min at 4 °C. Some 600 µl of supernatant was collected, lyophilized in a vacuum concentrator at 4 °C, and then dissolved in 400 µl of 50% (vol/vol, in water) acetonitrile. The 2-DG and 2-DG6P were determined on an ExionLC AD liquid chromatography combined with QTRAP 5500 mass spectrometer. Chromatographic separations were performed with a Millipore ZIC-pHILIC column (5 μm, 2.1 × 100 mm internal dimensions; 1.50462.0001). The column was maintained at 40 °C and the injection volume of all samples was 2 μl. The mobile phase consisted of 15 mM ammonium acetate and 3 ml l^−1^ ammonium hydroxide (>28%) in LC–MS-grade water (mobile phase A) and LC–MS-grade 90% (vol/vol) acetonitrile in HPLC water (mobile phase B) run at a flow rate of 0.25 ml min^−1^. The analytes were separated with the following gradient programme: 95% B held for 1 min, increased to 50% B in 6 min, held for 1 min, and the post time was set 2 min. The QTRAP mass spectrometer used an Turbo V ion source. The ion source was run in negative mode with a spray voltage of −4,500 V, gas 1 of 50 psi, gas 2 of 55 psi and curtain gas of 35 psi. Metabolites were measured using MRM, and declustering potentials and collision energies were optimized through use of analytical standards. The following transitions were used for monitoring each compound: 163.0/85.0 and 163.0/119.0 for 2-DG; 243.0/96.9, 243.0/78.9 and 243.0/139.0 for 2-DG6P and 149.9/114 for [U-^13^C]-glutamine. Data were collected and processed as in adenylates or NAD^+^ measurement.

### Determination of triacylglycerol synthesis

TAG synthesis rates were determined by the contents of labelled TAG in cells and mice treated with [U-^13^C]-glucose (dissolved in PBS). Primary hepatocytes were isolated and cultured in DMEM containing 1% BSA for 12 h before the experiment. Cells were rinsed twice with PBS, and then incubated in glucose-free DMEM supplemented with 1 mM sodium pyruvate, 25 mM [U-^13^C]-glucose and 1% BSA for another 12 h. Cells on a 10-cm dish were rinsed with 25 ml of PBS twice, and instantly frozen in liquid nitrogen, followed by lysing with 1 ml of methanol containing TAG (15:0/15:0/15:0) as an internal standard, and were collected after scraping. Mice were cannulated on their right jugular veins as in the glucose clamp experiments, and were fasted for 6 h (8:00 to 14:00). Next, 200 mM [U-^13^C]-glucose was then infused into a mouse at a constant rate of 0.2 μl min^−1^ per gram body weight for 2.5 h (titrated according to ref. ^141^). At the end of the infusion, the mouse was anaesthetized, and 100 mg of liver tissue was collected by freeze clamping, followed by immediately freezing in liquid nitrogen and homogenized in 1 ml of methanol containing TAG (15:0/15:0/15:0) as an internal standard. Lysates of cells or liver tissues were then quickly mixed each with 1 ml of chloroform and 400 μl of water by 20 s of vortexing. After centrifugation at 15,000*g* for 15 min at 4 °C, 700 μl of organic phase of each sample was collected, followed by lyophilization with nitrogen blow at room temperature. Analysis of TAG was performed on a Prominence UPLC system (Nexera UHPLC LC-30A, Shimadzu) interfaced with TripleTOF 5600+ system (SCIEX) equipped with ESI source. The lyophilized sample was dissolved in 20 µl of dichloromethane/methanol solution (2:1, vol/vol), and was diluted with 380 µl of methanol/isopropanol/H_2_O solution (65:30:5, vol/vol/vol). The injection volume was 5 µl. TAG contents were separated through a C8 column (2.1 × 100 mm with 1.7-μm particle size; 186002878, Waters) with column temperature maintained at 55 °C. Mobile phases consist of 10 mM ammonium formate in acetonitrile/H_2_O (60:40, vol/vol; mobile phase A) and 10 mM ammonium formate in isopropanol/acetonitrile (90:10, vol/vol; mobile phase B) and was run at a flow rate of 0.26 ml min^−1^. The gradient was: 32% B for 1.5 min, then increased to 97% B within 19.5 min and held for 4 min, then back to 32% B and held for another 5 min. The flow rate for mobile phases was set at 0.26 ml min^−1^. The mass spectrometer was running in positive, information-dependent acquisition (IDA) mode, with the source temperature of 550 °C, the ion source gas 1 and 2 at 55 psi, the curtain gas at 35 psi, the collision energy at 40 eV, the ion spray voltage floating at 5.5 kV and the mass range at 500–1,250 *m/z*. The accumulation time for the full scan was set at 150 ms and the accumulation time for each IDA scan was 45 ms. Peaks of metabolites with intensities larger than 100 counts per second after adding up the signal from ten rounds of IDA scan were chosen for further analysis. Data were collected using Analyst TF 1.6 software (SCIEX), and were analysed using MS-DIAL 4.7 software (RIKEN), through which the deconvolution and streamline criteria were used for peak/TAG identification.

### Determination of fatty acid oxidation rates

The rates of fatty acid β-oxidation were determined through the labelled intermediates of the tricarboxylic acid cycle in cells and mice treated with [U-^13^C]-pyruvic acid (PA) for a certain time duration. PA was conjugated to BSA before use. To conjugate PA, 200 mg of PA was first dissolved in 20 ml of ethanol in a conical flask by stirring, followed by dropwise mixing with 156 μl of 5 M NaOH. The slurry was constantly stirred for 12 h, to evaporate ethanol. The dried sediment was then dissolved with 10% fatty acid-free BSA to a final concentration of 2 mM.

Primary hepatocytes were isolated and cultured in the same way as those used for the determination of TAG synthesis. Cells were incubated in DMEM containing 100 μM [U-^13^C]-PA and 1% BSA for 12 h, followed by rinsing with PBS twice and freezing in liquid nitrogen. Mice were cannulated and treated as in the determination of TAG synthesis, except that 1.85 mM [U-^13^C]-PA was infused to each mouse at a constant rate of 0.2 μl min^−1^ per gram body weight for 2.5 h (titrated according to ref. ^141^). At the end of the infusion, the mouse was anaesthetized, and 50 mg of liver tissue was collected by freeze clamping, followed by immediately freezing in liquid nitrogen. Cells and liver tissues were then lysed with 1 ml of 80% methanol (vol/vol) in water containing 10 µg ml^−1^ myristic-d27 acid as an internal standard, followed with 20 s of vortexing. After centrifugation at 15,000*g* for 15 min at 4 °C, 600 µl of supernatant (aqueous phase) was freeze dried at 4 °C. The lyophilized sample was then vortexed for 1 min after mixing with 50 µl of freshly prepared methoxyamine hydrochloride (20 mg ml^−1^ in pyridine), followed by incubation at 4 °C for 1 h. The mixture was sonicated at 0 °C by bathing in ice slurry for 10 min, and was then incubated at 37 °C for 1.5 h, followed by mixing with 50 µl of MTBSTFA and incubated at 55 °C for 1 h. Before subjecting the sample to gas chromatography–mass spectrometry (GC–MS), the sample was centrifuged at 15,000*g* for 10 min, and 60 μl of supernatant was loaded into an injection vial. GC was performed on a HP-5MS column (30 m × 0.25 mm internal diameter, 0.25 μm film thickness; 19091S-433) using a GC/MSD instrument (7890-5977B, Agilent). The injector temperature was 260 °C. The column oven temperature was first held at 70 °C for 2 min, then increased to 180 °C at the rate of 7 °C min^−1^, then to 250 °C at the rate of 5 °C min^−1^ then to 310 °C at the rate of 25 °C min^−1^, where it was held for 15 min. The MSD transfer temperature was 280 °C. The MS quadrupole and source temperatures were maintained at 150 °C and 230 °C, respectively. Data were collected using MassHunter GC–MS Acquisition software (v.B.07.04.2260, Agilent), and were analysed using GC–MS MassHunter Workstation Qualitative Analysis software (v.B.07.01SP1, Agilent).

### Determination of faecal long-chain fatty acids

Some 0.1 g of fresh faecal sample was homogenized with 1 ml of methanol containing 100 μg ml^−1^ tridecanoic acid as an internal standard, followed by mixing with 1 ml of chloroform and 400 μl of distilled water by 20 s of vortexing. After centrifugation at 15,000*g* for 15 min at 4 °C, 600 μl of organic (lower) phase was collected, followed by lyophilization with nitrogen blow at room temperature. The lyophilized sample was then vortexed for 1 min after mixing with 1 ml of freshly prepared 1% H_2_SO_4_ (vol/vol in methanol), followed by incubating at 80 °C in oil bath for 1 h. The esterified products were then extracted by vigorously mixing with 2 ml of hexane for three times at room temperature. The organic (upper) phase was collected and combined, and was then dried in the nitrogen flow at room temperature. Before subjecting to GC–MS, the sample was dissolved in 1 ml of hexane, followed by centrifuging at 15,000*g* for 10 min, and some 60 μl of supernatant was loaded into an injection vial. GC was performed on a CP-Wax 52 CB column (30 m × 0.25 mm internal diameter, 0.25 μm film thickness; CP87131) using a 7890B (Agilent Technologies) instrument fitted with an MS detector (5977B Agilent). The injector temperature was set at 260 °C. The column oven temperature was initially held at 50 °C for 3 min, then increased to 170 °C at the rate of 10 °C min^−1^, then to 205 °C at the rate of 3 °C min^−1^, where it was held for 20 min, then to 235 °C at the rate of 5 °C min^−1^, and then to 250 °C at the rate of 10 °C min^−1^, where it was held for 3 min. The MSD transfer temperature was 280 °C. The MS quadrupole and source temperatures were maintained at 150 °C and 230 °C, respectively. Data were collected using MassHunter GC–MS Acquisition software (v.B.07.04.2260, Agilent), and were analysed using GC–MS MassHunter Workstation Qualitative Analysis software (v.B.07.01SP1, Agilent), followed by normalization to internal standard and dry protein weight.

### Determination of fructose-1,6-bisphosphate and aldometanib binding of aldolase

Binding of FBP by aldolase was determined as described previously^64^. Briefly, 1 μg of rabbit aldolase was mixed with aldometanib at the desired concentration in 40 μl of HEPES buffer (50 mM HEPES, pH 8.0) at 25 °C for 2 h, followed by mixing with 10 μl of 50 mM FBP (dissolved in HEPES buffer) for another 20 min on ice. Some 80 μl of 200 mM NaBH_4_ dissolved in MES buffer (prepared by gradually adding NaBH_4_ powder into a large volume, such as 200 ml of ice-cold MES buffer (200 mM MES, pH 6.0) with continuous stirring) was added to the mixture to trap Schiff-base intermediates as described previously^142^, followed by incubation for 20 min on ice. The reaction was terminated by mixing with 130 μl of 2× SDS sample buffer for SDS–PAGE. After staining with 1% Coomassie Brilliant Blue R-250 (dissolved in 45% methanol and 10% acetic acid in water) for 30 min, the SDS–PAGE gels were decoloured with staining solution without R-250 dye. The excised gel slices containing the aldolase band were subjected to in-gel trypsin digestion and dried. Samples were analysed on a nanoElute (Bruker) coupled to a timsTOF Pro (Bruker) equipped with a CaptiveSpray source. Peptides were dissolved in 10 μl 0.1% formic acid and were loaded onto a homemade C18 column (35 cm × 75 μm internal diameter, 1.9 μm, 100 Å). Samples were then eluted for 60 min with linear gradients of 3–35% acetonitrile (in 0.1% formic acid) at a flow rate of 0.3 μl min^−1^. MS data were acquired with a timsTOF Pro mass spectrometer (Bruker) operated in PASEF mode, and were analysed using Peaks Studio software (X+, Bioinformatics Solutions) and compared against the UniProt database. According to the catalytic mechanisms reported^142^, an adduct with 319.97 Dalton on Lys230 residue represented a phosphoglucitoylation (six-carbon) modification, or FBP binding, while 151.99 Dalton represented DHAP (three-carbon phosphoglycerolyation).

For examining the binding of aldometanib on aldolase, the aldometanib-conjugated rabbit aldolase was subjected to SDS–PAGE and MS, and were processed as described above, except that an adduct with 465.28 Dalton on the Lys230 residue represented a conjugation of aldometanib.

### Determination of inhibitory effects of aldometanib on aldolase fructose-1,6-bisphosphate binding in situ

The inhibitory effects of aldometanib on aldolase in situ were determined through a single-cell analyser customized by Jiangsu Rayme Biotechnology. The analyser was modified/upgraded from model no. SCA 500, and consists of five parts: (a) a single-cell excitation light source system through which the GCaMP6s-TRPV4 indicator expressed in a specific cell could be excited by a 10-μm (tip diameter) microstructured optical fibre transmitting a laser light beam at 488 nm; (b) an ultrasensitive fluorescence detecting system that adopts a high-sensitivity fluorescence detection module through which the fluorescence signal from a single cell could be detected in real time; (c) an ultra-micro-volume injection system consisting an air-driving pump interfaced with a microinjection pipette with its tip diameter below 500 nm, through which the femtolitre levels of FBP loaded on pipette could be injected to a specific cell; (d) a nanomanipulation or micromanipulation system consisting of two sets of automatic micromanipulation arms that can move automatically at the *x*, *y* and *z* axes at 50-nm accuracies, through which the microstructured optical fibre and the microinjection pipette can be held and adjusted onto a specific cell; and (e) an imaging system consisting an inverted fluorescence microscope and a charged-coupled device for observing cells in real time and adjusting microinjection/fluorescence detection systems.

Before the experiment, the injection needle was slowly loaded with approximately 10 μl of FBP solution (1 mM, dissolved in PBS), to the position that the liquid level of FBP solution could be reached by the electrode of the electric-driving pump, and was then fixed on the left automatic micromanipulation arm. The fluorescence detector was fixed on the right automatic micromanipulation arm. HEK293T cells stably expressing TRPV4-GCaMP6s were grown on a 35-mm dish (D35-20-10-N, In Vitro Scientific) to 20% confluence and were visualized under the Bright Field mode on the injection chamber (kept at 37 °C, 5% CO_2_) of the single-cell analyser. The chamber, and the left and right automatic micromanipulation arms were adjusted to make sure that a centred view of cells, injection needle and fluorescence detector could be obtained. The injection needle was lowered to penetrate the cytosolic area of a cell, as was the fluorescence detector, except that the tip of the detector just touched, but did not pierce, the cell membrane. The fluorescence of TRPV4-GCaMP6s was detected and recorded under the recording mode (dark field) after exciting by the 488-nm laser. FBP was microinjected under the following programmes: initial voltage, 0 V; high voltage, 5 V; low voltage, −5 V; injection time, 10 s. Data were collected and processed using SC analysis software (v.1.0, Rayme).

### Statistics and reproducibility

Statistical analyses were performed using Prism 9 (GraphPad Software), except for the Mantel–Cox test (for analysing survival curves) and ANCOVA (for analysing EE of mice), which were performed using SPSS 27.0 (IBM). Each group of data was subjected to the Kolmogorov–Smirnov test, Anderson–Darling test, D’Agostino–Pearson omnibus test or Shapiro–Wilk test for normal distribution when applicable. An unpaired two-tailed Student’s *t*-test was used to determine significance between two groups of normally distributed data. Welch’s correction was used for groups with unequal variances. An unpaired two-tailed Mann–Whitney test was used to determine significance between data without a normal distribution. For comparisons between multiple groups, an ordinary one-way or two-way ANOVA was used, followed by Tukey’s, Sidak’s, Dunnett’s or Dunn’s test. ANCOVA was used to compare the oxygen consumption and EE of the mice, while statistically controlling for the body weight of each mouse as the covariate, as indicated in the figure legends. The assumptions of homogeneity of error variances were tested using an *F*-test (*P* > 0.05). For comparison between multiple groups with two fixed factors, an ordinary two-way ANOVA or two-way RM ANOVA (for example, for GTT and ITT data) was used, followed by Tukey’s or Sidak’s multiple-comparisons test. Geisser–Greenhouse correction was used where applicable. The adjusted means and s.e.m., or s.d., were recorded when the analysis met the above standards. Differences were considered significant when *P* < 0.05, or *P* > 0.05 with large differences of observed effects (as suggested in refs. ^143,144^). All specific statistical details can be found in the figure captions and statistical data. All images shown without biological replicates are representative of a minimum of three independent experiments.

### Reporting summary

Further information on research design is available in the Nature Research Reporting Summary linked to this article.

## Supplementary information

Supplementary Information Supplementary Tables 1–5 and ‘Synthetic procedures for aldometanib and its derivatives’

Reporting Summary

## Data Availability

The analysis was performed using standard protocols with previously described analysis tools. No custom code was used in this study. The MS proteomics data have been deposited to the ProteomeXchange Consortium (http://proteomecentral.proteomexchange.org/) through the iProX partner repository^145^ under the dataset identifier PXD035968. Materials, reagents or other experimental data are available upon request. Source data are provided with this paper. Other data such as statistical analyses are also provided.

## Source data

Source Data Fig. 1 Statistical source data.
Source Data Fig. 1 Unprocessed western blots.
Source Data Fig. 2 Statistical source data.
Source Data Fig. 2 Unprocessed western blots.
Source Data Fig. 3 Statistical source data.
Source Data Fig. 3 Unprocessed western blots.
Source Data Fig. 4 Statistical source data.
Source Data Fig. 5 Statistical source data.
Source Data Fig. 5 Unprocessed western blots.
Source Data Fig. 6 Statistical source data.
Source Data Fig. 6 Unprocessed western blots.
Source Data Fig. 7 Statistical source data.
Source Data Fig. 8 Statistical source data.
Source Data Extended Data Fig. 1 Statistical source data.
Source Data Extended Data Fig. 2 Statistical source data.
Source Data Extended Data Fig. 3 Statistical source data.
Source Data Extended Data Fig. 3 Unprocessed western blots.
Source Data Extended Data Fig. 4 Statistical source data.
Source Data Extended Data Fig. 4 Unprocessed western blots.
Source Data Extended Data Fig. 5 Statistical source data.
Source Data Extended Data Fig. 5 Unprocessed western blots.
Source Data Extended Data Fig. 6 Statistical source data.
Source Data Extended Data Fig. 6 Unprocessed western blots.
Source Data Extended Data Fig. 7 Statistical source data.
Source Data Extended Data Fig. 7 Unprocessed western blots.
Source Data Extended Data Fig. 8 Statistical source data.
Source Data Extended Data Fig. 8 Unprocessed western blots.
Source Data Extended Data Fig. 9 Statistical source data.
Source Data Extended Data Fig. 9 Unprocessed western blots.
Source Data Extended Data Fig. 10 Statistical source data.
