# Supplementary material for: The aldolase inhibitor aldometanib mimics glucose starvation to activate lysosomal AMPK
Source: Nat Metab. 2022 Oct 10;4(10):1369–401. doi: 10.1038/s42255-022-00640-7 (PMC9584815; doi:10.1038/s42255-022-00640-7)
Supplement: Supplementary file 1 — Supplementary Tables 1–5 and ‘Synthetic procedures for aldometanib and its derivatives’ [file 42255_2022_640_MOESM1_ESM.pdf]

nature metabolism

Article https://doi.org/10.1038/s42255-022-00640-7

The aldolase inhibitor aldometanib mimics glucose starvation to activate lysosomal AMPK

In the format provided by the authors and unedited

Supplementary Table 1. Full list of KINOME*scan* profiling data of Aldometanib. Aldometanib at 1 μM was profiled against a panel of 468 kinases using the KINOME*scan* technology, which is an active site-dependent competition binding assay. Results were reported as the remaining enzymatic activity (percent of control DMSO). The value of 35% represents moderate binding affinity.

**Compound ID**: **Aldometanib (1 μM)**		
**DiscoveRx Gene Symbol**	**Entrez Gene Symbol**	**Remaining enzymatic activity (%)**
AAK1	AAK1	95
ABL1(E255K)-phosphorylated	ABL1	87
ABL1(F317I)-nonphosphorylated	ABL1	100
ABL1(F317I)-phosphorylated	ABL1	99
ABL1(F317L)-nonphosphorylated	ABL1	99
ABL1(F317L)-phosphorylated	ABL1	100
ABL1(H396P)-nonphosphorylated	ABL1	99
ABL1(H396P)-phosphorylated	ABL1	81
ABL1(M351T)-phosphorylated	ABL1	100
ABL1(Q252H)-nonphosphorylated	ABL1	90
ABL1(Q252H)-phosphorylated	ABL1	94
ABL1(T315I)-nonphosphorylated	ABL1	100
ABL1(T315I)-phosphorylated	ABL1	99
ABL1(Y253F)-phosphorylated	ABL1	76
ABL1-nonphosphorylated	ABL1	83
ABL1-phosphorylated	ABL1	77
ABL2	ABL2	100
ACVR1	ACVR1	91
ACVR1B	ACVR1B	94
ACVR2A	ACVR2A	99
ACVR2B	ACVR2B	93
ACVRL1	ACVRL1	98
ADCK3	CABC1	100
ADCK4	ADCK4	99
AKT1	AKT1	100
AKT2	AKT2	100
AKT3	AKT3	100
ALK	ALK	46
ALK(C1156Y)	ALK	42
ALK(L1196M)	ALK	69
AMPK-alpha1	PRKAA1	94
AMPK-alpha2	PRKAA2	100
ANKK1	ANKK1	100

ARK5	NUAK1	100
ASK1	MAP3K5	100
ASK2	MAP3K6	56
AURKA	AURKA	86
AURKB	AURKB	100
AURKC	AURKC	95
AXL	AXL	100
BIKE	BMP2K	100
BLK	BLK	98
BMPR1A	BMPR1A	100
BMPR1B	BMPR1B	100
BMPR2	BMPR2	100
BMX	BMX	94
BRAF	BRAF	89
BRAF(V600E)	BRAF	100
BRK	PTK6	98
BRSK1	BRSK1	97
BRSK2	BRSK2	96
BTK	BTK	83
BUB1	BUB1	89
CAMK1	CAMK1	84
CAMK1B	PNCK	78
CAMK1D	CAMK1D	100
CAMK1G	CAMK1G	89
CAMK2A	CAMK2A	87
CAMK2B	CAMK2B	91
CAMK2D	CAMK2D	96
CAMK2G	CAMK2G	91
CAMK4	CAMK4	100
CAMKK1	CAMKK1	97
CAMKK2	CAMKK2	96
CASK	CASK	37
CDC2L1	CDK11B	100
CDC2L2	CDC2L2	100
CDC2L5	CDK13	89
CDK11	CDK19	100
CDK2	CDK2	91
CDK3	CDK3	100
CDK4	CDK4	100
CDK4-cyclinD1	CDK4	100
CDK4-cyclinD3	CDK4	90
CDK5	CDK5	92

CDK7	CDK7	64
CDK8	CDK8	100
CDK9	CDK9	100
CDKL1	CDKL1	77
CDKL2	CDKL2	100
CDKL3	CDKL3	100
CDKL5	CDKL5	92
CHEK1	CHEK1	95
CHEK2	CHEK2	79
CIT	CIT	100
CLK1	CLK1	100
CLK2	CLK2	94
CLK3	CLK3	100
CLK4	CLK4	96
CSF1R	CSF1R	94
CSF1R-autoinhibited	CSF1R	94
CSK	CSK	100
CSNK1A1	CSNK1A1	83
CSNK1A1L	CSNK1A1L	96
CSNK1D	CSNK1D	100
CSNK1E	CSNK1E	100
CSNK1G1	CSNK1G1	100
CSNK1G2	CSNK1G2	92
CSNK1G3	CSNK1G3	100
CSNK2A1	CSNK2A1	98
CSNK2A2	CSNK2A2	67
CTK	MATK	89
DAPK1	DAPK1	100
DAPK2	DAPK2	88
DAPK3	DAPK3	93
DCAMKL1	DCLK1	100
DCAMKL2	DCLK2	100
DCAMKL3	DCLK3	100
DDR1	DDR1	100
DDR2	DDR2	83
DLK	MAP3K12	74
DMPK	DMPK	100
DMPK2	CDC42BPG	90
DRAK1	STK17A	92
DRAK2	STK17B	95
DYRK1A	DYRK1A	96
DYRK1B	DYRK1B	92

DYRK2	DYRK2	99
EGFR	EGFR	55
EGFR(E746-A750del)	EGFR	86
EGFR(G719C)	EGFR	90
EGFR(G719S)	EGFR	80
EGFR(L747-E749del, A750P)	EGFR	95
EGFR(L747-S752del, P753S)	EGFR	72
EGFR(L747-T751del,Sins)	EGFR	85
EGFR(L858R)	EGFR	71
EGFR(L858R,T790M)	EGFR	51
EGFR(L861Q)	EGFR	100
EGFR(S752-I759del)	EGFR	88
EGFR(T790M)	EGFR	91
EIF2AK1	EIF2AK1	89
EPHA1	EPHA1	100
EPHA2	EPHA2	100
EPHA3	EPHA3	100
EPHA4	EPHA4	84
EPHA5	EPHA5	95
EPHA6	EPHA6	97
EPHA7	EPHA7	100
EPHA8	EPHA8	100
EPHB1	EPHB1	96
EPHB2	EPHB2	100
EPHB3	EPHB3	100
EPHB4	EPHB4	100
EPHB6	EPHB6	100
ERBB2	ERBB2	99
ERBB3	ERBB3	82
ERBB4	ERBB4	91
ERK1	MAPK3	99
ERK2	MAPK1	100
ERK3	MAPK6	85
ERK4	MAPK4	98
ERK5	MAPK7	99
ERK8	MAPK15	100
ERN1	ERN1	100
FAK	PTK2	51
FER	FER	66
FES	FES	88
FGFR1	FGFR1	100
FGFR2	FGFR2	100

FGFR3	FGFR3	100
FGFR3(G697C)	FGFR3	100
FGFR4	FGFR4	98
FGR	FGR	100
FLT1	FLT1	100
FLT3	FLT3	100
FLT3(D835H)	FLT3	99
FLT3(D835V)	FLT3	70
FLT3(D835Y)	FLT3	100
FLT3(ITD)	FLT3	100
FLT3(ITD,D835V)	FLT3	92
FLT3(ITD,F691L)	FLT3	84
FLT3(K663Q)	FLT3	97
FLT3(N841I)	FLT3	70
FLT3(R834Q)	FLT3	86
FLT3-autoinhibited	FLT3	95
FLT4	FLT4	100
FRK	FRK	100
FYN	FYN	100
GAK	GAK	97
GCN2(Kin.Dom.2,S808G)	EIF2AK4	100
GRK1	GRK1	84
GRK2	ADRBK1	100
GRK3	ADRBK2	100
GRK4	GRK4	83
GRK7	GRK7	75
GSK3A	GSK3A	100
GSK3B	GSK3B	100
HASPIN	GSG2	100
HCK	HCK	87
HIPK1	HIPK1	85
HIPK2	HIPK2	75
HIPK3	HIPK3	98
HIPK4	HIPK4	83
HPK1	MAP4K1	95
HUNK	HUNK	78
ICK	ICK	83
IGF1R	IGF1R	89
IKK-alpha	CHUK	100
IKK-beta	IKBKB	100
IKK-epsilon	IKBKE	97
INSR	INSR	94

INSRR	INSRR	100
IRAK1	IRAK1	100
IRAK3	IRAK3	100
IRAK4	IRAK4	93
ITK	ITK	100
JAK1(JH1domain-catalytic)	JAK1	100
JAK1(JH2domain-pseudokinase)	JAK1	84
JAK2(JH1domain-catalytic)	JAK2	39
JAK3(JH1domain-catalytic)	JAK3	92
JNK1	MAPK8	97
JNK2	MAPK9	82
JNK3	MAPK10	69
KIT	KIT	83
KIT(A829P)	KIT	100
KIT(D816H)	KIT	100
KIT(D816V)	KIT	100
KIT(L576P)	KIT	89
KIT(V559D)	KIT	95
KIT(V559D,T670I)	KIT	80
KIT(V559D,V654A)	KIT	100
KIT-autoinhibited	KIT	49
LATS1	LATS1	68
LATS2	LATS2	63
LCK	LCK	86
LIMK1	LIMK1	85
LIMK2	LIMK2	79
LKB1	STK11	100
LOK	STK10	95
LRRK2	LRRK2	48
LRRK2(G2019S)	LRRK2	39
LTK	LTK	60
LYN	LYN	100
LZK	MAP3K13	100
MAK	MAK	74
MAP3K1	MAP3K1	99
MAP3K15	MAP3K15	87
MAP3K2	MAP3K2	86
MAP3K3	MAP3K3	88
MAP3K4	MAP3K4	100
MAP4K2	MAP4K2	98
MAP4K3	MAP4K3	100
MAP4K4	MAP4K4	93

MAP4K5	MAP4K5	100
MAPKAPK2	MAPKAPK2	100
MAPKAPK5	MAPKAPK5	100
MARK1	MARK1	97
MARK2	MARK2	100
MARK3	MARK3	85
MARK4	MARK4	100
MAST1	MAST1	100
MEK1	MAP2K1	100
MEK2	MAP2K2	100
MEK3	MAP2K3	60
MEK4	MAP2K4	95
MEK5	MAP2K5	98
MEK6	MAP2K6	100
MELK	MELK	97
MERTK	MERTK	93
MET	MET	47
MET(M1250T)	MET	92
MET(Y1235D)	MET	100
MINK	MINK1	80
MKK7	MAP2K7	90
MKNK1	MKNK1	100
MKNK2	MKNK2	97
MLCK	MYLK3	83
MLK1	MAP3K9	96
MLK2	MAP3K10	100
MLK3	MAP3K11	91
MRCKA	CDC42BPA	100
MRCKB	CDC42BPB	97
MST1	STK4	97
MST1R	MST1R	83
MST2	STK3	82
MST3	STK24	100
MST4	MST4	91
MTOR	MTOR	100
MUSK	MUSK	100
MYLK	MYLK	100
MYLK2	MYLK2	99
MYLK4	MYLK4	100
MYO3A	MYO3A	87
MYO3B	MYO3B	100
NDR1	STK38	83

NDR2	STK38L	93
NEK1	NEK1	100
NEK10	NEK10	82
NEK11	NEK11	72
NEK2	NEK2	98
NEK3	NEK3	85
NEK4	NEK4	100
NEK5	NEK5	96
NEK6	NEK6	85
NEK7	NEK7	100
NEK9	NEK9	96
NIK	MAP3K14	100
NIM1	MGC42105	94
NLK	NLK	100
OSR1	OXSR1	100
p38-alpha	MAPK14	100
p38-beta	MAPK11	100
p38-delta	MAPK13	87
p38-gamma	MAPK12	92
PAK1	PAK1	100
PAK2	PAK2	100
PAK3	PAK3	87
PAK4	PAK4	96
PAK6	PAK6	99
PAK7	PAK7	100
PCTK1	CDK16	96
PCTK2	CDK17	95
PCTK3	CDK18	100
PDGFRA	PDGFRA	100
PDGFRB	PDGFRB	90
PDPK1	PDPK1	95
PFCDPK1(P.falciparum)	CDPK1	100
PFPK5(P.falciparum)	MAL13P1.279	100
PFTAIRE2	CDK15	100
PFTK1	CDK14	76
PHKG1	PHKG1	98
PHKG2	PHKG2	98
PIK3C2B	PIK3C2B	100
PIK3C2G	PIK3C2G	100
PIK3CA	PIK3CA	99
PIK3CA(C420R)	PIK3CA	100
PIK3CA(E542K)	PIK3CA	89

PIK3CA(E545A)	PIK3CA	100
PIK3CA(E545K)	PIK3CA	71
PIK3CA(H1047L)	PIK3CA	82
PIK3CA(H1047Y)	PIK3CA	95
PIK3CA(I800L)	PIK3CA	100
PIK3CA(M1043I)	PIK3CA	100
PIK3CA(Q546K)	PIK3CA	79
PIK3CB	PIK3CB	96
PIK3CD	PIK3CD	100
PIK3CG	PIK3CG	100
PIK4CB	PI4KB	95
PIKFYVE	PIKFYVE	97
PIM1	PIM1	100
PIM2	PIM2	95
PIM3	PIM3	100
PIP5K1A	PIP5K1A	99
PIP5K1C	PIP5K1C	100
PIP5K2B	PIP4K2B	58
PIP5K2C	PIP4K2C	100
PKAC-alpha	PRKACA	100
PKAC-beta	PRKACB	93
PKMYT1	PKMYT1	100
PKN1	PKN1	100
PKN2	PKN2	96
PKNB(M.tuberculosis)	pknB	100
PLK1	PLK1	100
PLK2	PLK2	88
PLK3	PLK3	99
PLK4	PLK4	41
PRKCD	PRKCD	100
PRKCE	PRKCE	100
PRKCH	PRKCH	96
PRKCI	PRKCI	96
PRKCQ	PRKCQ	100
PRKD1	PRKD1	100
PRKD2	PRKD2	100
PRKD3	PRKD3	100
PRKG1	PRKG1	100
PRKG2	PRKG2	97
PRKR	EIF2AK2	100
PRKX	PRKX	100
PRP4	PRPF4B	100

PYK2	PTK2B	69
QSK	KIAA0999	99
RAF1	RAF1	100
RET	RET	100
RET(M918T)	RET	100
RET(V804L)	RET	100
RET(V804M)	RET	100
RIOK1	RIOK1	100
RIOK2	RIOK2	96
RIOK3	RIOK3	100
RIPK1	RIPK1	93
RIPK2	RIPK2	88
RIPK4	RIPK4	89
RIPK5	DSTYK	100
ROCK1	ROCK1	86
ROCK2	ROCK2	100
ROS1	ROS1	65
RPS6KA4(Kin.Dom.1-N-terminal)	RPS6KA4	100
RPS6KA4(Kin.Dom.2-C-terminal)	RPS6KA4	96
RPS6KA5(Kin.Dom.1-N-terminal)	RPS6KA5	100
RPS6KA5(Kin.Dom.2-C-terminal)	RPS6KA5	100
RSK1(Kin.Dom.1-N-terminal)	RPS6KA1	90
RSK1(Kin.Dom.2-C-terminal)	RPS6KA1	100
RSK2(Kin.Dom.1-N-terminal)	RPS6KA3	90
RSK2(Kin.Dom.2-C-terminal)	RPS6KA3	100
RSK3(Kin.Dom.1-N-terminal)	RPS6KA2	100
RSK3(Kin.Dom.2-C-terminal)	RPS6KA2	100
RSK4(Kin.Dom.1-N-terminal)	RPS6KA6	93
RSK4(Kin.Dom.2-C-terminal)	RPS6KA6	96
S6K1	RPS6KB1	100
SBK1	SBK1	98
SGK	SGK1	98
SgK110	SgK110	76
SGK2	SGK2	100
SGK3	SGK3	100
SIK	SIK1	100
SIK2	SIK2	100
SLK	SLK	90
SNARK	NUAK2	64
SNRK	SNRK	90
SRC	SRC	97
SRMS	SRMS	93

SRPK1	SRPK1	92
SRPK2	SRPK2	100
SRPK3	SRPK3	96
STK16	STK16	91
STK33	STK33	90
STK35	STK35	100
STK36	STK36	100
STK39	STK39	61
SYK	SYK	95
TAK1	MAP3K7	100
TAOK1	TAOK1	98
TAOK2	TAOK2	93
TAOK3	TAOK3	90
TBK1	TBK1	86
TEC	TEC	100
TESK1	TESK1	100
TGFBR1	TGFBR1	99
TGFBR2	TGFBR2	100
TIE1	TIE1	100
TIE2	TEK	91
TLK1	TLK1	100
TLK2	TLK2	100
TNIK	TNIK	100
TNK1	TNK1	68
TNK2	TNK2	89
TNNI3K	TNNI3K	91
TRKA	NTRK1	55
TRKB	NTRK2	63
TRKC	NTRK3	100
TRPM6	TRPM6	100
TSSK1B	TSSK1B	100
TSSK3	TSSK3	100
TTK	TTK	100
TXK	TXK	100
TYK2(JH1domain-catalytic)	TYK2	74
TYK2(JH2domain-pseudokinase)	TYK2	96
TYRO3	TYRO3	93
ULK1	ULK1	97
ULK2	ULK2	100
ULK3	ULK3	95
VEGFR2	KDR	96
VPS34	PIK3C3	98

VRK2	VRK2	40
WEE1	WEE1	100
WEE2	WEE2	100
WNK1	WNK1	97
WNK2	WNK2	100
WNK3	WNK3	100
WNK4	WNK4	100
YANK1	STK32A	89
YANK2	STK32B	90
YANK3	STK32C	100
YES	YES1	100
YSK1	STK25	96
YSK4	MAP3K19	100
ZAK	ZAK	93
ZAP70	ZAP70	99

Supplementary Table 2. Summary of lifespan analysis for nematodes and mice. Values of mean lifespan and median lifespan, and numbers of nematodes or mice counted (total, deaths and censored) in each lifespan experiments are shown. See also statistical analysis data (*P* values calculated by two-sided Mantel-CoX) comparing the lifespan of worms or mice between different treatments or genotypes.

Genotypes/Treatments	Mean life span (days)			Median life span (days)			N[c]	N[d]	N[e]	P-value Vs saline control within each genotype (Mantel-CoX)
	Estimated life span ± s.e.m.	95% confidence interval		Estimated life span ± s.e.m.	95% confidence interval					
		Lower bound	Upper bound		Lower bound	Upper bound				
**Fig. 4a**										
N2 + vehicle	19.149 ± 0.280	18.6	19.698	19.000 ± 0.289	18.434	19.566	178	22	200	N/A
N2 + 1 μM Aldometanib	19.335 ± 0.273	18.799	19.87	19.000 ± 0.274	18.462	19.538	186	14	200	N/A
N2 + 2 μM Aldometanib	19.620 ± 0.284	19.063	20.176	19.000 ± 0.289	18.434	19.566	184	16	200	N/A
N2 + 5 μM Aldometanib	20.334 ± 0.333	19.682	20.987	21.000 ± 0.325	20.363	21.637	186	14	200	<0.001
N2 + 10 μM Aldometanib	25.364 ± 0.436	24.509	26.218	25.000 ± 0.473	24.072	25.928	182	18	200	<0.001
N2 + 12.5 μM Aldometanib	24.350± 0.436	23.494	25.205	25.000 ± 0.433	24.151	25.849	187	13	200	<0.001
N2 + 25 μM Aldometanib	22.248 ± 0.380	21.504	22.993	23.000 ± 0.366	22.282	23.718	182	18	200	<0.001
N2 + 50 μM Aldometanib	21.558 ± 0.351	20.87	22.245	21.000 ± 0.362	20.29	21.71	185	15	200	<0.001
**Fig. 4c (left)**										
aak-2 + vehicle	15.658 ± 0.256	15.157	16.159	15.000 ± 0.281	14.449	15.551	166	34	200	N/A
aak-2 + 10 μM Aldometanib	15.692 ± 0.233	15.235	16.149	15.000 ± 0.277	14.457	15.543	172	28	200	N/A
**Fig. 4c (middle)**										
lmtr-2 + vehicle	18.903 ± 0.344	18.228	19.578	19.000 ± 0.518	17.984	20.016	173	27	200	N/A
lmtr-2 + 10 μM Aldometanib	18.698 ± 0.343	18.024	19.371	19.000 ± 0.449	18.12	19.88	174	26	200	N/A
**Fig. 4c (right)**										
axl-1 + vehicle	19.184 ± 0.309	18.578	19.79	19.000 ± 0.366	18.283	19.717	172	28	200	N/A
axl-1 + 10 μM Aldometanib	18.869 ± 0.337	18.209	19.529	19.000 ± 0.568	17.886	20.114	176	24	200	N/A

Genotypes/Treatments	Mean life span (days)			Median life span (days)			N[c]	N[d]	N[e]	P-value Vs saline control within each genotype (Mantel-CoX)
	Estimated life span ± s.e.m.	95% confidence interval		Estimated life span ± s.e.m.	95% confidence interval					
		Lower bound	Upper bound		Lower bound	Upper bound				
**Fig. 4g (Male)**										
Male + vehicle	788.684 ± 11.382	766.376	810.992	796.000 ± 19.615	757.555	834.445	171	-	171	N/A
Male + Aldometanib	841.994 ± 11.236	819.971	864.017	855.000 ± 12.532	830.437	879.563	175	-	175	0.001
**Fig. 4g (Female)**										
Female + vehicle	778.463 ± 16.038	747.029	809.898	800.000 ± 29.682	741.824	858.176	95	-	95	N/A
Female + Aldometanib	855.856 ± 17.104	822.331	889.38	854.000 ± 28.892	797.372	910.628	90	-	90	0.001
**Fig. 4d**										
N2 + vehicle	11.704 ± 0.387	10.946	12.462	12.000 ± 0.465	11.089	12.911	43	17	60	N/A
N2 + 10 μM Aldometanib	13.884 ± 0.460	12.982	14.786	13.000 ± 0.326	12.362	13.638	43	17	60	0.001
N2 + 12.5 μM Aldometanib	14.117 ± 0.506	13.125	15.11	14.000 ± 0.620	12.784	15.216	48	12	60	<0.001

[a]Independent repeats of each life span experiment were performed. Data from representative experiments are shown.

[b]Life span data sets within each panel of this table were done in parallel and statistical analyses was done within the data set.

[c]Number of worms or mice scored (death events).

[d]Number of nematodes or mice censored. See Methods section for detailed information of censored mice.

[e]Total number of nematodes and mice.

Supplementary Table 3. Full list of primers for analysing mRNA levels of mitochondrial genes in mouse and *C. elegans*.

Species	Gene name	Forward primer	Reverse primer
Mouse	*Gapdh*	GACTTCAACAGCAACTCCCAC	TCCACCACCCTGTTGCTGTA
	Nd1	TGCACCTACCCTATCACTCA	CGGCTCATCCTGATCATAGAATGG
	Nd2	ATACTAGCAATTACTTCTATTTTCATAGGG	GAGGGATGGGTTGTAAGGAAG
	Nd3	AAGCAAATCCATATGAATGCGG	GCTCATGGTAGTGGAAGTAGAAG
	Nd4	CCTCAGACCCCCTATCCACA	GTTTGGTTCCCTCATCGGGT
	Nd4l	CCAACTCCATAAGCTCCATACC	GATTTTGGACGTAATCTGTTCCG
	Nd5	ACGAAAATGACCCAGACCTC	GAGATGACAAATCCTGCAAAGATG
	Nd6	TGTTGGAGTTATGTTGGAAGGAG	CAAAGATCACCCAGCTACTACC
	Tfam	GGTCGCATCCCCTCGTCTAT	TTGGGTAGCTGTTCTGTGGAA
	Cs	CTCTACTCACTGCAGCAACCC	TTCATGCCTCTCATGCCACC
	Ndufs8	TGGCGGCAACGTACAAGTAT	GTAGTTGATGGTGGCAGGCT
	Ndufab1	GGACCGAGTTCTGTATGTCTTG	AAACCCAAATTCGTCTTCCATG
	Ndufb10	TGCCAGATTCTTGGGACAAGG	GTCGTAGGCCTTCGTCAAGT
	Ndufv3	GTGTGCTCAAAGAGCCCGAG	TCAGTGCCGAGGTGACTCT
	Ndufa8	GCGGAGCCTTTCACAGAGTA	TCAATCACAGGGTTGGGCTC
	Ndufs3	CTGACTTGACGGCAGTGGAT	CATACCAATTGGCCGCGATG
	Ndufa9	TCTGTCAGTGGAGTTGTGGC	CCCATCAGACGAAGGTGCAT
	Ndufa10	CAGCGCGTGGGACGAAT	ACTCTATGTCGAGGGGCCTT
	Sdha	AGGGTTTAATACTGCATGCCTTA	TCATGTAATGGATGGCATCCT
	Sdhb	AGTGCGGACCTATGGTGTTG	AGACTTTGCTGAGGTCCGTG
	Sdhc	TGAGACATGTCAGCCGTCAC	GGGAGACAGAGGACGGTTTG
	Sdhd	TGGTACCCAGCACATTCACC	GGGTGTCCCCATGAACGTAG
	Cytb	CCCACCCCATATTAAACCCG	GAGGTATGAAGGAAAGGTATTAGGG
	Uqcrc1	ATCAAGGCACTGTCCAAGG	TCATTTTCCTGCATCTCCCG
	Uqcrc2	TTCCAGTGCAGATGTCCAAG	CTGTTGAAGGACGGTAGAAGG
	Atp5f1b	CCGTGAGGGCAATGATTTATAC	GTCAAACCAGTCAGAGCTACC
	Cox6a1	GTTCGTTGCCTACCCTCAC	TCTCTTTACTCATCTTCATAGCCG
	Atp6	TCCCAATCGTTGTAGCCATC	TGTTGGAAAGAATGGAGTCGG
	Atp8	GCCACAACTAGATACATCAACATG	TGGTTGTTAG TGATTTTGGTGAAG
	Atp5f1a	CATTGGTGATGGTATTGCGC	TCCCAAACACGACAACTCC
	Cox1	CCCAGATATAGCATTCCCACG	ACTGTTCATCCTGTTCCTGC
	Cox2	TCTACAAGACGCCACATCCC	ACGGGGTTGTTGATTTCGTCT
	Cox3	CGTGAAGGAACCTACCAAGG	CGCTCAGAAGAATCCTGCAA
	Cox5b	AGCTTCAGGCACCAAGGAAG	TGGGGCACCAGCTTGTAATG
C. elegans	*ama-1*	GACATTTGGCACTGCTTTGT	ACGATTGATTCCATGTCTCG
	nuo-6	CTGCCAGGACATGAATACAATCTGAG	GCTATGAGGATCGTATTCACGACG
	nuaf-1	GAGACATAACGAGGCTCGTGTTG	GAAGCCTTCTTTCCAATCACTATCG
	sdha-1	TTACCAGCGTGCTTTCGGAG	AGGGTGTGGAGAAGAGAATGACC
	sdhb-1	GCTGAACGTGATCGTCTTGATG	GTAGGATGGGCATGACGTGG

	cyc-2.1	CGGAGTTATCGGACGTACATCAG	GTCTCGCGGGTCCAGACG
	isp-1	GCAGAAAGATGAATGGTCCGTTG	ATCCGTGACAAGGGCAGTAATAAC
	cco-1	GCTGGAGATGATCGTTACGAG	GCATCCAATGATTCTGAAGTCG
	cco-2	GTGATACCGTCTACGCCTACATTG	GCTCTGGCACGAAGAATTCTG
	atp-3	GTCCTCGACCCAACTCTCAAG	GTCCAAGGAAGTTTCCAGTCTC
	nduo-1	AGCGTCATTTATTGGGAAGAAGAC	AAGCTTGTGCTAATCCCATAAATGT
	nduo-2	TCTTTGTAGAGGAGGTCTATTACA	ATGTTAAAAACCACATTAGCCCA
	nduo-4	GCACACGGTTATACATCTACACTTATG	GATGTATGATAAAATTCACCAATAAGG
	nduo-5	AGATGAGATTTATTGGGTATTTCTAG	CACCTAGACGATTAGTTAATGCTG
	ctc-1	GCAGCAGGGTTAAGATCTATCTTAG	CTGTTACAAATACAGTTCAAACAAAT
	ctc-2	GTAGTTTATTGTTGGGAGTTTTAGTG	CACAATAATTCACCAAACTGATACTC
	atp-6	TGCTGCTGTAGCGTGATTAAG	ACTGTTAAAGCAAGTGGACGAG
	ctb-1	TGGTGTTACAGGGGCAACAT	TGGCCTCATTATAGGGTCAGC

Supplementary Table 4. Full list of primers for analysing mitochondrial DNA copy numbers in mouse and *C. elegans.*

Species	Gene name	Forward primer	Reverse primer
Mouse	*Hk2*	GCCAGCCTCTCCTGATTTTAGTGT	GGGAACACAAAAGACCTCTTCTGG
	Nd1	CTAGCAGAAACAAACCGGGC	CCGGCTGCGTATTCTACGTT
C.elegans	*nd-1*	AGCGTCATTTATTGGGAAGAAGAC	AAGCTTGTGCTAATCCCATAAATGT
	act-3	TGCGACATTGATATCCGTAAGG	GGTGGTTCCTCCGGAAAGAA

Supplementary Table 5. Full list of primers for analysing mRNA levels of inflammatory and fibrogenic genes in mouse.

Species	Gene name	Forward primer	Reverse primer
Mouse	*Gapdh*	GACTTCAACAGCAACTCCCAC	TCCACCACCCTGTTGCTGTA
	Cxcr2	TGGCTGGGATTCACCTCAAGAACA	TGTGGCTATGACTTCGGTTTGGGT
	Cxcl2	AGTGAACTGCGCTGTCAATG	GAGGGATGGGTTGTAAGGAAG
	Tnf-α	CCCTCACACTCAGATCATCTTCT	GCTACGACGTGGGCTACAG
	Mcp1	CCACTCACCTGCTGCTACTCA	TGGTGATCCTCTTGTAGCTCTCC
	Il-1β	GCAACTGTTCCTGAACTCAACT	ATCTTTTGGGGTCCGTCAACT
	Tgfb	CTCCCGTGGCTTCTAGTGC	GCCTTAGTTTGGACAGGATCTG
	Timp1	GCAACTCGGACCTGGTCATAA	CGGCCCGTGATGAGAAACT
	Col1a1	GCTCCTCTTAGGGGCCACT	CCACGTCTCACCATTGGGG
	Col3a1	CTGTAACATGGAAACTGGGGAAA	CCATAGCTGAACTGAAAACCAC
	Acta2	GTCCCAGACATCAGGGAGTAA	TCGGATACTTCAGCGTCAGGA
	Pdgfa	GAGGAAGCCGAGATACCCC	TGCTGTGGATCTGACTTCGAG
	Pdgfb	CATCCGCTCCTTTGATGATCTT	GTGCTCGGGTCATGTTCAAGT
	Pdgfra	TCCATGCTAGACTCAGAAGTC	TCCCGGTGGACACAATTTTTC
	Ddr2	ATCACAGCCTCAAGTCAGTGG	TTCAGGTCATCGGGTTGCAC

Synthetic procedures for Aldometanib and its derivatives

Unless otherwise noted, reagents and solvents in purest possible forms were obtained from commercial suppliers and were used without further purification. Reactions were monitored by thin-layer chromatography and were visualised with UV. Removal of solvents was conducted by using a rotary evaporator, and the residual solvent was removed from nonvolatile compounds using a vacuum manifold maintained at 1 torr. ^{1}H NMR and ^{13}C NMR spectra were obtained using a Ultrashield Plus-600 (600 MHz) spectrometer (Bruker), and were analysed using MestReNova 9.0 software. Chemical shifts are reported in parts per million (δ) relative to residual undeuterated solvent as an internal reference. Coupling constants (*J*) are reported in hertz. Spin multiplicities are described as s (singlet), brs (broad singlet), t (triplet), q (quartet), and m (multiplet). Mass spectra were recorded on 3100 Mass Detector (Waters). High resolution mass spectra were obtained using Scientific LTQ FTICR-MS (Thermo Fisher), and were analysed using HRMS by Xcalibur 2.2.

Synthetic procedures for Aldometanib

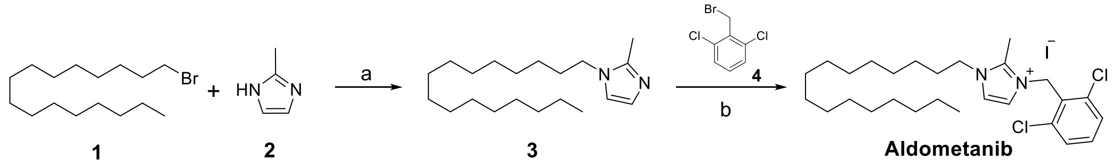

Reagents and conditions: (a) *t*-BuONa, THF, 0 °C to RT; (b) KI, $CHCl_3$, 65 °C

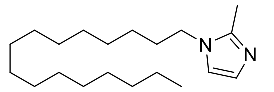

3

1-hexadecyl-2-methyl-1H-imidazole (**3**). The *t*-BuONa (12.96 g, 135 mmol) was added in portions to a stirred suspension of 2-methyl-1H-imidazole (**2**, 11.07 g, 135 mmol, suspended in 250 ml of THF) at 0 °C, followed by addition of 1-bromohexadecane (**1**, 27.48 g, 90 mmol). The mixture was then gradually warmed up to room temperature with continuous stirring. After 24 h, the reaction was quenched by addition of 300 ml of saturated NH_4Cl solution and was extracted with 400 ml of ethyl acetate for three times. The organic layers containing the product (**3**) were combined, and were sequentially washed with water and brine, followed by drying in anhydrous Na_2SO_4. The Na_2SO_4 was then filtered, and the filtrate was evaporated under reduced pressure. The residue containing **3** was dissolved in 150 ml of hexane, and was filtered. The filtrate was then mixed with hexane again, followed by filtration, and the filtrate was concentrated to afford the title compound as colourless liquid (22.78 g, 74.3 mmol, 82.6%). ^{1}H NMR (600 MHz, Chloroform-*d*) δ 6.89 (d, *J* = 1.3 Hz, 1H), 6.80 (d, *J* = 1.3

Hz, 1H), 3.80 (t, J = 7.3 Hz, 2H), 2.37 (s, 3H), 1.71 (p, J = 7.3 Hz, 2H), 1.26 (s, 26H), 0.88 (t, J = 7.0 Hz, 3H). MS (ESI) *m/z*: 306$[M+H]^+$.

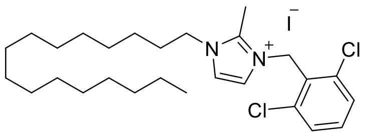

Aldometanib

3-(2,6-dichlorobenzyl)-1-hexadecyl-2-methyl-1H-imidazol-3-ium iodide (LXY-05-029, Aldometanib). The 1-hexadecyl-2-methyl-1H-imidazole (**3**, 22.77 g, 74.3 mmol) was dissolved with 300 ml of $CHCl_3$, and was mixed with 2-(bromomethyl)-1,3-dichlorobenzene (**4**, 19.61 g, 81.7 mmol) and KI (74.01 g, 445.9 mmol) with continuous stirring at 65 °C for 14 h. The mixture then was naturally cooled down to room temperature, and was filtered. The filtrate containing Aldometanib was concentrated and resuspended with hexane, stirred overnight, and was filtered again. The filtrate was mixed with ethyl acetate for crystallisation to afford Aldometanib as colourless crystals (34.64 g, 58.37 mmol, 78.6%). ^{1}H NMR (600 MHz, Chloroform-*d*) δ 7.63 (d, J = 2.2 Hz, 1H), 7.48 – 7.44 (m, 2H), 7.41 (dd, J = 9.3, 6.6 Hz, 1H), 6.96 (d, J = 2.3 Hz, 1H), 5.65 (s, 2H), 4.30 (t, J = 7.5 Hz, 2H), 3.03 (d, J = 2.2 Hz, 3H), 1.90 – 1.84 (m, 2H), 1.36 – 1.19 (m, 26H), 0.88 (t, J = 7.0 Hz, 3H). ^{13}C NMR (150 MHz, $CDCl_3$) δ 144.13, 136.69, 132.14, 129.27, 128.41, 122.31, 120.07, 49.53, 48.27, 31.90, 29.82, 29.68 – 29.50(overlapping), 29.39, 29.34, 29.05, 26.31, 22.66, 14.11, 12.56. MS (ESI) *m/z*: 466$[M]^+$. HRMS (ESI) calculated for $C_{27}H_{43}N_2Cl_2$ $[M]^+$, 465.2798; found, 465.2798.

<u>NMR and HRMS data for Aldometanib derivertives shown in Extended Data Fig. 1c</u>

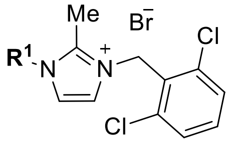

R¹ = C_2H_5– (**1a**), n-C_5H_{11}– (**1b**), n-$C_{10}H_{21}$– (**1c**), n-$C_{17}H_{35}$– (**1d**), n-$C_{22}H_{45}$– (**1e**)

Compound **1a**, ^{1}H NMR (600 MHz, Chloroform-*d*) δ 7.45 (d, J = 7.9 Hz, 2H), 7.41 – 7.36 (m, 2H), 6.92 (s, 1H), 5.53 (s, 2H), 4.41 – 4.09 (m, 2H), 2.84 (s, 3H), 1.49 (d, J = 5.8 Hz, 3H). MS (ESI) *m/z*: 270$[M]^+$. HRMS (ESI) calculated for $C_{13}H_{15}N_2Cl_2$ $[M]^+$, 269.0607; found, 269.0604.

Compound **1b**, ^{1}H NMR (600 MHz, Chloroform-*d*) δ 7.45 (d, J = 8.5 Hz, 2H), 7.39 (d, J = 7.1 Hz, 1H), 7.33 (s, 1H), 6.91 (s, 1H), 5.52 (s, 2H), 4.13 (t, J = 7.3 Hz, 2H), 2.81 (s, 3H), 1.84 – 1.72 (m, 2H), 1.43 – 1.18 (m, 4H), 0.88 (t, J = 7.1 Hz, 3H). MS (ESI) *m/z*: 312$[M]^+$. HRMS (ESI) calculated for $C_{16}H_{21}N_2Cl_2$ $[M]^+$, 311.1076; found, 311.1072.

Compound **1c**, ^{1}H NMR (600 MHz, Chloroform-*d*) δ 7.60 (d, J = 2.3 Hz, 1H), 7.45 (d,

J = 1.9 Hz, 1H), 7.44 (s, 1H), 7.40 (dd, J = 9.3, 6.7 Hz, 1H), 6.95 (d, J = 2.2 Hz, 1H), 5.64 (s, 2H), 4.29 (t, J = 7.5 Hz, 2H), 3.02 (s, 3H), 1.88 – 1.82 (m, 2H), 1.31 – 1.20 (m, 14H), 0.86 (t, J = 7.0 Hz, 3H). MS (ESI) *m/z*: 382 $[M]^+$. HRMS (ESI) calculated for $C_{21}H_{31}N_2Cl_2$ $[M]^+$, 381.1859; found, 381.1855.

Compound **1d**, ^{1}H NMR (600 MHz, DMSO-*d*$_6$) δ 7.71 (d, J = 2.2 Hz, 1H), 7.65 (s, 1H), 7.64 (s, 1H), 7.56 (dd, J = 8.7, 7.5 Hz, 1H), 7.22 (d, J = 2.2 Hz, 1H), 5.57 (s, 2H), 4.15 (t, J = 7.2 Hz, 2H), 2.75 (s, 3H), 1.75 – 1.69 (m, 2H), 1.27 – 1.22 (m, 28H), 0.85 (t, J = 7.0 Hz, 3H). MS (ESI) *m/z*: 480$[M]^+$. HRMS (ESI) calculated for $C_{28}H_{45}N_2Cl_2$ $[M]^+$, 479.2954; found, 479.2950.

Compound **1e**, ^{1}H NMR (600 MHz, DMSO-*d*$_6$) δ 7.71 (d, J = 2.2 Hz, 1H), 7.65 (s, 1H), 7.64 (s, 1H), 7.56 (dd, J = 8.7, 7.5 Hz, 1H), 7.22 (d, J = 2.2 Hz, 1H), 5.57 (s, 2H), 4.16 (t, J = 7.3 Hz, 2H), 2.76 (s, 3H), 1.75 – 1.69 (m, 2H), 1.25 – 1.22 (m, 38H), 0.85 (t, J = 7.0 Hz, 3H). MS(ESI) *m/z*:550 $[M]^+$. HRMS (ESI) calculated for $C_{33}H_{55}N_2Cl_2$ $[M]^+$, 549.3737; found, 549.3733.

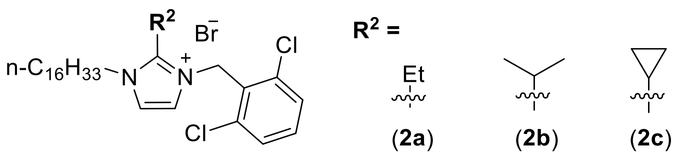

Compound **2a**, ^{1}H NMR (600 MHz, Chloroform-*d*) δ 7.73 (d, J = 2.1 Hz, 1H), 7.46 (d, J = 6.8 Hz, 2H), 7.43 (dd, J = 9.4, 6.4 Hz, 1H), 6.92 (d, J = 2.0 Hz, 1H), 5.66 (s, 2H), 4.31 (t, J = 7.5 Hz, 2H), 3.38 (q, J = 7.6 Hz, 2H), 1.90 (p, J = 7.5 Hz, 2H), 1.43 – 1.16 (m, 29H), 0.87 (t, J = 6.9 Hz, 3H). MS (ESI) *m/z*: 480$[M]^+$. HRMS (ESI) calculated for $C_{28}H_{45}N_2Cl_2$ $[M]^+$, 479.2954; found, 479.2949.

Compound **2b**, ^{1}H NMR (600 MHz, Chloroform-*d*) δ 7.85 (d, J = 2.1 Hz, 1H), 7.49 – 7.43 (m, 3H), 6.76 (d, J = 2.1 Hz, 1H), 5.68 (s, 2H), 4.40 (t, J = 7.7 Hz, 2H), 4.00 (p, J = 7.2 Hz, 1H), 1.89 (p, J = 7.8 Hz, 2H), 1.61 (d, J = 7.2 Hz, 6H), 1.44 – 1.18 (m, 26H), 0.87 (t, J = 7.0 Hz, 3H). MS (ESI) *m/z*: 494$[M]^+$. HRMS (ESI) calculated for $C_{29}H_{47}N_2Cl_2$ $[M]^+$, 493.3111; found, 493.3106.

Compound **2c**, ^{1}H NMR (600 MHz, Chloroform-*d*) δ 7.58 (d, J = 2.2 Hz, 1H), 7.47 (d, J = 7.4 Hz, 2H), 7.41 (dd, J = 9.1, 6.9 Hz, 1H), 6.83 (d, J = 2.2 Hz, 1H), 5.77 (s, 2H), 4.39 (t, J = 7.5 Hz, 2H), 2.19 – 2.08 (m, 1H), 1.92 (p, J = 7.3 Hz, 2H), 1.52 (tt, J = 5.8, 3.3 Hz, 2H), 1.47 (ddd, J = 7.6, 5.6, 3.6 Hz, 2H), 1.40 – 1.20 (m, 26H), 0.88 (t, J = 7.0 Hz, 3H). MS (ESI) *m/z*: 492$[M]^+$. HRMS (ESI) calculated for $C_{29}H_{45}N_2Cl_2$ $[M]^+$, 491.2954; found, 491.2950.
