# Supplementary material for: The aldolase inhibitor aldometanib mimics glucose starvation to activate lysosomal AMPK
Source: Nat Metab. 2022 Oct 10;4(10):1369–401. doi: 10.1038/s42255-022-00640-7 (PMC9584815; doi:10.1038/s42255-022-00640-7)
Supplement: Supplementary file 2 — Reporting Summary [file 42255_2022_640_MOESM2_ESM.pdf]

nature portfolio

Corresponding author(s): Xianming Deng and Sheng-Cai Lin

Last updated by author(s): 2022/8/11

Reporting Summary

Nature Portfolio wishes to improve the reproducibility of the work that we publish. This form provides structure for consistency and transparency in reporting. For further information on Nature Portfolio policies, see our Editorial Policies and the Editorial Policy Checklist.

Statistics

For all statistical analyses, confirm that the following items are present in the figure legend, table legend, main text, or Methods section.

n/a	Confirmed	
☐	☒	The exact sample size (*n*) for each experimental group/condition, given as a discrete number and unit of measurement
☐	☒	A statement on whether measurements were taken from distinct samples or whether the same sample was measured repeatedly
☐	☒	The statistical test(s) used AND whether they are one- or two-sided<br>*Only common tests should be described solely by name; describe more complex techniques in the Methods section.*
☐	☒	A description of all covariates tested
☐	☒	A description of any assumptions or corrections, such as tests of normality and adjustment for multiple comparisons
☐	☒	A full description of the statistical parameters including central tendency (e.g. means) or other basic estimates (e.g. regression coefficient) AND variation (e.g. standard deviation) or associated estimates of uncertainty (e.g. confidence intervals)
☐	☒	For null hypothesis testing, the test statistic (e.g. *F*, *t*, *r*) with confidence intervals, effect sizes, degrees of freedom and *P* value noted<br>*Give P values as exact values whenever suitable.*
☒	☐	For Bayesian analysis, information on the choice of priors and Markov chain Monte Carlo settings
☒	☐	For hierarchical and complex designs, identification of the appropriate level for tests and full reporting of outcomes
☒	☐	Estimates of effect sizes (e.g. Cohen's *d*, Pearson's *r*), indicating how they were calculated

Our web collection on statistics for biologists contains articles on many of the points above.

Software and code

Policy information about availability of computer code

Data collection	EPSON Scan 3.9.3.4 was used to scan blots from X-ray films.<br>Zen 2012, Zen 3.1, Zen Blue 3.3 and LAS X (version 3.0.2.16120) were used to collect microscopic images, as described in related method sections.<br>SoftMax Pro (v.5.4.1.1) and UV Winlab (v.7.1.0.68) were used to collect enzymatic data.<br>LabChart (v.8.1.12) was used to record electrocardiogram, and Vevo 2100 (v.1.6.0) echocardiogram.<br>BM IR software (v7.3.0.28) was used to record surface temperature, and EcoMRI 2018 (v. 180206) body composition.<br>MetaScreen software (v.2.3.15.12) was used to collect data generated from indirect calorimetry.<br>Wave 2.6.1. was used to collect OCR data from MEFs and nematodes, and DatLab software (v.7.3.0.3) muscle tissues.<br>Biacore Insight Evaluation Software (v.3.0.11.15423) was used to analyse SPR data.<br>Capture (v.2021.1.13) was used to record pharyngeal pumping rates.<br>FACSDiva software (v.8.0.2) was used to collect flow cytometry data.<br>Maestro (v.11.9) was used to perform in silico docking.<br>MassLynx 4.1 was used to monitor and collect data from preparative HPLC.<br>TopSpin (version 3.2) was used to collect NMR data for chemicals, and Tune (version 2.1) to HRMS.<br>MassHunter LC/MS acquisition 10.1.48 was used to collect data of FBP concentrations on CE-MS.<br>Analyst TF 1.6 was used to collect data of TAG levels on HPLC-MS, Analyst 1.6.3 for pharmacokinetics of Aldometanib, and Analyst 1.7.1 for metabolites concentrations on HPLC-MS.<br>MassHunter GC/MS acquisition B.07.04.2260 was used to collect data from GC-MS.<br>SC analysis (v.1.0) was used to collect data from in situ FBP binding to aldolase.<br>FACSDiva software (v8.0.2, BD Biosciences) was used to collected data of flow cytometry.
Data analysis	Plots were generated by Prism 9, and were formatted by Illustrator 2022.<br>Statistical analysis was performed by Prism 9 and SPSS 27.0.<br>Intensities of blots and fluorescent dyes were quantified using Image J software (version 1.8.0).

Microscopic images were analysed and processed by Zen 2012 and Zen 3.1, as described in related Methods section, and were formatted on Photoshop 2022.
Enzymatic data were processed and analysed using OriginPro software (v.9.2.0).
Data generated from indirect calorimetry were processed using Macro Interpreter (v.2.32).
SPR data were analysed using Origin 7 software (v.7.0552).
Aimersoft Video Editor software (v.3.6.2.0) was used to analysed pharyngeal pumping rates data.
FlowJo software (v10.6.x) was used to analyse the flow cytometry data.
Results of metabolites measured on CE-MS were analysed on Qualitative Analysis B.06.00, and HPLC-MS on MultiQuant 3.0.3 (for metabolites) or MultiQuant 3.0.3 (for Aldometanib).
Protein and peptide mass spectrometry data were analysed using Peaks Studio (version X+, for timsTOF Pro).
RT-PCR results were analysed by LightCycler software (v.96 1.1).
Preparative HPLC data were analysed by EasyChrom 2.0.
NMR data were analysed on MestReNova 9.0, and on HRMS by Xcalibur 2.2.
OCR results from MEFs and nematodes were analysed by Wave 2.6.1, and muscle tissues Prism 9.
WinNonlin 8.1 was used to calculate the half-life of Aldometanib.
Results of TAG measured on HPLC-MS were analysed by MS-DIAL 4.7, and TCA cycle intermediates by GC-MS MassHunter Workstation Software (version B.07.01SP1, Qualitative Analysis).
FlowJo software (v10.6.x, BD Biosciences) to analyse data of flow cytometry.

For manuscripts utilizing custom algorithms or software that are central to the research but not yet described in published literature, software must be made available to editors and reviewers. We strongly encourage code deposition in a community repository (e.g. GitHub). See the Nature Portfolio guidelines for submitting code & software for further information.

Data

Policy information about availability of data

All manuscripts must include a data availability statement. This statement should provide the following information, where applicable:

- Accession codes, unique identifiers, or web links for publicly available datasets
- A description of any restrictions on data availability
- For clinical datasets or third party data, please ensure that the statement adheres to our policy

The analysis was performed using standard protocols with previously described analysis tools. No custom code was used in this study.
The MS proteomics data have been deposited to the ProteomeXchange Consortium (http://proteomecentral.proteomexchange.org) through the iProX partner repositor with the dataset identifier PXD035968.
Materials, reagents or other experimental data are available upon request.
Full immunoblots are provided as Supplementary Information Fig. 1.
Source data are provided with this paper.

Field-specific reporting

Please select the one below that is the best fit for your research. If you are not sure, read the appropriate sections before making your selection.

☒ Life sciences ☐ Behavioural & social sciences ☐ Ecological, evolutionary & environmental sciences

For a reference copy of the document with all sections, see nature.com/documents/nr-reporting-summary-flat.pdf

Life sciences study design

All studies must disclose on these points even when the disclosure is negative.

Sample size	The chosen sample sizes were similar to those used in this field: n = 4-8 mice or rats were used to determine the effects of AMPK activators (e.g., metformin and MK-8722) on blood glucose (ref. 37,102), fatty liver (ref. 73,75) and NASH (ref. 48,78); n = 100-200 worms and mice were used to determine lifespan (ref. 103-105); n = 9-23 worms and 6-32 mice were used to determine healthspan (ref. 106-108); n = 3-6 mice or rats were used to determine the pharmacokinetics of Aldometanib (ref. 71); n = 18-35 cells from 3-8 dishes/fields were included for conclusions based on immunofluorescent staining (ref. 14,15); n = 3-10 samples were used for evaluation of the levels of metabolites in cells and tissues (ref. 9,14,15); n = 3 samples to determine the expression levels and phosphorylation levels of a specific protein (ref. 13); n = 3 samples to determine the mRNA levels of a specific gene (ref. 13,73); and n = 3-4 samples to determine the activity of aldolase in vitro (ref. 109). No statistical methods were used to predetermine sample size.
Data exclusions	No data was excluded.
Replication	All experimental findings were repeated at least three times as stated in figure legends.
Randomization	Randomisation was applied wherever possible. For example, during MS analyses (metabolites, proteins and pharmacokinetics), samples were processed and subjected to the mass spectrometer in random orders. For animal experiments, sex-matched (only for rodents), age-matched litter-mate animals in each genotype were randomly assigned to pharmacological treatments. In cell experiments, cells of each genotype were parallel seeded and randomly assigned to different treatments. Otherwise, randomisation was not performed. For example, when performing immunoblotting, samples needed to be loaded in a specific order to generate the final figures.
Blinding	Blinding was applied wherever possible. For example, samples, cages or agar plates during sample collection and processing were labelled as code names that were later revealed by the individual who picked and treated animals or cells, but did not participate in sample collection

and processing, until assessing outcome. Similarly, during microscopy data collection and statistical analyses, the fields of view were chosen on a random basis, and are often performed by different operators, preventing potentially biased selection for desired phenotypes. Otherwise, blinding was not performed, such as the measurement of aldolase activity in vitro, as different reagents were added for particular reactions.

Reporting for specific materials, systems and methods

We require information from authors about some types of materials, experimental systems and methods used in many studies. Here, indicate whether each material, system or method listed is relevant to your study. If you are not sure if a list item applies to your research, read the appropriate section before selecting a response.

Materials & experimental systems

n/a	Involved in the study
☐	☒ Antibodies
☐	☒ Eukaryotic cell lines
☒	☐ Palaeontology and archaeology
☐	☒ Animals and other organisms
☒	☐ Human research participants
☒	☐ Clinical data
☒	☐ Dual use research of concern

Methods

n/a	Involved in the study
☒	☐ ChIP-seq
☐	☒ Flow cytometry
☒	☐ MRI-based neuroimaging

Antibodies

Antibodies used

Rabbit anti-phospho-AMPKα-T172 [cat. #2535, 1:1,000 for IB (immunoblotting)], anti-AMPKα (cat. #2532, 1:1,000 for IB), anti-phospho-ACC-Ser79 (cat. #3661, 1:1,000 for IB), anti-ACC (cat. #3662, 1:1,000 for IB), anti-ALDOA (cat. #8060, 1:2,000 for IB), anti-AXIN1 (cat. #2074, 1:1,000 for IB), anti-LAMTOR1 (cat. #8975, 1:500 for IB), anti-phospho-p70 S6K-S389 (cat. #9234, 1:1,000 for IB), anti-p70 S6K (cat. #2708, 1:1,000 for IB), anti-phospho-SREBP1c-S372 (cat. #9874, 1:500 for IB), anti-phospho-TBC1D1-S660 (cat. #6928, 1:500 for IB), anti-TBC1D1 (cat. #4629, 1:1,000 for IB), anti-ATG5 (cat. #12994, 1:1,000 for IB), anti-p62 (cat. #23214, 1:1,000 for IB), anti-SDHA (cat. #11998, 1:1,000 for IB), and anti-COX4 (cat. #4850, 1:1,000 for IB) antibodies were purchased from Cell Signaling Technology. Rabbit anti-transferrin (cat. ab1223, 1:500 for IB), anti-ATP6V1B2 (cat. ab73404, 1:2,000 for IB), anti-UCP1 (cat. ab10983, 1:1,000 for IB), anti-VDAC1 (cat. ab34726, 1:1,000 for IB), anti-LONP1 (cat. ab103809, 1:1,000 for IB), anti-F4/80 [cat. ab111101, 1:250 for immunohistochemistry (IHC)], mouse anti-total OXPHOS (cat. ab110413, 1:1,000 for IB homogenates of MEFs, and 1:5,000 for adipose tissues), rat anti-LAMP2 [cat. ab13524; 1:1,000 for IB or 1:120 IF (immunofluorescent staining)], and HRP-conjugated goat anti-Rat IgG (cat. ab7097, 1:2,000 for IB) antibodies were purchased from Abcam. Rabbit anti-ALDOB (cat. 18065-1-AP, 1:1,000 for IB), anti-HSP60 (cat. 15282-1-AP, 1:1,000 for IB), and anti-tubulin (cat. #10068-1-AP, 1:1,000 for IB nematode tubulin; and cat. #66031-1-Ig, 1:20,000 for IB mammalian tubulin) antibodies were purchased from Proteintech. Goat anti-AXIN [cat. sc-8567, 1:100 for IP (immunoprecipitation) and 1:120 for IF], rabbit anti-SREBP1 (cat. sc-366, 1:1,000 for IB), mouse anti-HA (cat. sc-7392, 1:1,000 for IB), and mouse anti-goat IgG-HRP (cat. sc-2354, 1:5,000 for IB) antibodies were purchased from Santa Cruz Biotechnology. Mouse anti-ALDOC (cat. AM2215b, 1:2,000 for IB) antibody was purchased from Abgent. Normal rabbit control IgG (cat. CR1, 1:100 for IP) was purchased from Sino Biological. The HRP-conjugated goat anti-mouse IgG (cat. 115-035-003, 1:5,000 for IB) and goat anti-rabbit IgG (cat. 111-035-003, 1:5,000 for IB and 1:120 for IHC) antibodies were purchased from Jackson ImmunoResearch. Alexa Fluor 488 donkey anti-goat IgG (cat. A11055, 1:100 for IF), Alexa Fluor 594 donkey anti-rat IgG (cat. A21209, 1:100 for IF) and Alexa Fluor 488 goat anti-rabbit IgG (cat. A11008, 1: 100 for IHC) antibodies were purchased from Thermo.

Validation

Mouse anti-ALDOC (cat. AM2215b) was validated by the manufacturer: https://www.abcepta.com/products/search/AM2215b, and also in our previous study (ref. 14).

The following commercially available antibodies were validated by the company, as well as other researchers (as the information collected by the RRID database):
Rabbit anti-phospho-AMPKα-T172 (cat. #2535, RRID: AB_331250), anti-AMPKα (cat. #2532, RRID: AB_330331), anti-phospho-ACC-Ser79 (cat. #3661, RRID: AB_330337), anti-ACC (cat. #3662, RRID: AB_2219400), anti-ALDOA (cat. #8060, RRID: AB_2797635), anti-AXIN1 (cat. #2074, RRID: AB_2062419), anti-LAMTOR1 (cat. #8975, RRID: AB_10860252), anti-phospho-p70 S6K-S389 (cat. #9234, RRID: AB_2269803), anti-p70 S6K (cat. #2708, RRID: AB_390722), anti-phospho-SREBP1c-S372 (cat. #9874, RRID: AB_10949508), anti-phospho-TBC1D1-S660 (cat. #6928, RRID: AB_10831836), and anti-TBC1D1 (cat. #4629, RRID: AB_1904162) anti-ATG5 (cat. #12994, 1:1,000 for IB, RRID: AB_2630393), anti-p62 (cat. #23214, 1:1,000 for IB, RRID: AB_2798858), anti-SDHA (cat. #11998, 1:1,000 for IB, RRID: AB_2750900), and anti-COX4 (cat. #4850, 1:1,000 for IB, RRID: AB_2085424) antibodies were purchased from Cell Signaling Technology. Rabbit anti-transferrin (cat. ab1223, RRID: AB_298951), anti-ATP6V1B2 (cat. ab73404, RRID: AB_1924799), anti-UCP1 (cat. ab10983, 1:1,000 for IB, RRID: AB_2241462), anti-VDAC1 (cat. ab34726, 1:1,000 for IB, RRID:AB_778788), anti-LONP1 (cat. ab103809, 1:1,000 for IB, RRID:AB_10858161), anti-F4/80 [cat. ab111101, 1:250 for immunohistochemistry (IHC), RRID: AB_10859466], rat anti-LAMP2 (cat. ab13524, RRID: AB_369111), and HRP-conjugated goat anti-Rat IgG (cat. Ab7097, RRID: AB_955411) antibodies were purchased from Abcam. Rabbit anti-ALDOB (cat. 18065-1-AP, RRID: AB_2273968), anti-HSP60 (cat. 15282-1-AP, 1:1,000 for IB, RRID: AB_2121440),and anti-tubulin (cat. #10068-1-AP, RRID: AB_2303998; and cat. #66031-1-Ig, RRID: AB_11042766) antibodies were purchased from Proteintech. Goat anti-AXIN (cat. sc-8567, RRID: AB_2227789), rabbit anti-SREBP1 (cat. sc-366, RRID: AB_2194229), mouse anti-HA (cat. sc-7392, RRID: AB_627809), and mouse anti-goat IgG-HRP (cat. sc-2354, RRID: AB_628490) antibodies were purchased from Santa Cruz Biotechnology. Mouse anti-ALDOC (cat. AM2215b, 1:2,000 for IB) antibody was purchased from Abgent. Normal rabbit control IgG (cat. CR1, 1:100 for IP, RRID: AB_2613396) was purchased from Sino Biological. The HRP-conjugated goat anti-mouse IgG (cat. 115-035-003, RRID: AB_10015289) and goat anti-rabbit IgG (cat. 111-035-003, RRID: AB_2313567) antibodies were purchased from Jackson ImmunoResearch. Alexa Fluor 488 donkey anti-goat IgG (cat. A11055, RRID: AB_2534102) and Alexa Fluor 594 donkey anti-rat IgG (cat. A21209, RRID: AB_2535795) antibodies were

purchased from Thermo.

Eukaryotic cell lines

Policy information about cell lines

Cell line source(s)	HEK293T cells were purchased from ATCC. MEFs and primary hepatocytes were obtained from the indicated mouse strains.
Authentication	HEK293T cells were obtained from and pre-authenticated by ATCC by STR sequencing and used at low passages.
Mycoplasma contamination	The cell lines were routinely tested negative for mycoplasma contamination in our lab.
Commonly misidentified lines (See ICLAC register)	No commonly misidentified lines were used.

Animals and other organisms

Policy information about studies involving animals; ARRIVE guidelines recommended for reporting animal research

Laboratory animals

Animal maintenance:
Mice (3-4 per 385 × 210 × 174 mm3 cage) and rats (2 per 482 × 336 × 268 mm3 cage) were housed with free access to water and standard diet [65% (w/w) carbohydrate, 11% (w/w) fat, and 24% (w/w) protein]. The light was on from 8 a.m. to 8 p.m., with the temperature kept at 21-24 °C and humidity at 40-70%. Except those used for determining lifespans, only male mice and rats were used in the study. Littermate controls were used throughout the study. Aldometanib was supplied either through oral gavaging or in drinking water. For determining the acute, glucose-lowering effects of Aldometanib on normoglyceamic (lean) rodents, 4-week-old C57BL/6 mice or ZDF rats were used. For creating the diabetic mouse model, C57BL/6 mice were fed with HFD (high fat diet, with 60% calories from fat, cat. D12492, Research Diets) for 4 months starting at 4 weeks old, and db/db mice with standard diet for 3 months starting at 4 weeks old. For creating the diabetic rat model, ZDF rats were fed with HFD for 3 months starting at 8 weeks old. For creating the NASH model, C57BL/6 mice were fed with AMLN diet [40% calories from fat, 20% calories from fructose, and 2% (w/w) cholesterol, cat. D09100301, Research Diets] for 30 weeks starting at 4 weeks old. In this study, only male rodents were used (except that in Fig. 8h, both male and female mice were used). Unless stated otherwise (e.g., aged mice for lifespan determination), rodents at 4 weeks old were used.
Nematodes (hermaphrodites) were maintained on nematode growth medium (NGM) plates [1.7% (w/v) agar, 0.3% (w/v) NaCl, 0.25% (w/v) bacteriological peptone, 1 mM CaCl2, 1 mM MgSO4, 25 mM KH2PO4-K2HPO4, pH 6.0, 0.02% (w/v) streptomycin, and 5 µg/ml cholesterol] spread with E. coli OP50 as standard food. Aldometanib of desired concentrations was added to the autoclaved NGM (cooled down to 60 °C) before pouring onto pates. All worms were cultured at 20 °C. Unless stated otherwise, nematodes at L4 stage were used.

Animal source:
Wildtype C57BL/6J mice (#000664) were obtained from The Jackson Laboratory. AXINF/F and LAMTOR1F/F mice were generated and maintained as described previously (ref. 13). TRPV1-/- mice were obtained from The Jackson Laboratory provided by Dr. David Julius (#003770). TRPV1-/- mice with knockdown of TRPV2-4 or GFP were generated as described previously (ref. 15). AMPKα1F/F (Jackson Laboratory, #014141) and AMPKα2F/F mice (Jackson Laboratory, #014142) were obtained from Jackson Laboratory, provided by Dr. Sean Morrison. CaMKK2-/- mice (MGI: 4941485) were obtained from Jackson Laboratory, provided by Dr. Talal Chatila. Db/db (Leprdb) mice were obtained from Jackson Laboratory (#000697). LKB1F/F mice (MGI: 2387402) were obtained from Frederick National Laboratory for Cancer Research, provided by Dr. Ron DePinho (BRC no. RBRC02975). ATG5F/F mice were obtained from RIKEN, provided by Dr. N. Mizushima (BRC no. RBRC02975). LAMTOR1F/F and AMPKα1/2F/F mice were crossed with Mck-Cre or Alb-Cre mice to generate muscle-specific knockout (LAMTOR1-MKO) or liver-specific LAMTOR1 knockout (LAMTOR1-LKO) mice, and muscle-specific (AMPKα-MKO) or liver-specific AMPKα knockout (AMPKα-LKO) mice. AMPKα1/2F/F were also crossed with Alb-CreERT2 mice to generate inducible liver-specific AMPKα knockout mice (for deleting AMPK in established fatty liver and NASH mouse models to analyse the roles of AMPK, after activation by Aldometanib, in fatty liver or NASH alleviation), in which hepatic AMPKα were deleted by intraperitoneally injecting the Alb-CreERT2-carrying AMPKα1/2F/F mice [fed with HFD for 16 weeks, or AMLN (Amylin liver NASH model diet) diet for 30 weeks] with tamoxifen (dissolved in corn oil) at 200 mg/kg, 3 times a week. Knockout efficiencies were analysed at 1 week after the last injection by western blotting. All mouse strains used in this study are in the C57BL/6J background. ZDF rats (#123) were obtained from Vital River Laboratory Animal Technology (Beijing, China), a branch of Charles River Laboratories.
Wildtype (N2 Bristol) and aak-2(ok524) strains were obtained from Caenorhabditis Genetics Center, and the aak-2(ok524) strain was outcrossed 6 times to N2 before the experiments. The axl-1, lmtr-2, HLH-30::GFP and hsp-6p::gfp strains that have been outcrossed 6 times to N2, as described previously (ref. 118), were gifts from Dr. Ying Liu from the Institute of Molecular Medicine, Peking University.

Wild animals	The study did not involve wild animals
Field-collected samples	The study did not involve samples collected from the field.
Ethics oversight	Protocols for all rodent experiments were approved by the Institutional Animal Care and the Animal Committee of Xiamen University (XMULAC20180028).

Note that full information on the approval of the study protocol must also be provided in the manuscript.

Flow Cytometry

Plots

Confirm that:

☒ The axis labels state the marker and fluorochrome used (e.g. CD4-FITC).

☒ The axis scales are clearly visible. Include numbers along axes only for bottom left plot of group (a 'group' is an analysis of identical markers).

☒ All plots are contour plots with outliers or pseudocolor plots.

☒ A numerical value for number of cells or percentage (with statistics) is provided.

Methodology

Sample preparation	MEFs grown to 70-80% confluence in a well of a 6-well dish were incubated in 2 ml of DMEM containing 1 mg/ml (final concentration) FITC-dextran for 2 or 4 h at 37 °C in a humidified incubator containing 5% CO2. Cells were then washed with 3 ml of DMEM (pre-heated to 37 °C) for 3 times, and then trypsinised. Some 1 × 106 of trypsinised cells were resuspended with 0.5 ml of PBS supplemented with 2% (m/v) BSA, and were filtered by passing through a 100-µm Cell Strainer.
Instrument	LSRFortessa Cell Analyzer (BD Biosciences), equipped with 20 detectors and 355-nm (15 mW), 405-nm (50 mW), 488-nm (50 mW), and 633 nm (40-mW) lasers. In this study, the 488-nm (50 mW) laser and the 530/30 filter used to excite and detect the fluorescence of FITC-dextran.
Software	FACSDiva software (v8.0.2, BD Biosciences) was used to collected data, the FlowJo software (v10.6.x, BD Biosciences) to analyse data.
Cell population abundance	Cell population abundance was labelled in Extended Data Fig. 3n, a representative analysis.
Gating strategy	Gating strategies used for analytical samples are shown in Extended Data Fig. 3n. Gating strategies, including the FSC-SSC gate, are directly shown in the specific panels. Gate boundaries were set either based on control samples, or followed density distributions based on best practices.

☒ Tick this box to confirm that a figure exemplifying the gating strategy is provided in the Supplementary Information.
