# Supplementary figures and images for: The aldolase inhibitor aldometanib mimics glucose starvation to activate lysosomal AMPK
Source: Nat Metab. 2022 Oct 10;4(10):1369–401. doi: 10.1038/s42255-022-00640-7 (PMC9584815; doi:10.1038/s42255-022-00640-7)

**Fig. 1f**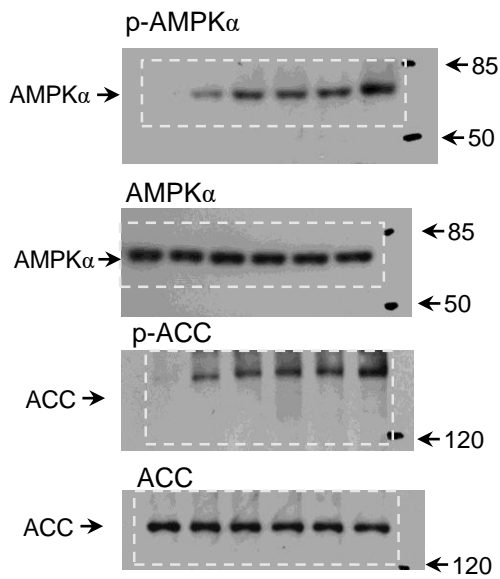**Fig. 1g**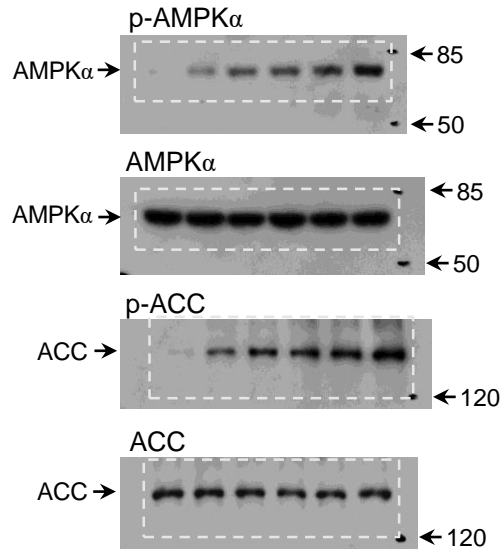**Fig. 1h**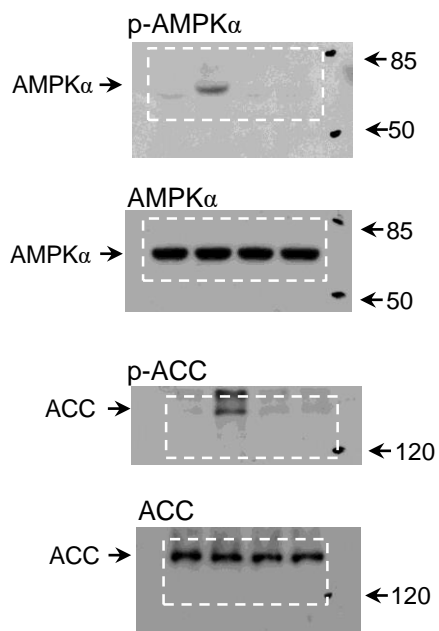**Fig. 1i**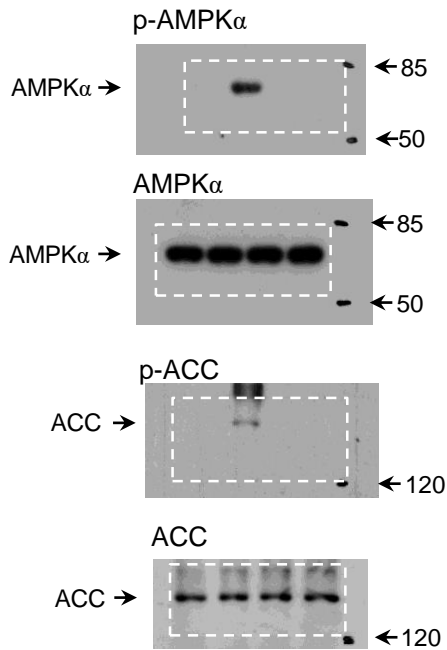

**Fig. 1m**

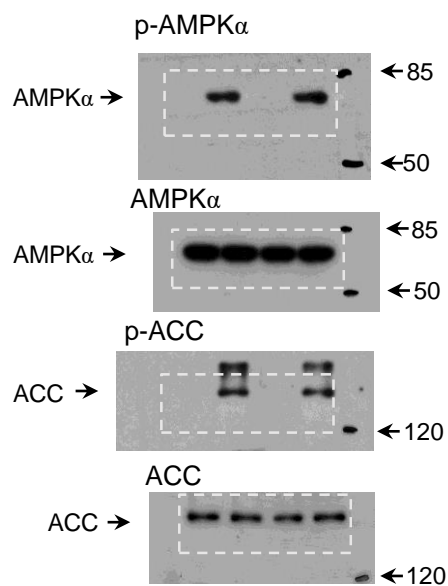

**Fig. 1n**

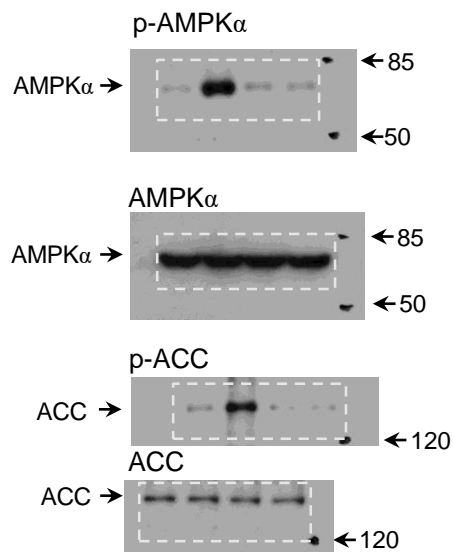

**Fig. 1o**

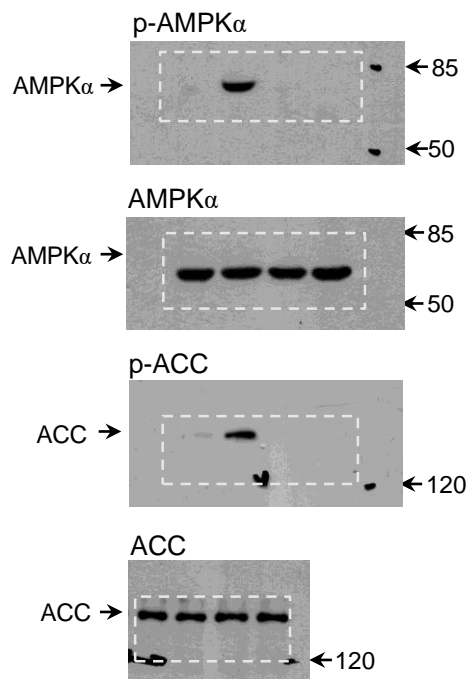

**Fig. 1p**

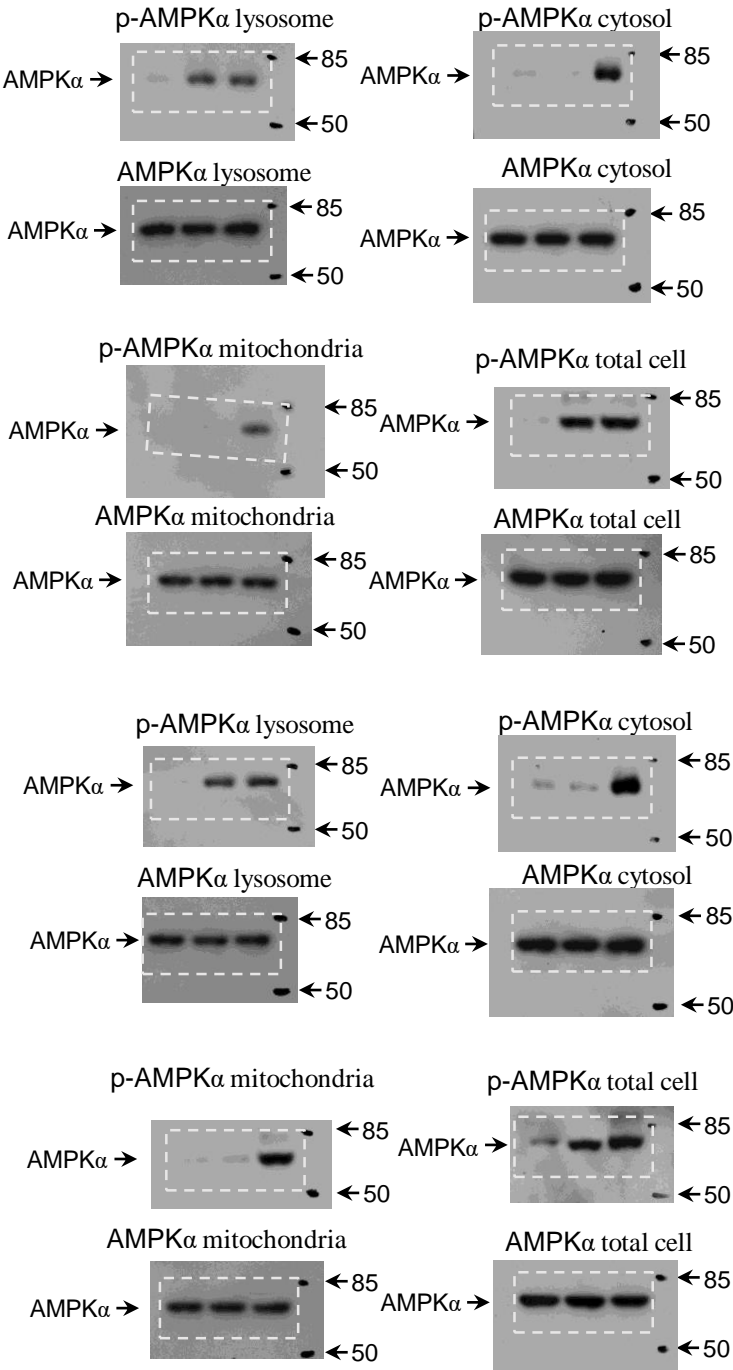

Supplement: Source Data Fig. 1 — Unprocessed western blots. [file 42255_2022_640_MOESM4_ESM.pdf]

**Fig. 2b**

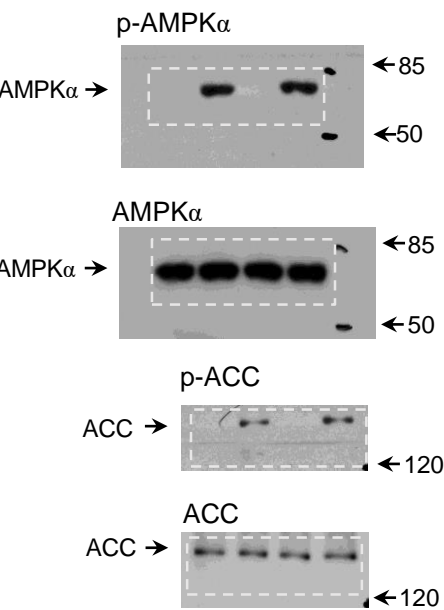

**Fig. 2c**

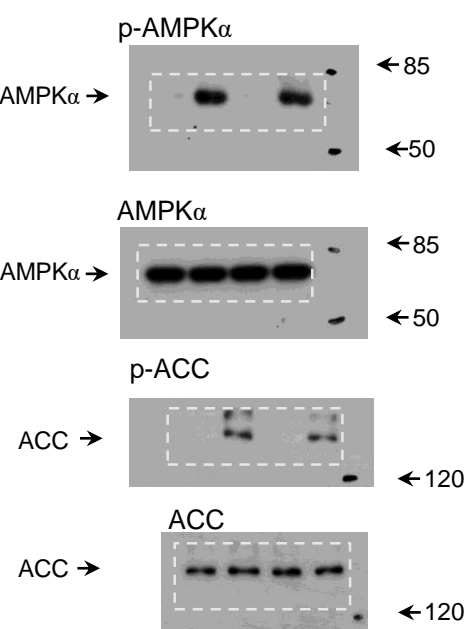

**Fig. 2e**

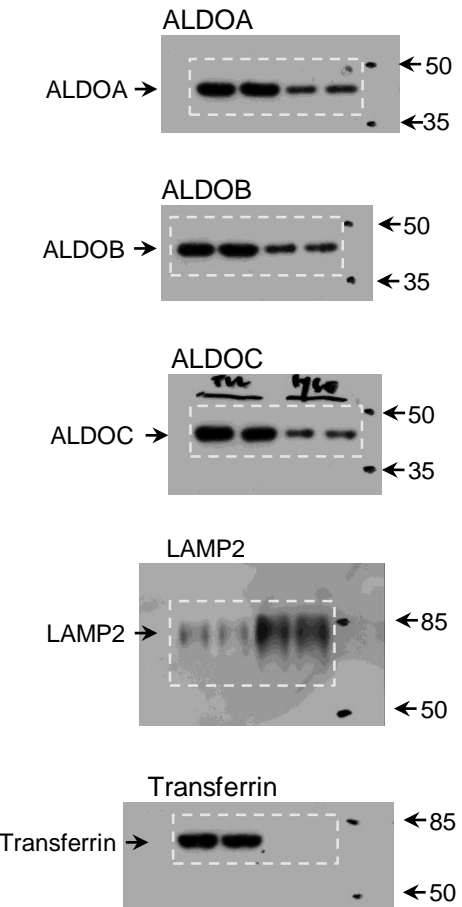

**Fig. 2f**

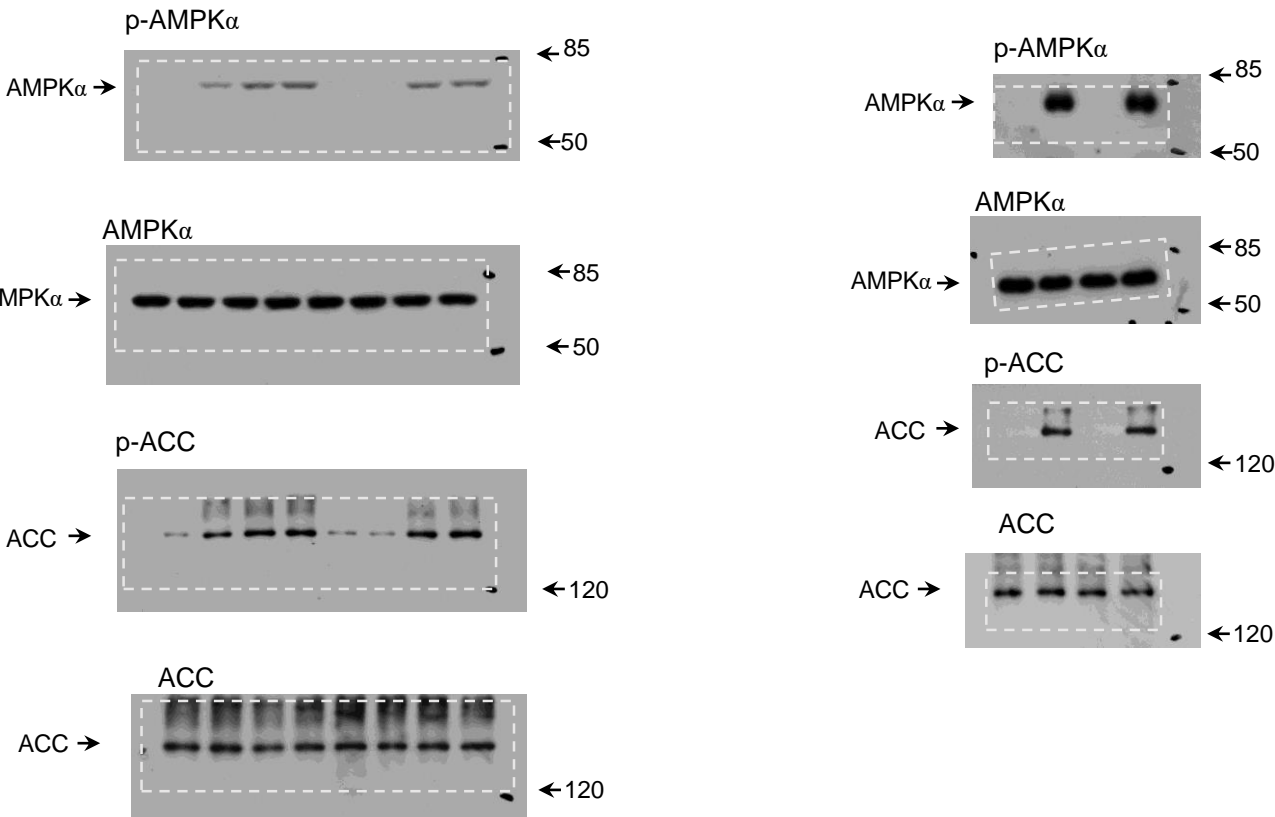

**Fig. 2h**

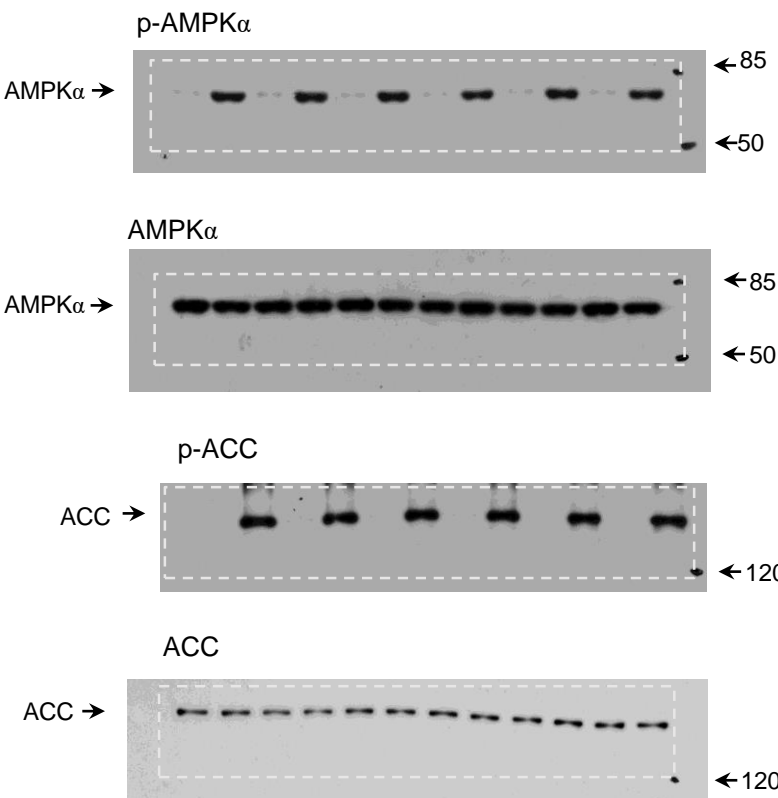

**Fig. 2m**

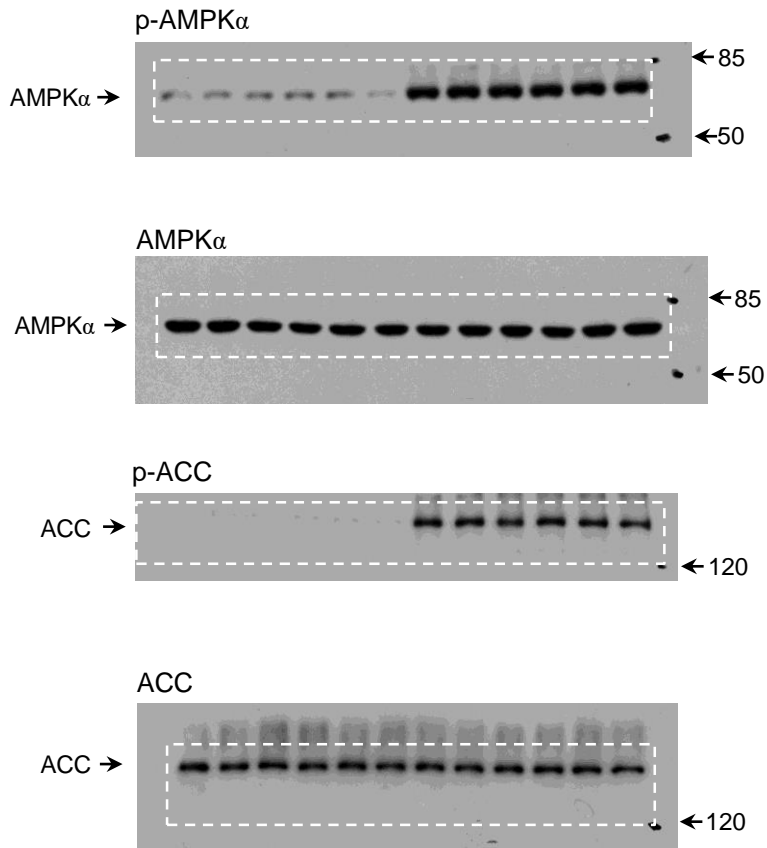

**Fig. 2n**

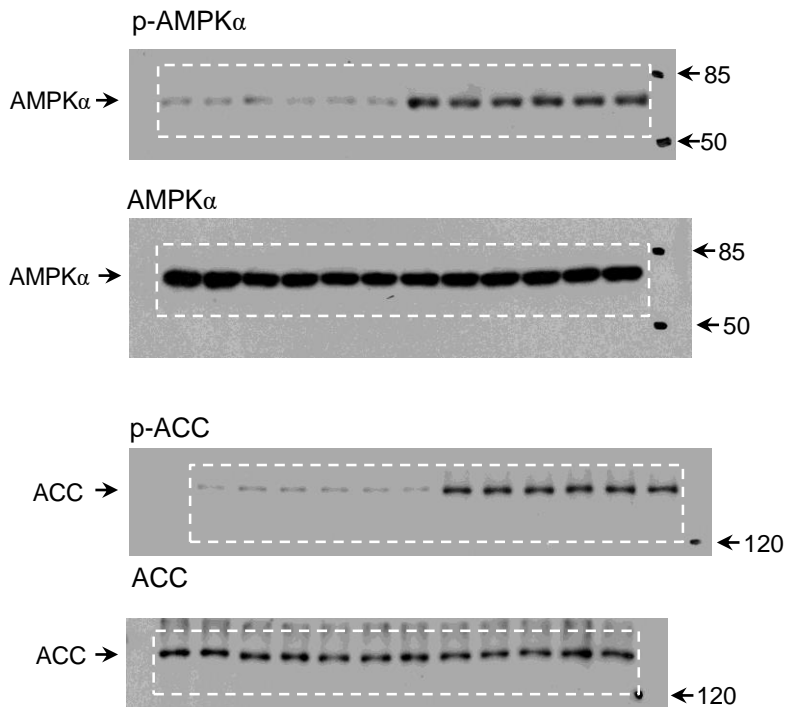

**Fig.2o**

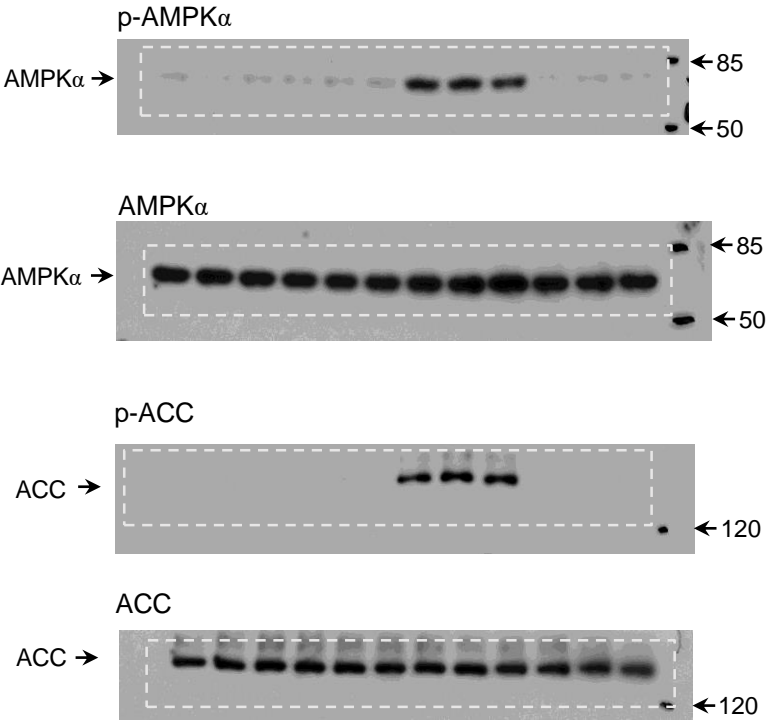

**Fig. 2p**

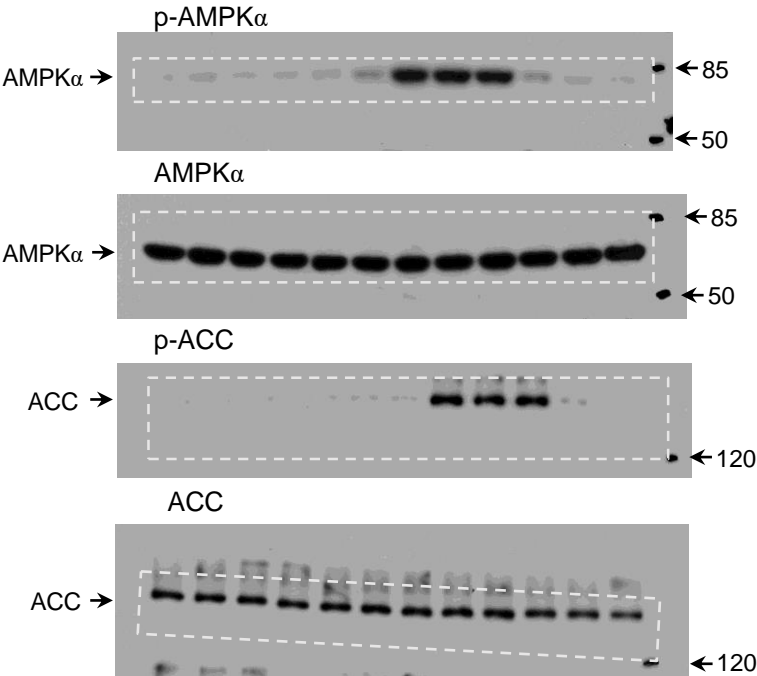

Supplement: Source Data Fig. 2 — Unprocessed western blots. [file 42255_2022_640_MOESM6_ESM.pdf]

**Fig.3d**

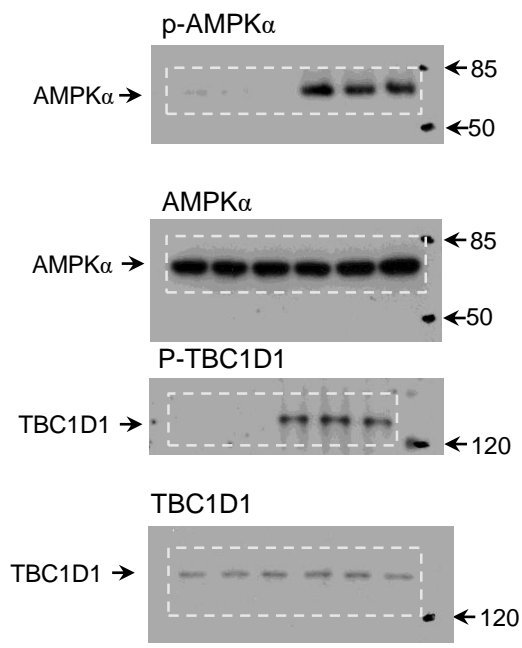

Supplement: Source Data Fig. 3 — Unprocessed western blots. [file 42255_2022_640_MOESM8_ESM.pdf]

**Fig.5a**

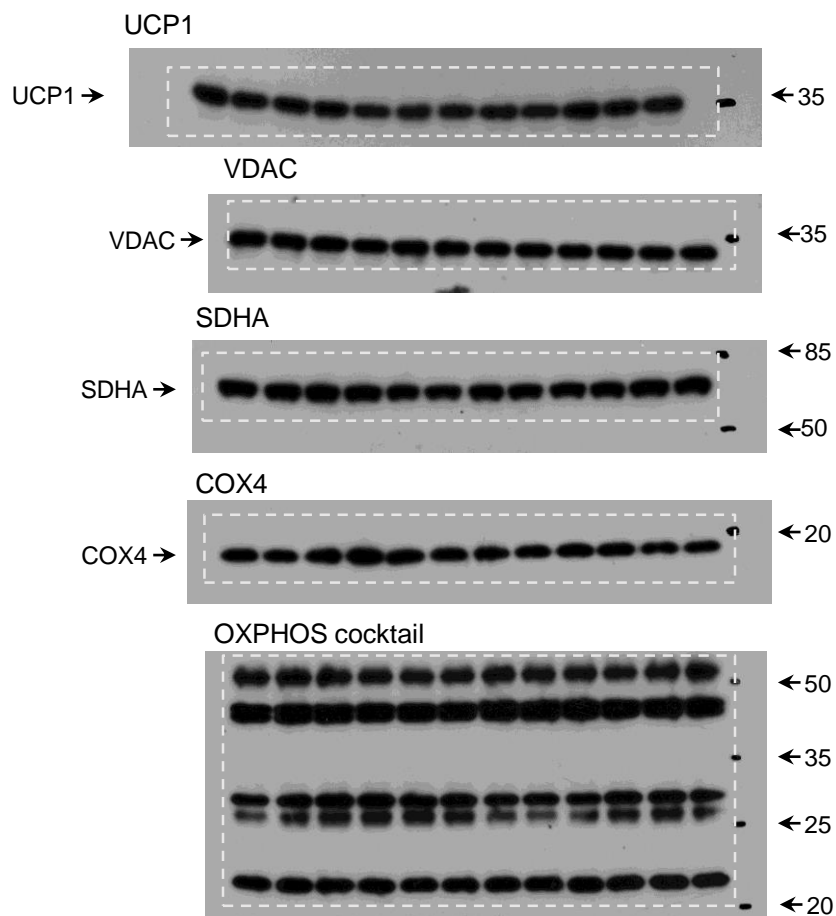

**Fig.5a**

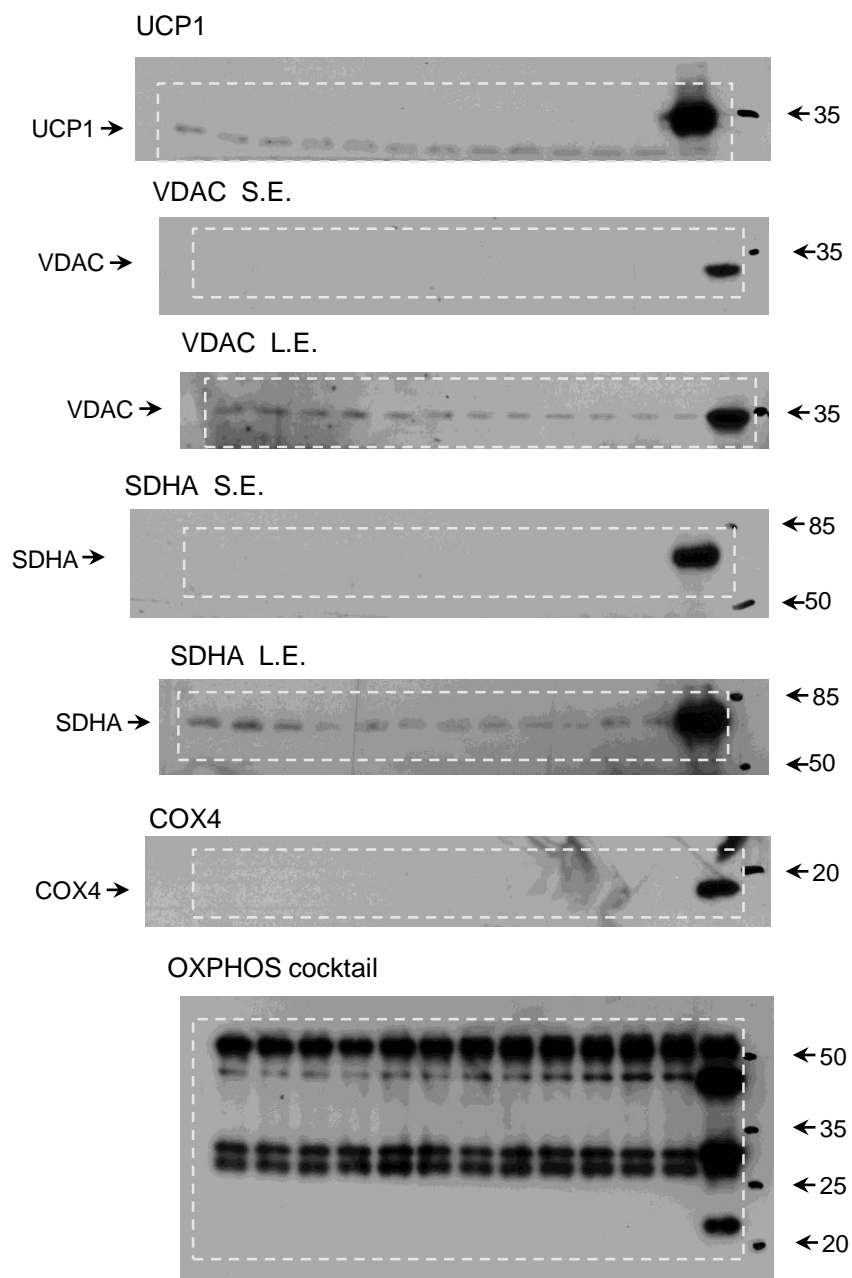

Supplement: Source Data Fig. 5 — Unprocessed western blots. [file 42255_2022_640_MOESM11_ESM.pdf]

Fig.6c

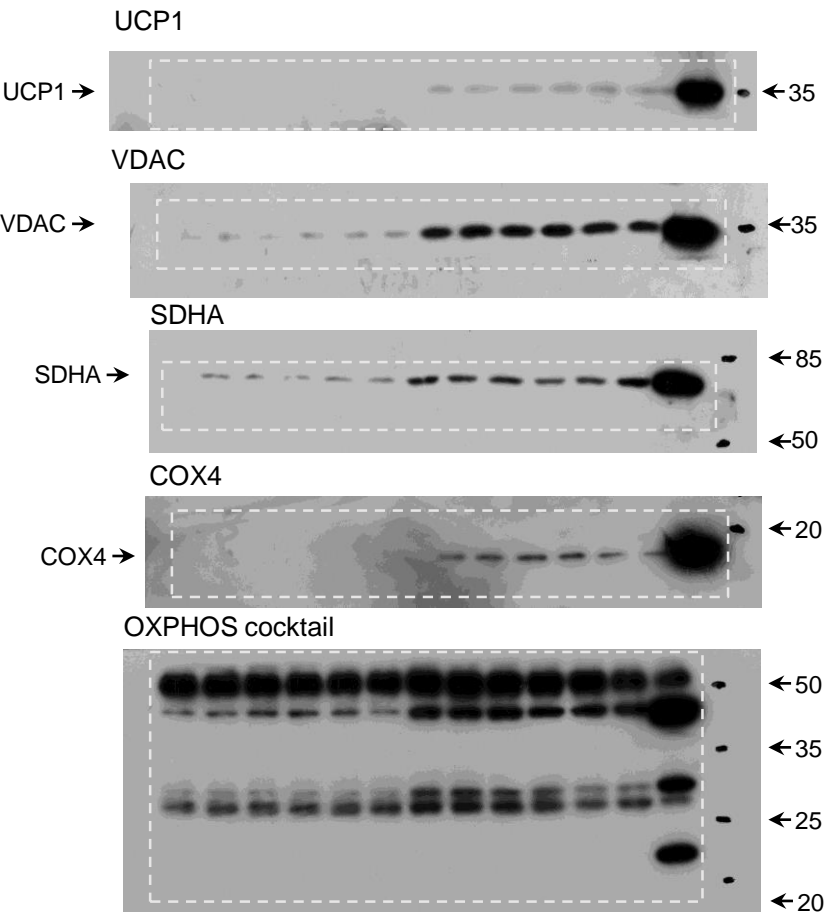

Supplement: Source Data Fig. 6 — Unprocessed western blots. [file 42255_2022_640_MOESM13_ESM.pdf]

Extended Data Fig. 6k

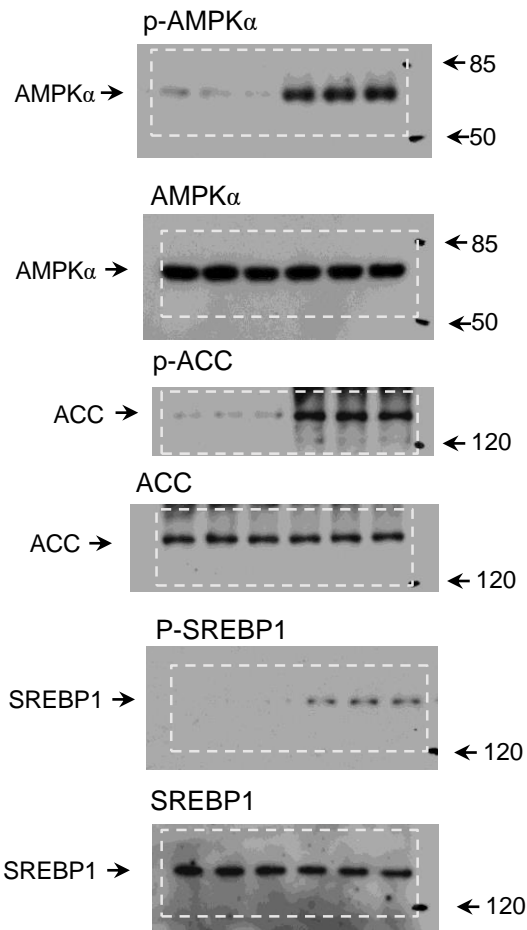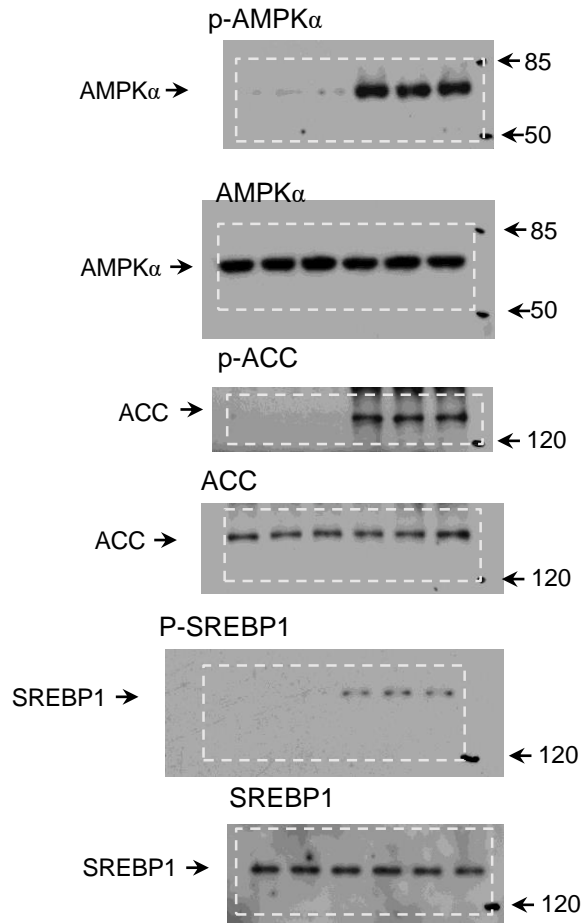

Extended Data Fig. 6k

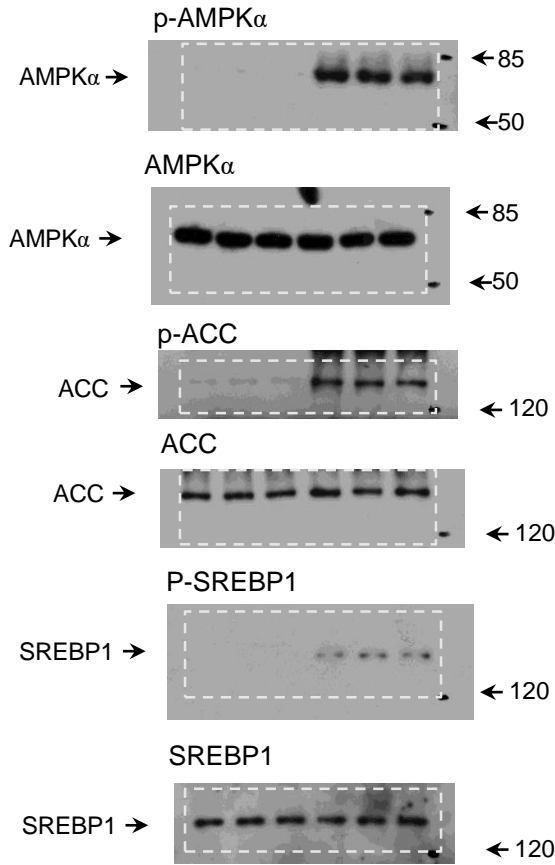

Extended Data Fig. 6n

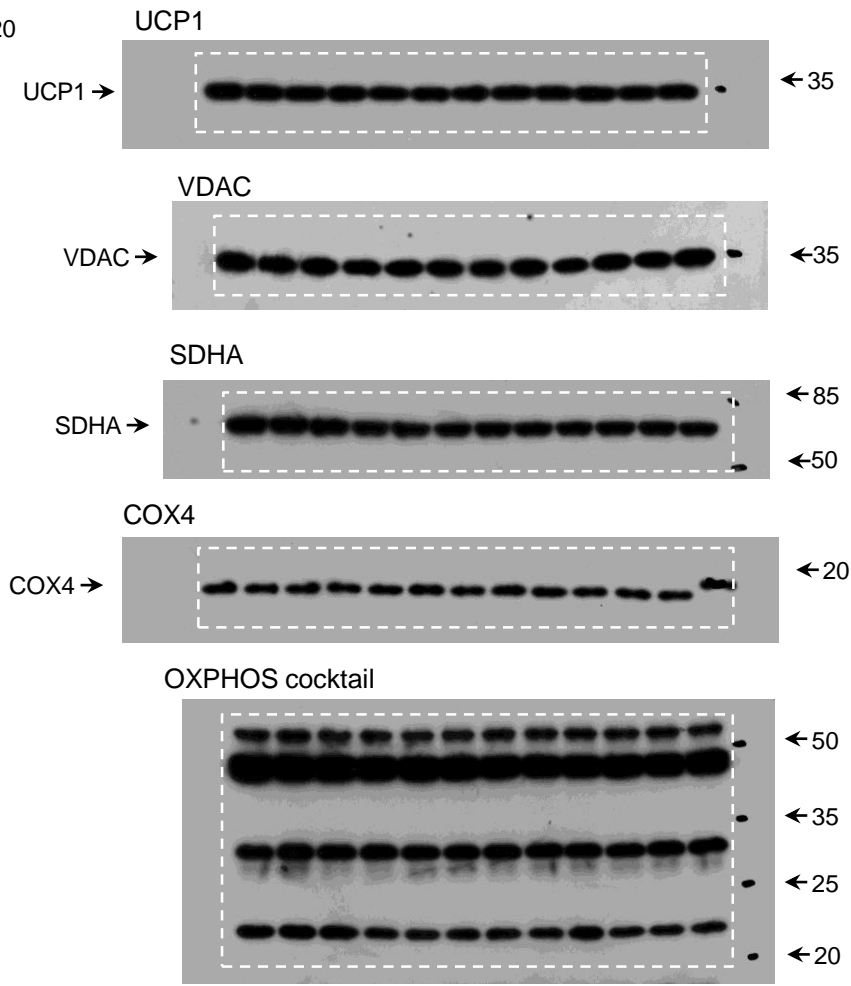

Supplement: Source Data Extended Data Fig. 6 — Unprocessed western blots. [file 42255_2022_640_MOESM25_ESM.pdf]

## Extended Data Fig. 7f

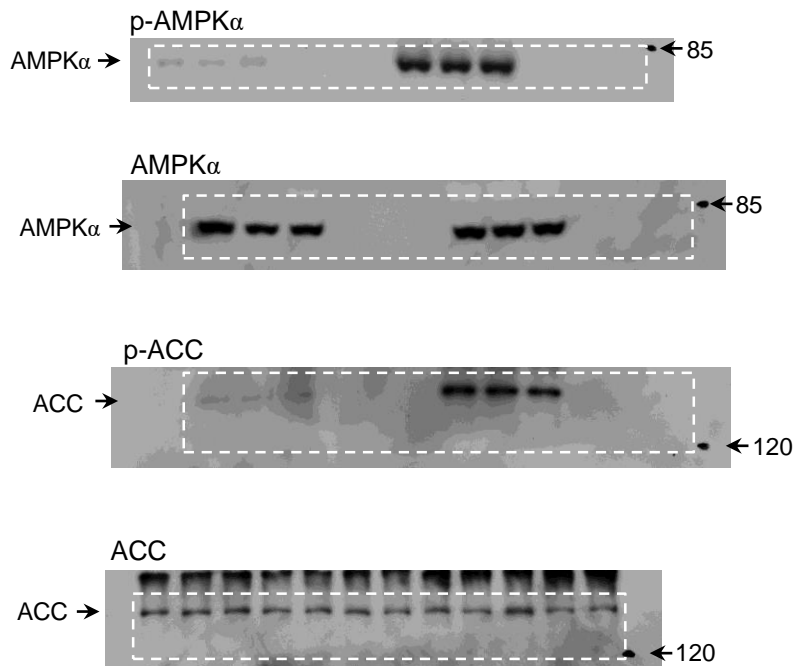

## Extended Data Fig. 7i

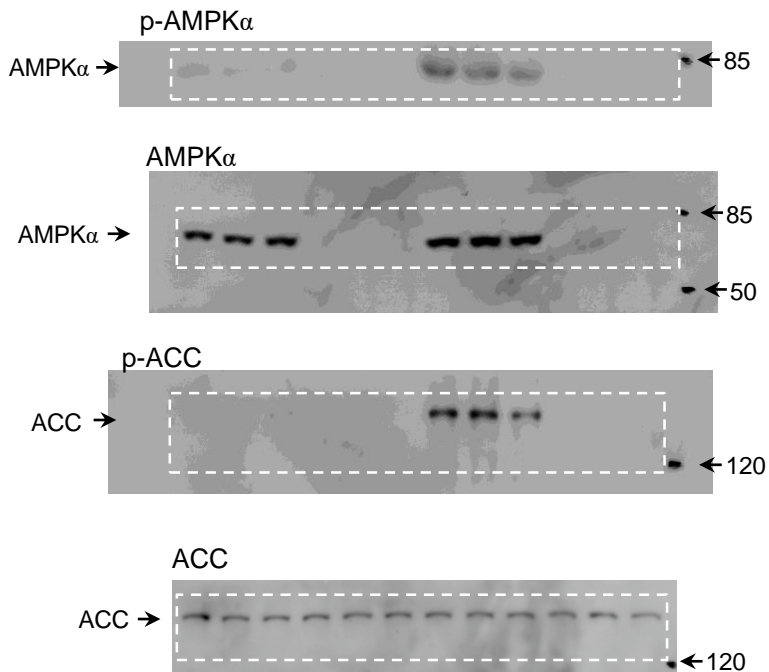

Supplement: Source Data Extended Data Fig. 7 — Unprocessed western blots. [file 42255_2022_640_MOESM27_ESM.pdf]

# Extended Data Fig. 8a

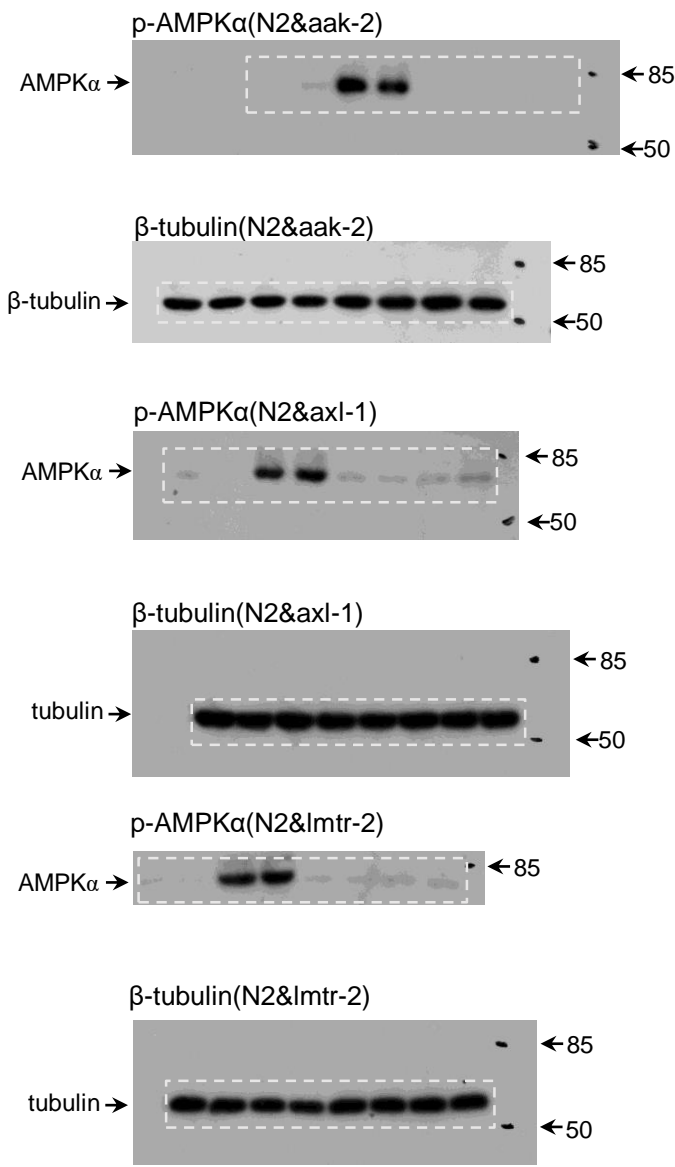

Supplement: Source Data Extended Data Fig. 8 — Unprocessed western blots. [file 42255_2022_640_MOESM29_ESM.pdf]

# Extended Data Fig. 9a

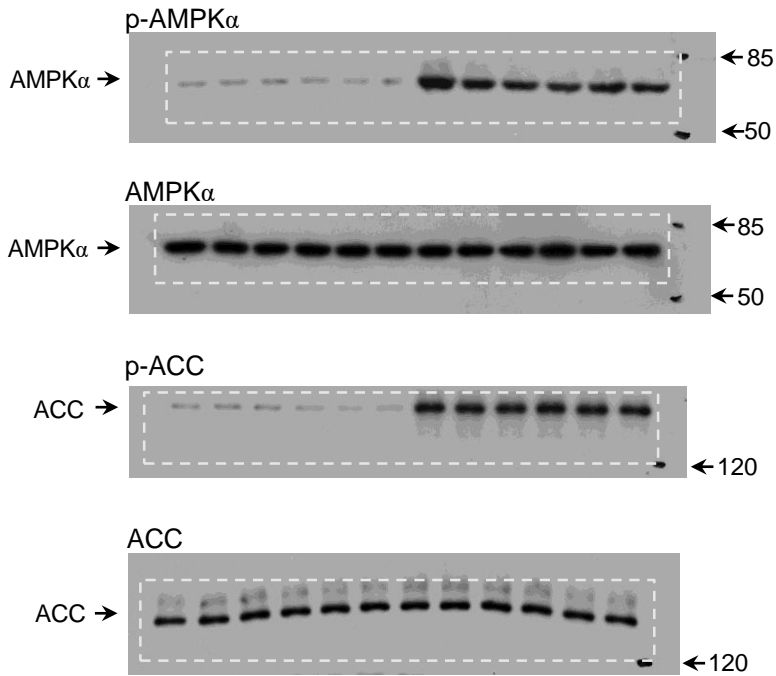

# Extended Data Fig. 9f

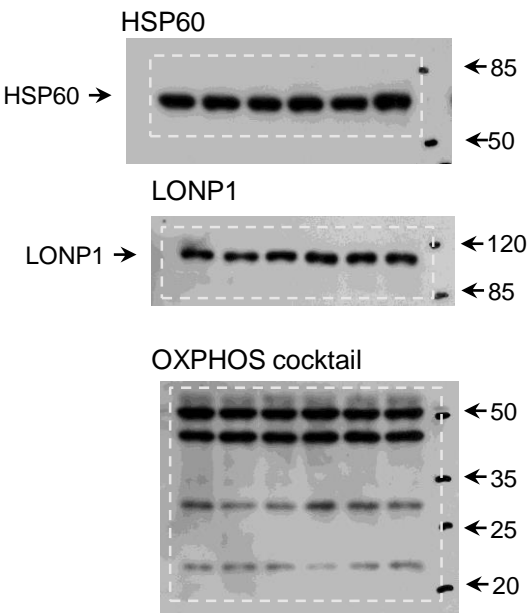

Extended Data Fig. 9g

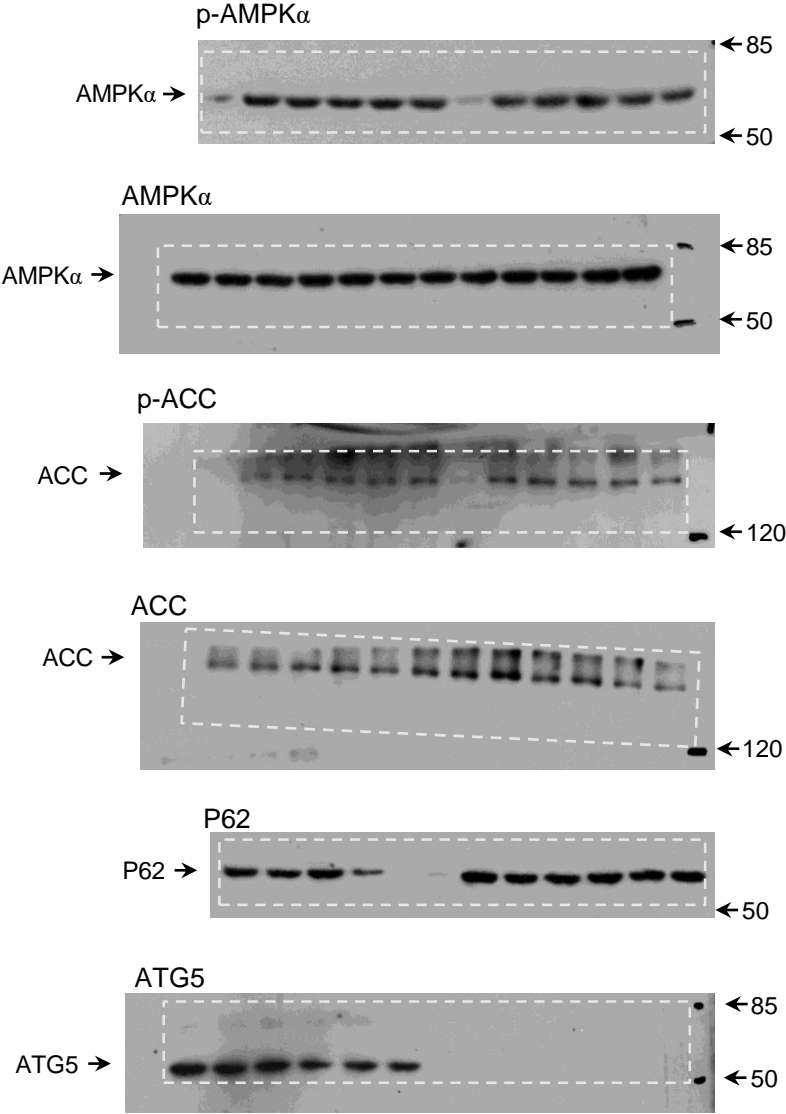

Supplement: Source Data Extended Data Fig. 9 — Unprocessed western blots. [file 42255_2022_640_MOESM31_ESM.pdf]
