# Supplementary material for: The aldolase inhibitor aldometanib mimics glucose starvation to activate lysosomal AMPK
Source: Nat Metab. 2022 Oct 10;4(10):1369–401. doi: 10.1038/s42255-022-00640-7 (PMC9584815; doi:10.1038/s42255-022-00640-7)

Extended Data Fig. 3a

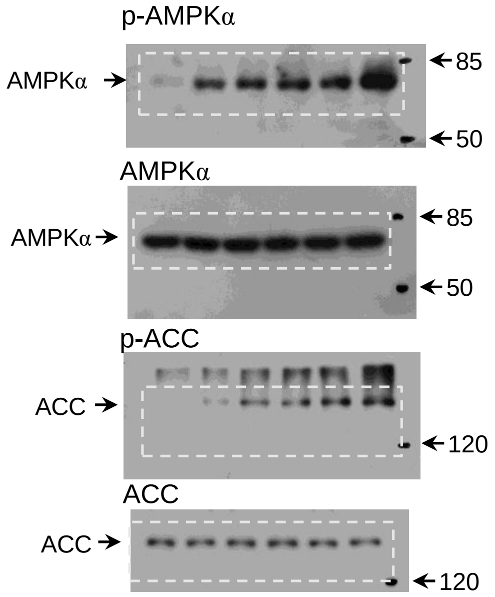

Extended Data Fig. 3b

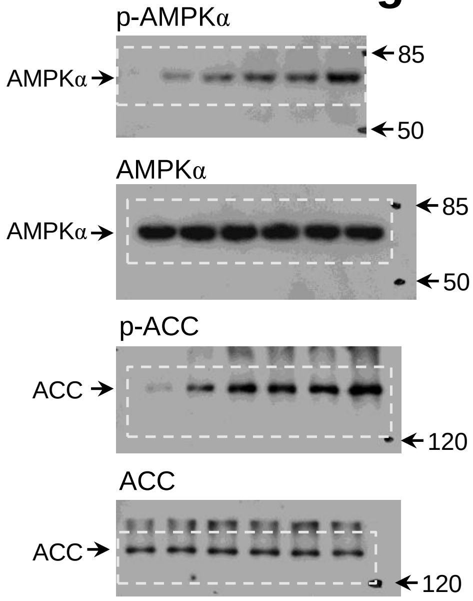

Extended Data Fig. 3c

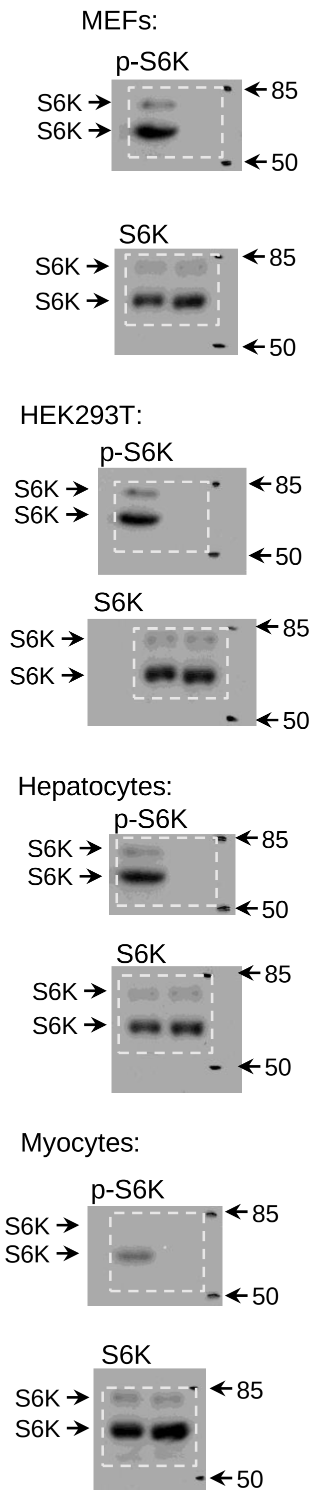

Extended Data Fig. 3d

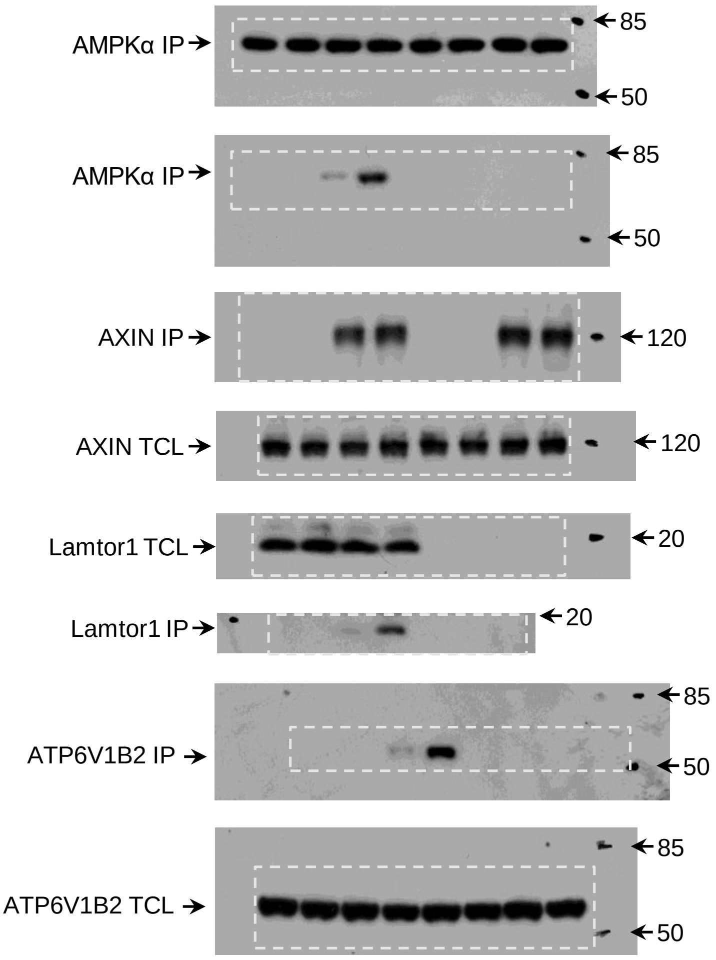

Extended Data Fig. 3e

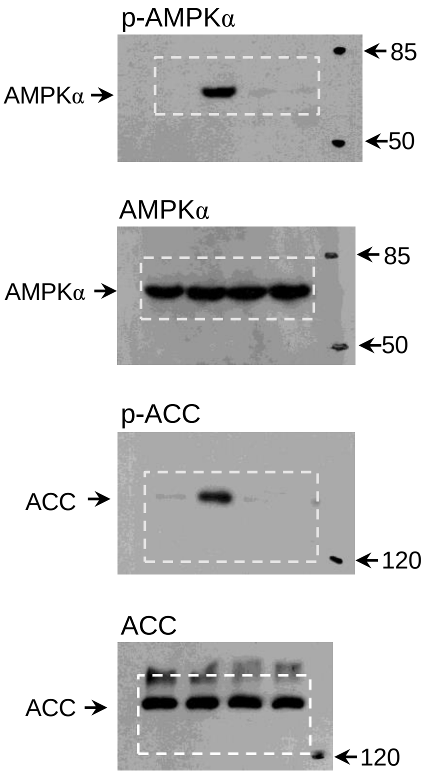

Extended Data Fig. 3f

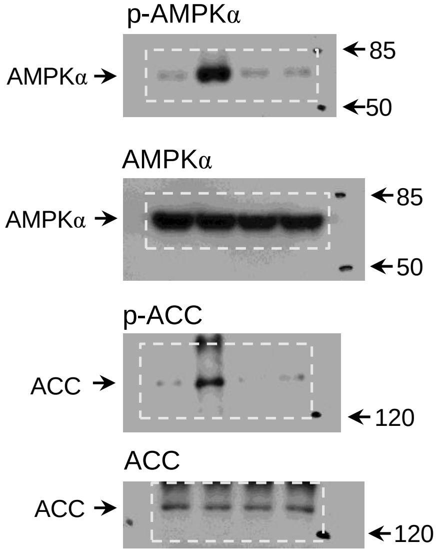

Extended Data Fig. 3g

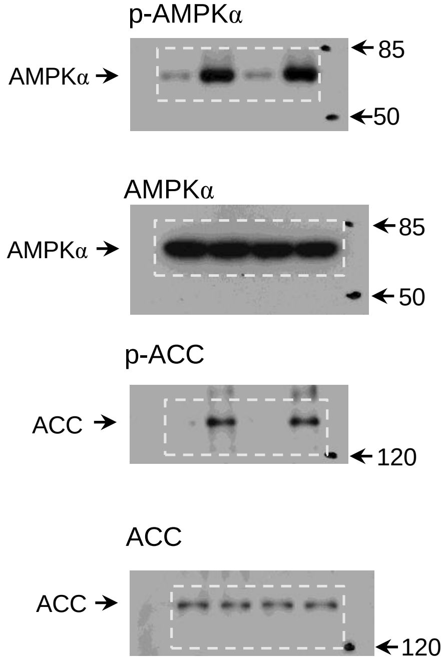

Extended Data Fig. 3i

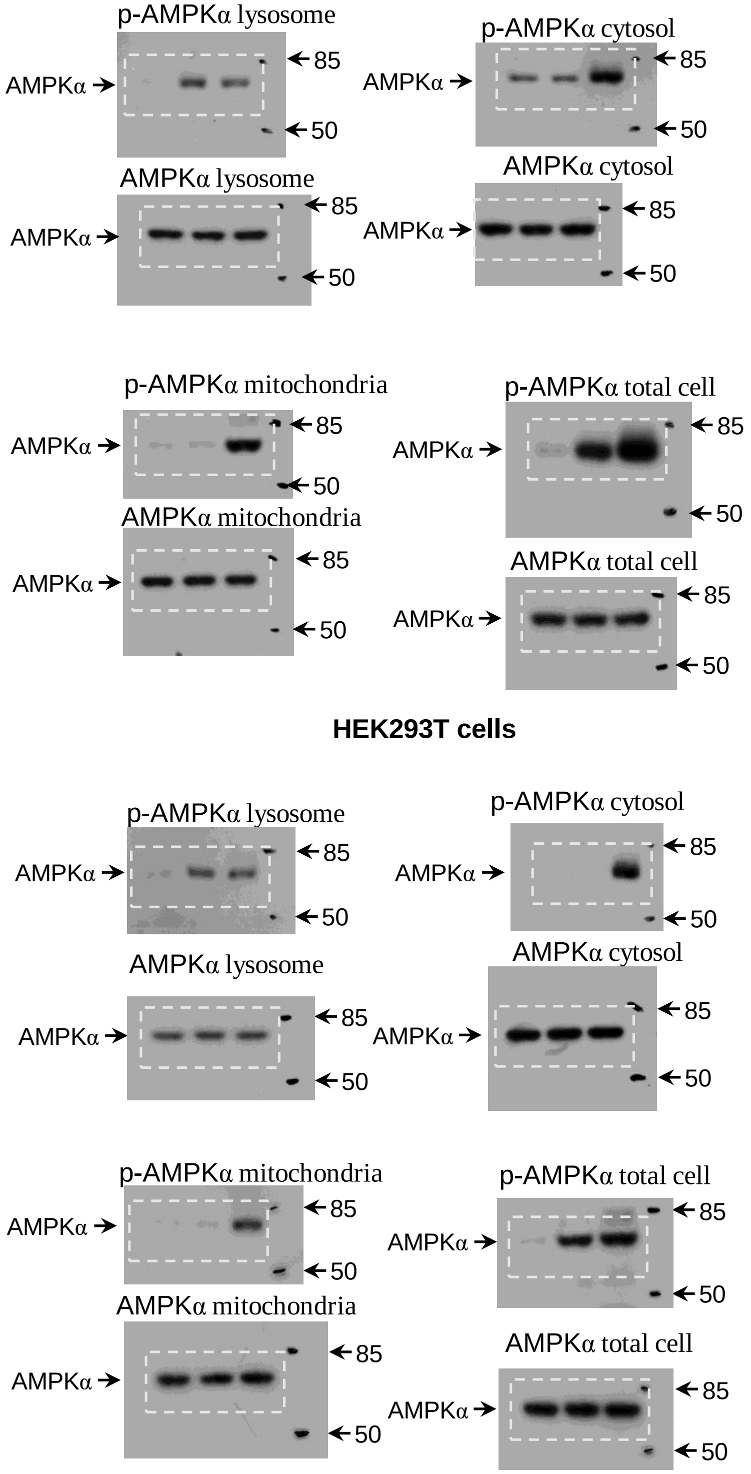

Primary skeletal muscle cells

Extended Data Fig. 3h

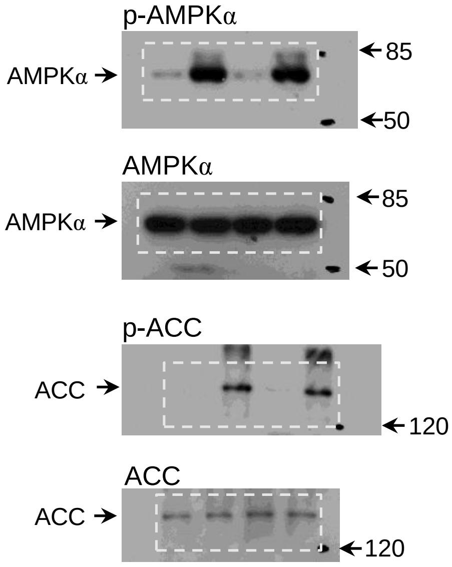

Supplement: Source Data Extended Data Fig. 3 — Unprocessed western blots. [file 42255_2022_640_MOESM19_ESM.pdf]
