# Supplementary material for: The aldolase inhibitor aldometanib mimics glucose starvation to activate lysosomal AMPK
Source: Nat Metab. 2022 Oct 10;4(10):1369–401. doi: 10.1038/s42255-022-00640-7 (PMC9584815; doi:10.1038/s42255-022-00640-7)

Extended Data Fig. 5d

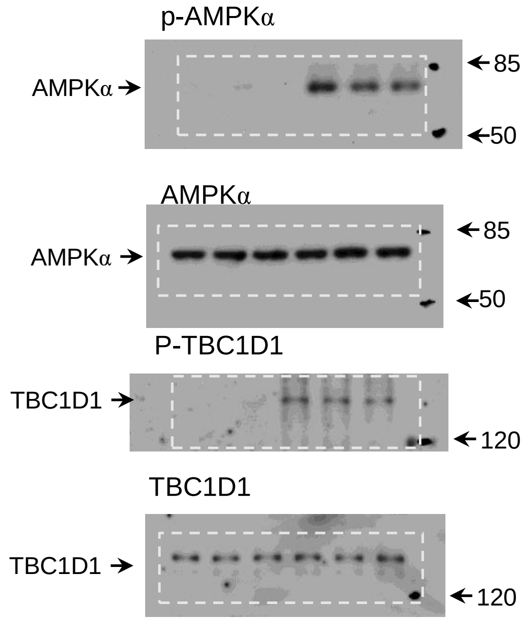

Extended Data Fig. 5e

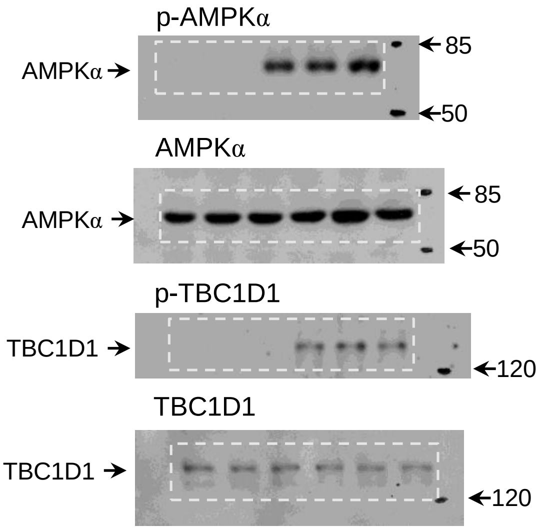

Extended Data Fig. 5g

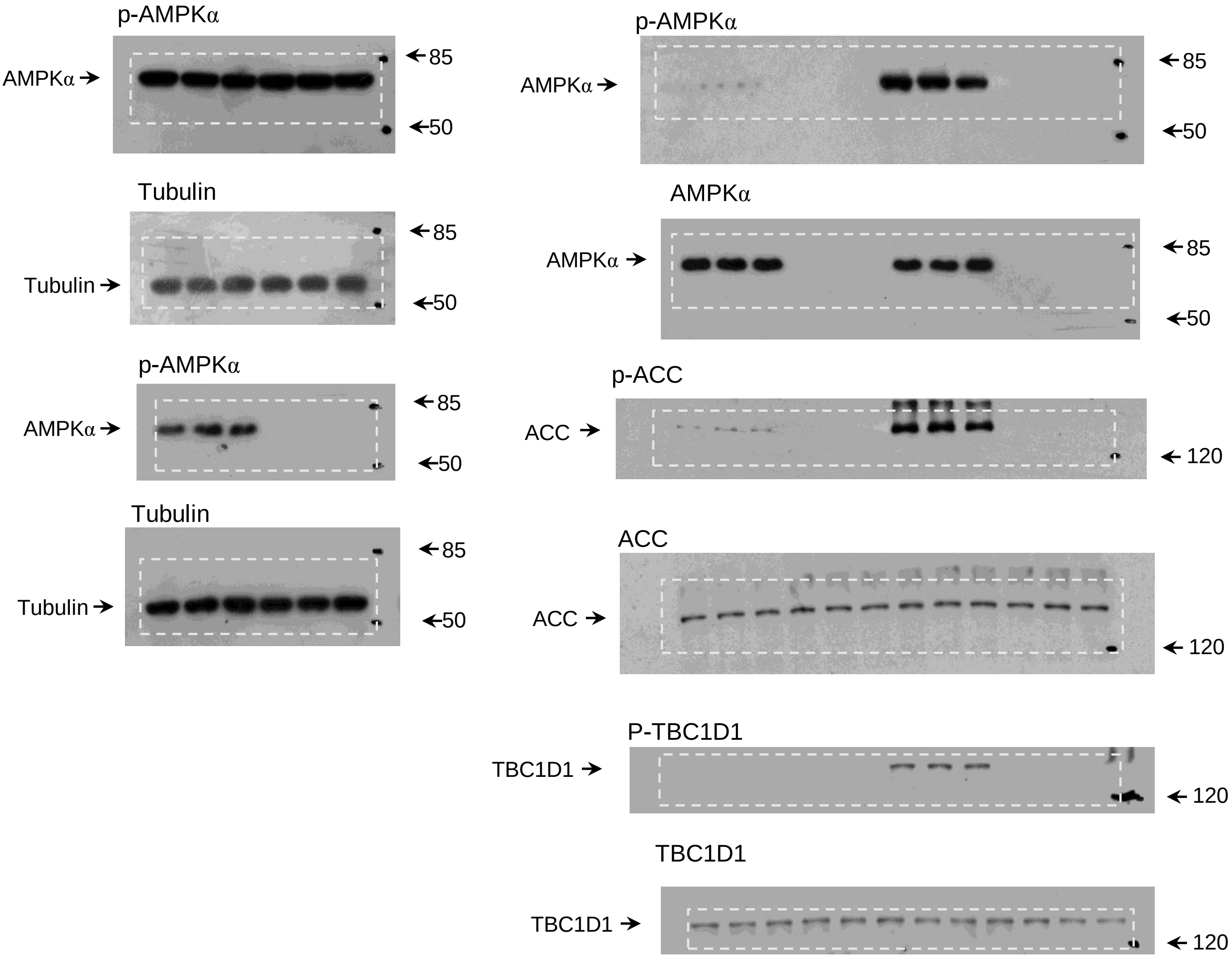

Extended Data Fig. 5g

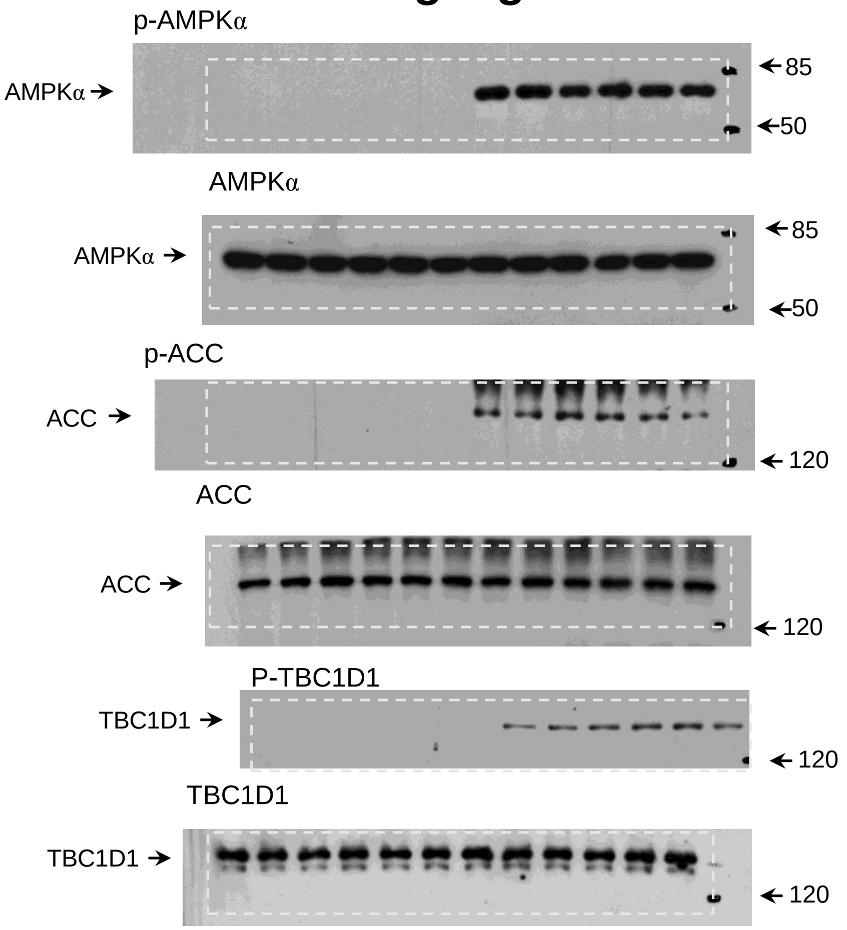

Extended Data Fig. 5i

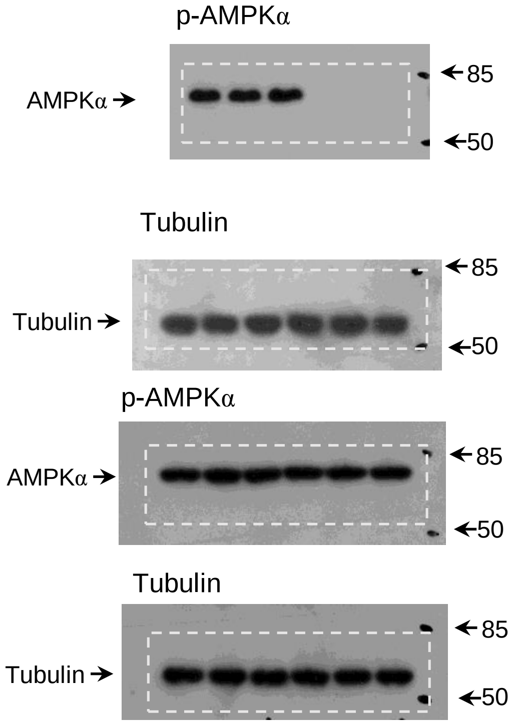

Extended Data Fig. 5j

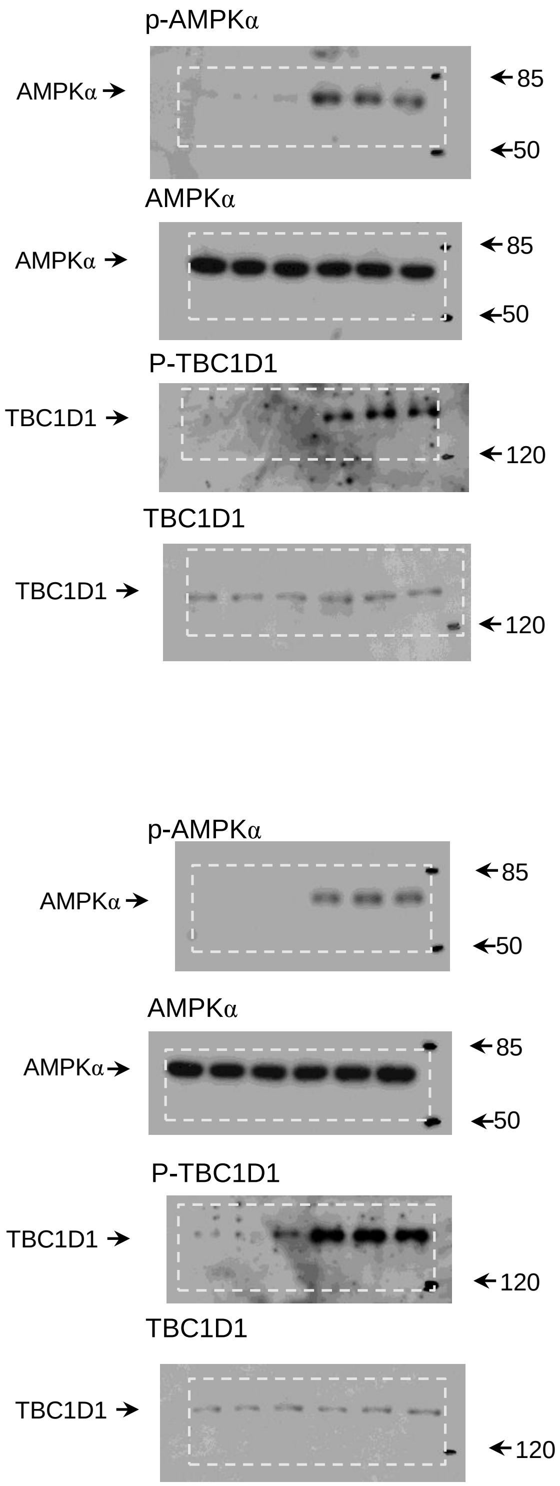

Supplement: Source Data Extended Data Fig. 5 — Unprocessed western blots. [file 42255_2022_640_MOESM23_ESM.pdf]
